# Supplementary material for: Is microfinance associated with changes in women’s well-being and children’s nutrition? A systematic review and meta-analysis
Source: BMJ Open. 2019 Jan 28;9(1):e023658. doi: 10.1136/bmjopen-2018-023658 (PMC6352765; doi:10.1136/bmjopen-2018-023658)
Supplement: Supplementary data [file bmjopen-2018-023658supp002.pdf]

Is microfinance associated with changes in women's wellbeing and childhood nutrition? A systematic review and meta-analysis

Wanjiku Gichuru[1] MPH (International Health), Shalini Ojha[2] PhD, Alan R Smyth[3] MD, Lisa Szatkowski PhD[1]

[1]University of Nottingham, Division of Epidemiology and Public Health, Clinical Sciences Building, Nottingham City Hospital, Nottingham, UK, NG5 1PB

[2]University of Nottingham, Division of Graduate Entry Medicine, Derby Medical School, Derby, DE22 3DT

[3]Child Health, Obstetrics and Gynaecology, E Floor, East Block, Queen's Medical Centre, Nottingham, UK, NG7 2UH

Corresponding author: shalini.ojha@nottingham.ac.uk

Supplementary material

Supplement 1: Search Terms

1. MEDLINE SEARCH STRATEGY

Contraceptive Use

1. (microfinanc* or microcredit or microloan or "micro-financ*" or "micro-credit" or "micro-loan" or "micro financ*" or "micro credit" or "micro loan" or "small loan" or "small lend" or "micro enterpris*" or "micro enterpreneur").mp. [mp=title, abstract, original title, name of substance word, subject heading word, keyword heading word, protocol supplementary concept word, rare disease supplementary concept word, unique identifier]
2. (random* or "randomi*ed control trial" or "randomi*ed cluster trial" or study or analys* or cohort or "cross section*" or "cross-section*" or survey or " pre test and post test" or "pre-test and post-test" or "before and after" or "interrupted time series" or "time series" or "time-series" or "time points").mp. [mp=title, abstract, original title, name of substance word, subject heading word, keyword heading word, protocol supplementary concept word, rare disease supplementary concept word, unique identifier]
3. ("contraceptive*" or "contraception" or "reproductive" or gynaecolog* or gynecolog* or "birth control" or fertility).mp. [mp=title, abstract, original title, name of substance word, subject heading word, keyword heading word, protocol supplementary concept word, rare disease supplementary concept word, unique identifier]
4. exp contraceptive behavior/
5. 3 or 4
6. economics/ or financial support/
7. 1 or 6
8. 2 and 5 and 7
9. limit 8 to (humans and yr="1990 -Current")

Female Empowerment

1. economics/ or financial support/
2. (microfinanc* or microcredit or microloan or "micro-financ*" or "micro-credit" or "micro-loan" or "micro financ*" or "micro credit" or "micro loan" or "small loan" or "small lend" or "micro enterpris*" or "micro enterpreneur").mp. [mp=title, abstract, original title, name of substance word,

subject heading word, keyword heading word, protocol supplementary concept word, rare disease supplementary concept word, unique identifier]

3. ("health" or "outcome" or "evaluat*" or "intervention" or "impact" or "result*" or "effect*").mp. [mp=title, abstract, original title, name of substance word, subject heading word, keyword heading word, protocol supplementary concept word, rare disease supplementary concept word, unique identifier]

4. (random* or "randomi*ed control trial" or "randomi*ed cluster trial" or study or analys* or cohort or "cross section*" or "cross-section*" or survey or " pre test and post test" or "pre-test and post-test" or "before and after" or "interrupted time series" or "time series" or "time-series" or "time points").mp. [mp=title, abstract, original title, name of substance word, subject heading word, keyword heading word, protocol supplementary concept word, rare disease supplementary concept word, unique identifier]

5. ("gender" or "female" or "gender violence" or "assault" or "women" or "woman" or "women's empowerment" or "empowerment" or " women's rights" or "gender equality" or "intimate partner violence" or " travel* without permission" or "girl* education" or "school enrollment" or "school enrolment" or "infanticide").mp. [mp=title, abstract, original title, name of substance word, subject heading word, keyword heading word, protocol supplementary concept word, rare disease supplementary concept word, unique identifier]

6. 1 or 2

7. 3 or 5

8. 4 and 6 and 7

9. limit 8 to (humans and yr="1990 -Current")

Nutrition

1. (microfinanc* or microcredit or microloan or "micro-financ*" or "micro-credit" or "micro-loan" or "micro financ*" or "micro credit" or "micro loan" or "small loan" or "small lend" or "micro enterpris*" or "micro enterpreneur").mp. [mp=title, abstract, original title, name of substance word, subject heading word, keyword heading word, protocol supplementary concept word, rare disease supplementary concept word, unique identifier]

2. economics/ or exp financial support/

3. Child Nutrition Disorders/ or Nutrition Disorders/ or Nutrition Surveys/

4. (nutrition or malnutrition or undernutrition or under-nutrition or "MUAC" or "mid-upper arm circumference" or "Z score*" or Z-scores or "weight-for-age" or stunting or "weight-for-height" or "weight for age" or "weight for height" or underweight or “height for age” or “height-for-age” or

wasting or “whz” or wasting).mp. [mp=title, abstract, original title, name of substance word, subject heading word, keyword heading word, protocol supplementary concept word, rare disease supplementary concept word, unique identifier]

5. (random* or "randomi*ed control trial" or "randomi*ed cluster trial" or study or analys* or cohort or "cross section*" or "cross-section*" or survey or "pre test and post test" or "pre-test and post-test" or "before and after" or "interrupted time series" or "time series" or "time-series" or "time points").mp. [mp=title, abstract, original title, name of substance word, subject heading word, keyword heading word, protocol supplementary concept word, rare disease supplementary concept word, unique identifier]

6. 1 or 2

7. 3 or 4

8. 6 and 7

9. 5 and 8

10. limit 9 to (yr="1990 -Current")

II EMBASE SEARCH STRATEGY

Contraceptive Use

1. (microfinanc* or microcredit or microloan or "micro-financ*" or "micro-credit" or "micro-loan" or "micro financ*" or "micro credit" or "micro loan" or "small loan" or "small lend" or "micro enterpris*" or "micro enterpreneur").mp. [mp=title, abstract, heading word, drug trade name, original title, device manufacturer, drug manufacturer, device trade name, keyword]

2. (random* or "randomi*ed control trial" or "randomi*ed cluster trial" or study or analys* or cohort or "cross section*" or "cross-section*" or survey or "pre test and post test" or "pre-test and post-test" or "before and after" or "interrupted time series" or "time series" or "time-series" or "time points").mp. [mp=title, abstract, heading word, drug trade name, original title, device manufacturer, drug manufacturer, device trade name, keyword]

3. exp finance/

4. 1 or 3

5. ("contraceptive*" or "contraception" or "reproductive" or gynaecolog* or gynecolog* or "birth control" or fertility).mp. [mp=title, abstract, heading word, drug trade name, original title, device manufacturer, drug manufacturer, device trade name, keyword]

6. exp contraceptive behavior/

7. 5 or 6

8. 4 and 7
9. 2 and 8
10. limit 9 to (human and yr="1990 -Current")

Female Empowerment

1. (microfinanc* or microcredit or microloan or "micro-financ*" or "micro-credit" or "micro-loan" or "micro financ*" or "micro credit" or "micro loan" or "small loan" or "small lend" or "micro enterpris*" or "micro enterpreneur").mp. [mp=title, abstract, heading word, drug trade name, original title, device manufacturer, drug manufacturer, device trade name, keyword]
2. ("health" or "outcome" or "evaluat*" or "intervention" or "impact" or "result*" or "effect*").mp. [mp=title, abstract, heading word, drug trade name, original title, device manufacturer, drug manufacturer, device trade name, keyword]
3. (random* or "randomi*ed control trial" or "randomi*ed cluster trial" or study or analys* or cohort or "cross section*" or "cross-section*" or survey or "pre test and post test" or "pre-test and post-test" or "before and after" or "interrupted time series" or "time series" or "time-series" or "time points").mp. [mp=title, abstract, heading word, drug trade name, original title, device manufacturer, drug manufacturer, device trade name, keyword]
4. ("gender" or "female" or "gender violence" or "assault" or "women" or "woman" or "women's empowerment" or "empowerment" or " women's rights" or "gender equality" or "intimate partner violence" or " travel* without permission" or "girl* education" or "school enrollment" or "school enrolment" or "infanticide").mp. [mp=title, abstract, heading word, drug trade name, original title, device manufacturer, drug manufacturer, device trade name, keyword]
5. exp finance/
6. 1 or 5
7. 2 or 4
8. 3 and 6 and 7
9. limit 8 to (human and yr="1990 -Current")
10. 1 and 3 and 7
11. limit 10 to (human and yr="1990 -Current")

Nutrition

1. (microfinanc* or microcredit or microloan or "micro-financ*" or "micro-credit" or "micro-loan" or "micro financ*" or "micro credit" or "micro loan" or "small loan" or "small lend" or "micro

enterpris*" or "micro enterpreneur").mp. [mp=title, abstract, heading word, drug trade name, original title, device manufacturer, drug manufacturer, device trade name, keyword]
2. Child Nutrition Disorders/ or Nutrition Disorders/ or Nutrition Surveys/
3. (nutrition or malnutrition or undernutrition or under-nutrition or "MUAC" or "mid-upper arm circumference" or "Z score*" or Z-scores or "weight-for-age" or stunting or "weight-for-height" or "weight for age" or "weight for height" or underweight or “height for age” or “height-for-age” or wasting or “whz” or wasting).mp. [mp=title, abstract, heading word, drug trade name, original title, device manufacturer, drug manufacturer, device trade name, keyword]
4. (random* or "randomi*ed control trial" or "randomi*ed cluster trial" or study or analys* or cohort or "cross section*" or "cross-section*" or survey or "pre test and post test" or "pre-test and post-test" or "before and after" or "interrupted time series" or "time series" or "time-series" or "time points").mp. [mp=title, abstract, heading word, drug trade name, original title, device manufacturer, drug manufacturer, device trade name, keyword]
5. 2 or 3
6. exp finance/
7. 1 or 6
8. 5 and 7
9. 4 and 8
10. limit 9 to (human and yr="1990 -Current")

III ECONLIT SEARCH STRATEGY

Female Empowerment

microfinanc* or microcredit or microloan or "micro-financ*" or "micro-credit" or "micro-loan" or "micro financ*" or "micro credit" or "micro loan" or "small loan" or "small lend" or "micro enterpris*" or "micro entrepreneur”

AND

"health" or "outcome" or "evaluat*" or "intervention" or "impact" or "result*" or "effect*" or "gender" or "female" or "gender violence" or "assault" or "women" or "woman" or "women's empowerment" or "empowerment" or " women's rights" or "gender equality" or "intimate partner violence" or " travel* without permission" or "girl* education" or "school enrollment" or "school enrolment" or "infanticide"

AND

random* or "randomi*ed control trial" or "randomi*ed cluster trial" or study or analys* or cohort or "cross section*" or "cross-section*" or survey or " pre test and post test" or "pre-test and post-test" or "before and after" or "interrupted time series" or "time series" or "time-series" or "time points”

Contraceptive Use

microfinanc* or microcredit or microloan or "micro-financ*" or "micro-credit" or "micro-loan" or "micro financ*" or "micro credit" or "micro loan" or "small loan" or "small lend" or "micro enterpris*" or "micro entrepreneur”

AND

random* or "randomi*ed control trial" or "randomi*ed cluster trial" or study or analys* or cohort or "cross section*" or "cross-section*" or survey or "pre test and post test" or "pre-test and post-test" or "before and after" or "interrupted time series" or "time series" or "time-series" or "time points”

AND

"contraceptive*" or "contraception" or "reproductive" or gynaecolog* or gynecolog* or "birth control" or fertility

Nutrition

microfinanc* or microcredit or microloan or "micro-financ*" or "micro-credit" or "micro-loan" or "micro financ*" or "micro credit" or "micro loan" or "small loan" or "small lend" or "micro enterpris*" or "micro entrepreneur”

AND

nutrition OR malnutrition OR undernutrition OR under-nutrition OR underweight OR "MUAC" OR "mid-upper arm circumference" OR stunting OR "weight-for-age" OR "height for age" OR "height-for-age" OR wasting OR whz OR "Z score"

AND

random* or "randomi*ed control trial" or "randomi*ed cluster trial" or study or analys* or cohort or "cross section*" or "cross-section*" or survey or "pre test and post test" or "pre-test and post-test" or "before and after" or "interrupted time series" or "time series" or "time-series" or "time points"

IV CENTRAL

microfinanc* or microcredit or microloan or "micro-financ*" or "micro-credit" or "micro-loan" or "micro financ*" or "micro credit" or "micro loan" or "small loan" or "small lend" or "micro enterpris*" or "micro enterpreneur"

V LILAC

Microfinan$ OR microcredit$ OR microenterprise$ OR microentrepreneur$ OR microemp$

OR

(micro AND (enterprise$ ORcredit$ OR entrepreneur$ OR finan$ OR empres$ OR companhia$))

OR

Index microfinanzas

Supplement 2: REVISED NEWCASTLE-OTTAWA SCALE ADAPTED FOR CROSS-SECTIONAL STUDIES

Selection: (Maximum 5 stars) /6

1) Representativeness of the sample: **

a) Truly representative of the average in the target population. ** (all subjects or random sampling)

b) Somewhat representative of the average in the target population. * (non-random sampling)

c) Selected group of users.

d) No description of the sampling strategy.

2) Sample size:

a) Justified and satisfactory. *

b) Not justified.

3) Non-respondents:

a) Comparability between respondents and non-respondents characteristics is established, and the response rate is satisfactory. *

b) The response rate is unsatisfactory, or the comparability between respondents and non-respondents is unsatisfactory.

c) No description of the response rate or the characteristics of the responders and the non-responders.

4) Ascertainment of the exposure (risk factor):**

a) Validated – based on individual exposure. **

b) Non-validated measurement tool, but the tool is available or described – based on group exposure e.g. village level.*

c) No description of the measurement tool.

Comparability: (Maximum 2 stars) – /2

1) The subjects in different outcome groups are comparable, based on the study design or analysis. Confounding factors are controlled.

a) The study controls for the most important factors – age, education level, social status (select one). * *

b) The study displays data on the above factors comparing intervention and non-intervention groups but does not adjust*

c) No data on above factors collected

Outcome: (Maximum 2 stars) /3

1) Assessment of the outcome:

a) Assessment through self-reported anonymised questionnaires or blinded independent assessors. **

b) Record linkage. **

c) Systematic assessment without blinding or independent assessors and self-reported through interviewer. *

d) No description

2) Statistical test: -

a) The statistical test used to analyze the data is clearly described and appropriate, and the measurement of the association is presented, including confidence intervals and the probability level (p value). *

b) The statistical test is not appropriate, not described or incomplete.

Total # of stars: /11

This scale has been adapted from the Newcastle-Ottawa Quality Assessment Scale for cohort studies to perform a quality assessment of cross-sectional studies for this systematic review.

Supplement 3: Studies Excluded at Full-Text Screening

Reason For Exclusion	Number excluded
Contraceptive Use	
No results for outcome of interest	3
No comparison group included in the study	1
Childhood Nutrition	
No results for outcome of interest	4
Inappropriate measure provided at result stage (pooled result)	1
Study results already presented in another included article	1
Not traced in print and online editions of journal referenced	3
Female Empowerment	
No non-economic outcome presented in results	29
Outcome of interest only presented for intervention group but not for comparison group	1
No comparison group included in the study	12
Comparison group included but were also exposed to the intervention in some capacity	4
Exposure included other credit sources as well as microfinance	1
No empirical quantitative data presented (theoretical framework)	3
Study results already presented in another included article (including critiques of existing studies)	3
Study protocol only, no results provided	1
Not traced in print and online editions of journal referenced	3
Primary exposure of interest not microfinance	2
Not traced in the British library catalogue and other sources	2

Supplement 4: Impact of Microfinance on Household Decision Making Agency among Female Clients

Study	Measure of Decision Making Agency	Statistical measure of effect used	Results<br>(*p*=*p-value*, *n*=sample size)	Direction of effect
Angelucci *et al, 2015*[37]	Proportion of women who participate in any financial decision<br>Number of household issues women have a say on	Regression coefficients (SE)	0.008 (0.003)<br>*p*<0.01 *n*=12183<br>0.071 (0.030)<br>*p*<0.05 *n*=12379	Positive
Beaman *et al, 2014*[38]	Proportion of women free to decide about a) food expenses b) education expenses c) business.<br><br>d) Standardised index of intra-household decision making power derived from 3 individual measures	Regression coefficients (SE) for outcomes a-c<br><br>Regression coefficient (SE) for change in standardised index d (i.e. change in deviations from mean)	-0.006 (0.016) n=5425<br>0.010 (0.014) n=4440<br>0.012 (0.020) n=4180<br>0.02 (0.03) *n*=5425	No significant change
Mohindra *et al, 2008*[39]	Decision-making agency based on at least one situation of male decision making versus no male decision making in: seeking health care of a family member; daily household expenditures; child's education at school; family planning; voting in an election.	Adjusted odds ratio for early joiner (>2 years membership) compared to non-clients	0.90 (95%CI 0.53-1.74)<br>n=928	No significant change
Montgomery & Weiss, 2011[40]	Women between 15-40 asked about their involvement in family decisions regarding:<br>Child's schooling<br>Child's marriage<br>Whether to have another child<br>Repair/construction of house<br>Sale-purchase of livestock<br>Borrowing money	Logit, SE and ORs for female clients compared to non-clients	0.22 (0.30) OR=1.25<br>0.45 (0.34) OR=1.57<br>-0.01 (0.51) OR=0.99<br>0.36 (0.40) OR=1.43<br>-0.12 (0.57) OR=0.88<br>0.96 (0.38) OR=2.62**<br>-1.16 (1.08) OR=0.31<br>-0.60 (0.60) OR=0.55<br>**significant at 5% level *n*=2876	Positive change in involvement in decisions regarding borrowing money.<br>No significant changes in other domains.

	Woman's participation in community political activity<br>Woman's decision to work outside home			
Sharif, 2004[42]	Degree of participation in decisions regarding:<br>Daily food purchases<br>Large purchases e.g. house, furniture<br>Health expenditure<br>Education of children<br>Marriage of children and social events<br>Fertility<br>Five point ranking given for each domain, 1 being least able, to 5, able to make decisions on her own	Means and standard deviation, Wilcoxon Z statistic and significance for difference between groups	Clients Non-clients<br>4.2 (1.15) 3.8 (1.45)<br>Z=1.83, $p<0.05$<br>3.1 (0.78) 2.7 (0.99)<br>Z=2.43, p<0.05<br>3.1 (0.87) 2.9 (0.91)<br>Z=0.68<br>3.2 (0.82) 2.9 (0.81)<br>Z=1.43, p<0.05<br>2.9 (0.61) 2.9 (0.67)<br>Z=2.14<br>2.9 (0.39) 2.9 (0.54)<br>Z=0.39	Positive change in decisions on purchase of food, large purchases and education of children
Tarozzi *et al, 2015*[44]	Standardised index of fraction of decision across 20 domains women involved in:<br>All issues<br>Economic issues<br>(standardised using mean and SD of the outcome estimated from control areas at endline)	Regression coefficients (SEs) for change in standardised index (i.e. change in deviations from mean)	-0.043 (0.030)<br>n=10500 women<br>-0.038 (0.032)<br>n=10497 women	No significant change
Zaman, 1999[45]	Decision making agency:<br>If owns poultry % that can sell poultry independently<br>If owns livestock % that can sell livestock independently<br>If owns jewellry % that can sell jewellry independently<br>If has savings % can use savings independently	Coefficient estimates	-0.103 (*n*=980)<br>-0.178 (*n*= 103)<br>0.017 (*n*= 694)<br>-0.345*** (*n*=379)<br><br>***significant at 1% level	Positive change only in decisions on use of savings
