# Supplementary material for: Is microfinance associated with changes in women’s well-being and children’s nutrition? A systematic review and meta-analysis
Source: BMJ Open. 2019 Jan 28;9(1):e023658. doi: 10.1136/bmjopen-2018-023658 (PMC6352765; doi:10.1136/bmjopen-2018-023658)
Supplement: Supplementary data [file bmjopen-2018-023658supp001.pdf]

PROSPERO
International prospective register of systematic reviews

National Institute for Health Research

Is microfinance associated with changes in women's empowerment and childhood nutrition, and does this vary by geographical region? A systematic review and meta-analysis

Wanjiku J Gichuru, Lisa Szatkowski, Alan Smyth, Shalini Ojha

Citation

Wanjiku J Gichuru, Lisa Szatkowski, Alan Smyth, Shalini Ojha. Is microfinance associated with changes in women's empowerment and childhood nutrition, and does this vary by geographical region? A systematic review and meta-analysis. PROSPERO 2015 CRD42015026018 Available from: http://www.crd.york.ac.uk/PROSPERO/display_record.php?ID=CRD42015026018

Review question

Through systematic review and, if possible, meta-analysis assess whether microfinance programmes are associated with changes in female empowerment and the wellbeing of women over the age of 15 years

Through systematic review and, if possible, meta-analysis assess whether microfinance programmes are associated with changes in use of a contraception method among women of reproductive age

Through systematic review and, if possible, meta-analysis assess whether microfinance programmes are associated with changes in childhood nutrition and whether this varies by the sex of the child

Searches

The Cochrane Central Register of Controlled Trials (CENTRAL),

Ovid MEDLINE,

EMBASE,

Latin American and Caribbean Health Sciences (LILACS),

ECONLIT.

An attempt will be made to access unpublished studies and dissertations through a search of grey literature through www.thesis.com.

The search will be limited to studies carried out after 1990.

No language restrictions will be imposed.

Types of study to be included

Cross-sectional surveys, cohort studies, controlled before-and-after studies, interrupted time series, quasi-experimental studies, randomised and non-randomised control/cluster trials.

Condition or domain being studied

Women's empowerment as measured by intimate partner violence, decision making agency, mobility and distinctly as uptake of a contraceptive method. Empowerment of women may also be linked to improved childhood nutrition. This will be measured by weight-for-age Z-scores, height-for-age Z-scores, weight-for-height Z scores and mid-upper arm circumference.

Participants/population

Inclusion: Women above the age of fifteen and children under-five for the outcome on childhood nutrition.

Exclusion: Men, children above five years

Intervention(s), exposure(s)

Intervention: Microfinance schemes defined as a combination of savings and credit services offered without physical collateral to a population thought to be poor or otherwise vulnerable through any organisation or institution.

The provider may be non-profit, e.g. NGO, self-help group (SHG), community-based organisation or microfinance bank, or a for-profit micro-finance institution, e.g., commercial bank.

Studies having an additional intervention will also be considered, provided that the primary intervention is microfinance.

Comparator(s)/control

Populations without any microfinance services or the same population prior to receiving microfinance. In studies with more than one comparator group, the group without microfinance will be considered as the main comparator.

Context

Developing countries in South Asia, sub Saharan Africa and Latin America and the Caribbean as defined by the Word Bank

Primary outcome(s)

1. Use of contraception

2. Childhood nutrition measured as the rate of malnutrition in girls and boys under-five years of age

3. Female empowerment and well-being

Timing and effect measures

1. Use of contraception method

2. Weight-for age Z score, Height-for-age Z score, Weight-for-height Z score, mid-upper arm circumference

3. Intimate partner violence (IPV), decision making agency,mobility

Secondary outcome(s)

None

Data extraction (selection and coding)

The search will be conducted and subsequent papers reviewed for eligibility independently by two researchers in three stages; title, abstract and full-text.

A data extraction form will be completed for each selected study by one researcher under the following sub-headings; publication details, study details, nature of study, intervention and results. The data extraction forms will then be reviewed by the second researcher. This is to be used in further analysis and synthesis of the data.

Any disparities will be solved be mutual consensus between the two primary researchers. If this is not possible, the input of the third researcher will be sought.

Risk of bias (quality) assessment

The selected studies will be assessed for risk of bias by two researchers using the Cochrane Collaboration's tool for assessing risk of bias in randomised controlled trials and for quality by the Newcastle-Ottawa Quality Assessment Scale in non-randomised studies.

Any disparities will be resolved be mutual consensus between the two primary researchers. If this is not possible, the input of the third researcher will be sought.

Strategy for data synthesis

Outcome measures will be extracted from the studies and used in the meta-analyses. The studies providing an appropriate measure of effect will be weighted using a quality rating system and then stratified by quality score. A descriptive analysis will be done for studies providing quantitative outcome measures not suitable for meta-analysis.

A fixed-effects or a random-effects model will be used in pooling of the data and a suitable method of estimating variance in studies will be applied. The summary estimate of the effect size will be done in each stratum according to quality score, i.e. high, medium and low quality score, and statistical tests (I-squared) used to check for heterogeneity.

Analysis of subgroups or subsets

A sub-group analysis of the measures of effect chosen will be done according to region to detect any variations between regions. The three regions will be geographically specified as Sub-Saharan Africa, South Asia and South America. The results will be presented by tables within the text of the review or if possible in forest plots in the meta-analysis

Contact details for further information

Dr Gichuru

wanjiku.gichuru@gmail.com

Organisational affiliation of the review

University of Nottingham

www.nottingham.ac.uk

Review team members and their organisational affiliations

Dr Wanjiku J Gichuru. University of Nottingham
Dr Lisa Szatkowski. University of Nottingham
Professor Alan Smyth. University of Nottingham
Dr Shalini Ojha. University of Nottingham

Anticipated or actual start date

20 April 2015

Anticipated completion date

08 December 2015

Funding sources/sponsors

The Commonwealth Scholars and Fellows Scheme funded the Masters' course of which this review formed part of the dissertation

Conflicts of interest

None known

Language

English

Country

England

Stage of review

Review_Completed_not_published

Subject index terms status

Subject indexing assigned by CRD

Subject index terms

Female; Humans; Nutritional Status; Power (Psychology)

Date of registration in PROSPERO

09 September 2015

Date of publication of this version

12 January 2016

Revision note for this version

Update to reflect the completion of the review.

Details of any existing review of the same topic by the same authors

Stage of review at time of this submission

Stage	Started	Completed
Preliminary searches	Yes	Yes
Piloting of the study selection process	Yes	Yes
Formal screening of search results against eligibility criteria	Yes	Yes
Data extraction	Yes	Yes
Risk of bias (quality) assessment	Yes	Yes

Stage	Started	Completed
Data analysis	Yes	Yes

Revision note

Update to reflect the completion of the review.

Versions

09 September 2015

12 January 2016

PROSPERO
This information has been provided by the named contact for this review. CRD has accepted this information in good faith and registered the review in PROSPERO. CRD bears no responsibility or liability for the content of this registration record, any associated files or external websites.
