# Supplementary material for: Is microfinance associated with changes in women’s well-being and children’s nutrition? A systematic review and meta-analysis
Source: BMJ Open. 2019 Jan 28;9(1):e023658. doi: 10.1136/bmjopen-2018-023658 (PMC6352765; doi:10.1136/bmjopen-2018-023658)
Supplement: Author's manuscript [file bmjopen-2018-023658.draft_revisions.pdf]

BMJ Open is committed to open peer review. As part of this commitment we make the peer review history of every article we publish publicly available.

When an article is published we post the peer reviewers' comments and the authors' responses online. We also post the versions of the paper that were used during peer review. These are the versions that the peer review comments apply to.

The versions of the paper that follow are the versions that were submitted during the peer review process. They are not the versions of record or the final published versions. They should not be cited or distributed as the published version of this manuscript.

BMJ Open is an open access journal and the full, final, typeset and author-corrected version of record of the manuscript is available on our site with no access controls, subscription charges or pay-per-view fees (http://bmjopen.bmj.com).

If you have any questions on BMJ Open's open peer review process please email info.bmjopen@bmj.com

Is microfinance associated with changes in women's wellbeing and childhood nutrition? A systematic review and meta-analysis

Journal:	*BMJ Open*
Manuscript ID	bmjopen-2018-023658
Article Type:	Research
Date Submitted by the Author:	18-Apr-2018
Complete List of Authors:	Gichuru, Wanjiku; University of Nottingham, Academic Division of Child Health;<br>Ojha, Shalini; University of Nottingham, Division of Graduate Entry Medicine; University of Nottingham, Academic Division of Child Health<br>Smyth, Alan; University of Nottingham, Division of Child Health, Obstetrics & Gynaecology,<br>Szatkowski, Lisa; University of Nottingham, Division of Epidemiology and Public Health
Keywords:	Microfinance, Female empowerment, Community child health < PAEDIATRICS, Global health

SCHOLARONE™
Manuscripts

Is microfinance associated with changes in women's wellbeing and childhood nutrition? A systematic review and meta-analysis

Wanjiku Gichuru[1] MPH (International Health), Shalini Ojha[2] PhD, Alan R Smyth[3] MD, Lisa Szatkowski PhD[1]

[1]University of Nottingham, Division of Epidemiology and Public Health, Clinical Sciences Building, Nottingham City Hospital, Nottingham, UK, NG5 1PB

[2]University of Nottingham, Division of Graduate Entry Medicine, Derby Medical School, Derby, DE22 3DT

[3]Division of Child Health, Obstetrics and Gynaecology, E Floor, East Block, Queen's Medical Centre, Nottingham, UK, NG7 2UH

Corresponding author: shalini.ojha@nottingham.ac.uk

Competing interests: The authors have no competing interests to declare.

For peer review only - http://bmjopen.bmj.com/site/about/guidelines.xhtml

For peer review only

ABSTRACT

Objective: To explore the impact of microfinance on contraceptive use, childhood nutrition and female empowerment in South Asia, Sub-Saharan Africa and Latin America and the Caribbean.

Setting: Communities in South Asia, Sub Saharan Africa, and Latin America

Methods: We conducted a systematic search of published and grey literature (1990-2015), with no language restrictions. We included controlled trials, observational studies, and panel data analyses investigating microfinance involving women and children in developing regions. Additional interventions were allowed provided microfinance was the primary intervention. We conducted meta-analysis, where possible, to calculate pooled odds ratios. Where studies could not be combined we described these qualitatively.

Conclusions: We included 23 studies in the review. Microfinance was associated with a 53% increase in the number of women using contraceptives [OR 1·53, 95%CI 1·21-1·94]. We found mixed results for the association between microfinance and intimate partner violence, although some positive changes were noted in overall female empowerment (e.g. decision making agency and freedom to travel). Improvements in nutrition were noted in two studies where height-for-age Z-scores (HAZ) were higher in children of clients compared to non-clients.

Interpretation: Microfinance has the potential to generate changes in contraceptive use, female empowerment and childhood nutrition. It was not possible to compare microfinance models due to the small numbers of studies. More rigorous empirical evidence is needed to establish an association between microfinance and improved social and health outcomes.

Funding: WG completed this work whilst a Master's in Public Health Student at the University of Nottingham; she received a Commonwealth Scholarship to support her study. No other funds were received.

PROSPERO registration number: CRD42015026018

For peer review only - http://bmjopen.bmj.com/site/about/guidelines.xhtml

For peer review only

Strengths of the study:

- A critical evaluation of the limited evidence of the effects of microfinance on social and health outcomes.
- Encompasses all regions of the low-and-middle income countries where microfinance is most likely to impact health and wellbeing of vulnerable populations.
- Broad search terms used to capture all types of microfinance and a range of terminologies for the chosen outcomes.
- No language restrictions – captured all Latin American literature which is vital in the field of microfinance

Limitations of the study:

- Due to lack of randomised controlled trials in this field, inclusion of quasi-experimental studies

For peer review only

For peer review only - http://bmjopen.bmj.com/site/about/guidelines.xhtml

INTRODUCTION

Rationale: Microfinance is the provision of financial services, including savings, deposit, and credit services, to the poor[1]. The term was first used in the early 1990s though schemes have been in operation in the developing world since the 1970s[2]. 'Microfinance' is subtly distinct from 'microcredit,' which refers to only small loans to poor people without a savings component. Microfinance may also include provision of micro-insurance as an "add on" to the loans and saving component. Distinct characteristics of microfinance schemes are that they are short-term, have simple application procedures and do not require loan security but instead rely on a 'collective' guarantee from an enrolled group[3]. The purpose of microfinance is that the loans should reach the poor and move them out of poverty[4].

The financial viability of microfinance programmes may be assessed by factors such as loan size, number of loans per person and repayment rates. One of the first studies to evaluate the economic impact of microfinance on participants was a quasi-experimental survey from Bangladesh[5]. This showed a reduction in moderate and extreme poverty and an increase in annual household expenditure of 18% among female, and 11% among male, borrowers. Institutions such as the World Bank, International Monetary Fund and the United Nations have since supported microfinance. There are currently over 3,500 microfinance institutions providing financial support to 170 million people worldwide, mostly in South Asia, Sub-Saharan Africa (SSA), Latin America and the Caribbean (LAC)[6].

There is an emerging body of literature, including both experimental and quasi-experimental studies, looking at the social and health outcomes of microfinance programmes. In some cases, individual studies from the same region have reported contradictory results. For example, one study in Ghana demonstrated that combining microfinance and nutritional education led to improved indicators of childhood nutrition in the intervention group[7], while a study in Ethiopia failed to demonstrate any difference in nutrition status between the children of clients and non-clients[8]. The two studies used different nutritional outcome measures as well as different age limits which makes synthesis of the findings difficult. Similarly, a study from Bangladesh reported improved female empowerment fifteen years later[9], but there was no significant effect in a study in Hyderabad, India[10]. Most available studies are small and have insufficient power to detect small changes in outcomes. Therefore, this systematic review brings together results from existing studies to assess whether

For peer review only - http://bmjopen.bmj.com/site/about/guidelines.xhtml

receiving microfinance is associated with changes in women's empowerment and the well-being of their children.

Objectives: We aimed to evaluate the impact of microfinance schemes on social outcomes, specifically female contraceptive use and measures of female empowerment (intimate partner violence, decision making ability and mobility), as well as the effects on child nutrition.

METHODS

The protocol for this review is registered with PROSPERO, registration number CRD42015026018, and is available from http://www.crd.york.ac.uk/PROSPERO.

Eligibility Criteria: We included all controlled trials, observational studies, and analyses of panel data from South Asia, SSA and LAC[11] in women over the age of 15 and children under five. We included quasi-experimental studies (empirical studies used to estimate the causal impact of an intervention without randomisation). In most cases, panel data were longitudinal or "before and after" studies. We also put in a geographical limitation to studies in countries within three World Bank regions with the highest number of developing countries[12]. Studies were included where the microfinance intervention comprised both savings and credit services, without physical collateral, to a poor or otherwise vulnerable population. Studies where microfinance was introduced and measured for expected change in outcome were included. Studies where an additional intervention was delivered in addition to microfinance were also included, provided that there was an intervention group where a microfinance intervention was assessed in comparison to the control group. In studies with more than one comparison group, the group without microfinance was considered as the main comparator. Studies were excluded where there were no suitable comparison data – either from a population who had not received microfinance, or pre-intervention data from those who went on to receive microfinance.

Outcome measures: Table 1 lists the outcome measures used to assess the impact of microfinance. The Grameen foundation proposed three variables as indicators of the social performance of microfinance[13]: female use of contraceptives, female empowerment and childhood nutrition. Three indicators of female empowerment were chosen, self-reported intimate partner violence, decision-making ability and mobility. These were collated from indicators defined by the WHO[14,15] and the UN

For peer review only - http://bmjopen.bmj.com/site/about/guidelines.xhtml

Millennium taskforce on gender equality[16] and also from literature on social measures of female empowerment[17–21]. The World Health Organisation (WHO) considers the health and wellbeing of women to be tied to their ability to access healthcare and have a say in decisions related to their health[22]. Improved health status could therefore be a possible consequence and proxy indicator of female empowerment. The WHO provides some standardised measures for use in assessing the health of women in a population. These include deaths from pregnancy-related complications, uptake of contraceptives and utilisation of perinatal services[22,23]. Uptake of contraceptives is one of the measures proposed by the Grameen Foundation. Broader measures of gender equality and female empowerment were derived from targets put forward by the UN Millennium Taskforce on MDGs. They recommended three indicators of gender equality and female empowerment to measure progress towards this goal[24] including school enrollment rates, gender gaps in pay and prevalence of gender-related violence experienced by women at the hands of intimate partners. These proposed outcome measures were used to inform the selection of the measures used in this systematic review.

Table 1: Definitions of outcome measures

**Contraceptive Use**<br>Self-reported use of any contraceptive method to prevent or plan for pregnancy.
**Female empowerment**<br>**Intimate Partner Violence (IPV):** Self-reported intimate partner violence described as physical, sexual, or psychological harm by a current or former partner[25].<br>**Sole decision-making ability:** Self-reported independent decision-making agency in household decisions where the woman is not the head of household.<br>**Mobility:** Self-reported freedom to travel out of the village or to attend social events without the permission or accompaniment of a male relative.
**Childhood nutrition**<br>Standard nutritional measures for children aged <5 as defined by the WHO Global Database on Child Growth and Malnutrition (WHO). Moderate undernutrition (malnutrition) was defined as a Z-score <-2 but > -3 standard deviations (SD) from the mean. Severe undernutrition (malnutrition) was defined as a Z-score<-3 SD from the mean.<br>**Weight-for-age Z-score (WAZ)**<br>**Height (or length)-for-age Z-score (HAZ)** – the most indicative measure of chronic undernutrition over a prolonged period leading to growth retardation known as stunting.<br>**Weight-for-height (or length) (WHZ)** – most indicative measure of acute undernutrition known as wasting. This distinguishes short children of normal weight and tall children of low weight that may not be captured by WAZ or HAZ.<br>**Body mass index (BMI)-for-age Z-score (BAZ).**

For peer review only - http://bmjopen.bmj.com/site/about/guidelines.xhtml

Mid-upper arm circumference (MUAC) – an absolute measure where a MUAC <11.5cm in children 6-60 months is considered as severe acute malnutrition (wasting) and MUAC 11-12.5cm moderate acute malnutrition[26].

Information sources:

EMBASE, MEDLINE, LILACS, CENTRAL and ECONLIT were searched from 1990 (when microfinance was first described[2] to 9th September 2015. Theses were accessed through www.theses.com, and the references of included studies were tracked to identify other relevant papers. No language restrictions were applied. Searches were conducted using MESH headings and free text, as described in Supplement 1.

Study selection, data extraction and quality assessment: Two authors (WG and LS) independently screened the titles and abstracts of retrieved studies against the study eligibility criteria. Discrepancies were resolved by discussion and duplicates removed. Retrieved studies were translated into English, where necessary, and data were extracted by the two authors independently using a standard data extraction form. The methodological quality of included studies was assessed independently by WG and LS using the Cochrane Risk-of-Bias tool[27] for controlled trials and quasi-experimental studies and a modified Newcastle Ottawa Scale (NOS)[28] for cross-sectional surveys and analyses of panel data (Supplement 2).

Data synthesis and analysis: Meta-analyses were conducted using STATA 13 (StataCorp, College Station, TX) to pool the measures of effects from eligible studies. Where available, adjusted measures of effect were preferred over unadjusted measures. Statistical significance was set at a *p*-value of <0·05. A random effects model was initially fitted for each meta-analysis. For studies with low heterogeneity analysis was repeated using a fixed effects model. Publication bias was assessed using funnel plots and Egger's asymmetry test (where at least five studies were available). Descriptive synthesis was carried out where studies could not be meta-analysed.

RESULTS

Study selection: A total of 5416 titles were identified across the three groups of outcome measures, which reduced to 4821 after removal of duplicates. From these, 4584 titles were excluded as not being on microfinance as agreed mutually by two authors; 237 abstracts were subsequently screened. A total of 17 abstracts were translated for the authors to review. Each author screened the abstracts individually then came together to compare findings. The authors disagreed on two

For peer review only - http://bmjopen.bmj.com/site/about/guidelines.xhtml

abstracts under contraceptive use, four under childhood nutrition, and 36 under female empowerment. These were discussed further jointly and agreed upon by mutual consensus. A total of 95 progressed to full-text screening. Reference tracking identified two additional studies for full-text screening. 23 articles were included in the final review (Figure 1). Seventy-four titles were excluded after full-text screening with reasons for exclusion outlined in Supplement 3. Of the 23 included articles, three reported on contraceptive use, four on childhood nutrition and sixteen on indicators of female empowerment. Fifteen were from South Asia, seven from SSA and one from LAC. Table 2 summarises the characteristics of the included studies.

For peer review only

Table 2: Summary of included studies

Author, year of publication	Study design	Study setting (urban/rural, country, region)	Number of participants included in analysis	Data collection time points	Intervention provider	Services provided	Comparison group (MF, microfinance)	Outcome measured	Quality assessment score
Studies with outcome measure of contraceptive use									
Desai & Tarozzi, 2011[29]	Panel data survey	Rural, Ethiopia, Sub-Saharan Africa	7712 women at baseline; 7949 women at follow-up	2003 and 2006	CBO supported by an international NGO	Credit and savings in group-lending model, with additional family planning (FP) education	Two comparison groups – 1. No MF or FP (used as the controls in this review) 2. FP only	Married women aged 15-49 reporting current use of any form of contraception	NOS 7/11
Pitt & Khandker, 1996[30]	Quasi-experimental using panel data	Rural, Bangladesh, South Asia	1731 women	1991, 1992	MFI - Grameen, BRAC, BRDC	Credit and savings in group lending model	No MF	Married women aged 14-50 reporting current use of any form of contraception	NOS 4/11
Steele et al, 2001[31]	Quasi-experimental using panel data	Rural, Bangladesh, South Asia	6456 women at baseline; 5696 women at follow-up	1993 and 1995	International NGO and MFI-ASA	Credit and savings in group lending model	Two comparison groups – 1. No MF (used as the controls in this review) 2. Savings with no credit	Married women reporting current use of any form of contraception	NOS 7/11
Studies with outcome measure of female empowerment									
Ahmed, 2005[32]	Data subset from cross sectional survey	Not reported, Bangladesh, South Asia	2044 women	1999	MFI - BRAC	Credit and savings in group lending model with unspecified skilled training offered to some clients	Two comparison groups – 1. No MF (used as the controls in this review) 2. Skilled training and MF	All women reporting either physical or verbal abuse between herself the client and her husband in the preceding 4 months	NOS 7/11
Bajracharya & Amin, 2013[33]	Cross sectional survey	Rural and urban, Bangladesh, South Asia	4195 women	2007 Demographic and Health Survey	Any MFI - Grameen, BRAC, ASA, PROSHIKA	Credit and Savings in group lending Model	No MF	Married women reporting any form of violence by her partner in preceding 12 months	NOS 8/11
Dalal et al, 2013[34]	Cross sectional Survey	Rural and urban, Bangladesh,	4465 women	2007 Demographic and	Any MFI - Grameen, BRAC,	Credit and Savings in group lending	No MF	All women reporting any form of violence	NOS 8/11

For peer review only - http://bmjopen.bmj.com/site/about/guidelines.xhtml

		South Asia		Health Survey	ASA, PROSHIKA	Model		by her partner in preceding 12 months	
Pronyk et al, 2006[35]	Cluster RCT	Rural, South Africa, Sub-Saharan Africa	538 women (290 intervention, 248 control)	2001, 2005	Local NGO	Credit and savings in group lending model with additional life skills training	No MF	All women reporting intimate partner violence in preceding 12 months	Cochrane Risk-of-Bias – High
Schuler et al, 1996[36]	Cross sectional survey	Rural, Bangladesh, South Asia	1225 women	1992	MFI - Grameen and BRAC	Credit and savings in group lending model	No MF	Women reporting physical beating by husband in the preceding 12 months	NOS 7/11
Angelucci et al, 2015[37]	Cluster RCT	Rural, Mexico, Latin and Central America	1823 women	2009-12	MFI – Compartamos Banco	Credit and savings in group lending model	No MF	Decision-making agency: participation in financial decisions and household issues by non-single women aged 18-60 who are not the only adult in their household.	Cochrane Risk-of-Bias – High
Banerjee et al, 2015[10]	Cluster RCT	Urban, India, South Asia	6862 women at first follow-up; 6142 women at second follow-up	2005, 2010	MFI – Spandana	Credit and savings in group lending model	No MF	Index of empowerment encompassing scores across 16 domains, covering decision making, levels of health and education expenditure and school enrollment	Cochrane Risk-of-Bias – High
Beaman et al, 2014[38]	Cluster RCT	Rural, Mali, Sub-Saharan Africa	5425 women	2009, 2012	SHG with NGO support	Credit and savings in self-help group model	No MF	Decision making agency: women's freedom to decide about food and educational expenses and take decisions about business. Index of intra-household decision making power combining individual measures	Cochrane Risk-of-Bias – High
Mohindra et al, 2008[39]	Cross sectional survey	Rural, India, South Asia	928 women	2003	SHG with NGO support	Credit and savings in self-help group model	No MF	Decision-making agency – whether women aged 18-59 reported at least 1 situation (of 5 asked) in which her husband	NOS 7/11

For peer review only

For peer review only - http://bmjopen.bmj.com/site/about/guidelines.xhtml

								or a male relative was the sole decision-maker	
Montgomery & Weiss, 2011[40]	Cross sectional survey	Rural and urban, Pakistan, South Asia	2876 women	2005	Commercial MFI - Khushali	Credit and savings in group lending model	No MF	Decision making agency – women between 15-40 asked whether their opinion is taken into account in a series of household decisions	NOS 7/11
Pitt et al, 2003[9]	Quasi-experimental using panel data	Rural, Bangladesh, South Asia	2074 women	1991/1992, 1998/1999	MFI – Grameen, BRAC, BRDC, ASA	Credit and savings in group lending model	No MF	Empowerment score combining empowerment indicators across several domains of decision making, discussion, finance and mobility	NOS 7/11
Rahman et al, 2009[41]	Quasi-experimental cross-sectional survey	Rural and urban, Bangladesh, South Asia	571 recruited and analysed	Not indicated	MFI - Grameen and BRAC	Credit and Savings in group lending model	No MF	Mobility index; empowerment index	NOS 6/11
Sharif, 2004[42]	Cross sectional survey	Not reported, Bangladesh, South Asia	483 women	1997	MFI - ASA	Credit and savings in group lending model	No MF	Decision making agency – Likert-type responses on women's extent of decision making across 6 domains	NOS 7/11
Swain & Wallentin, 2009[43]	Quasi-experimental cross-sectional survey	Rural and urban, India, South Asia	961 women	2000 and 2003	SHG with MFI linkage	Savings at group level and credit from MFI in group lending model	No MF	Empowerment score	NOS 5/11
Tarozzi et al, 2015[44]	Cluster RCT (independent baseline and follow-up samples)	Rural, Ethiopia, Sub-Saharan Africa	6412 households at baseline; 6263 households at follow-up	2003 and 2006	CBOs supported by international NGO	Credit and savings in group lending model	No MF	Decision making agency – fraction of decisions across 20 domains women aged 15-49 were involved in making	Cochrane Risk-of-Bias -High
Zaman, 1999[45]	Cross sectional survey	Rural, Bangladesh, South Asia	1568 women	1995	MFI - BRAC	Credit and savings in group lending model	No MF	Decision making agency	NOS 2/11
Studies with outcome measures of childhood nutrition									
Abubakari et al, 2014[46]	Cross sectional survey	Rural, Ghana, Sub-Saharan Africa	180 children	2011	Village Savings and Loans Association	Credit and savings in self-help group model	No MF	Anthropometric measurement of nutritional status in	NOS 4/10

For peer review only

For peer review only - http://bmjopen.bmj.com/site/about/guidelines.xhtml

								children <5 years based on HAZ scores: >-2 well nourished; <-2 to -3 moderate malnutrition; <-3 severe malnutrition	
Doocy et al, 2005[8]	Cross sectional survey	Rural and urban, Ethiopia Sub-Saharan Africa	608 children	2003	NGO - WISDOM	Credit and Savings in group lending model	Two comparison groups: 1. No MF (used as the controls in this review) 2. New clients <1 cycle of MF	Anthropometric measurement of nutritional status in children aged 6-59 months based on MUAC: <11cm severe malnutrition; 11-12.5cm moderate malnutrition	NOS 6/11
Friesen et al, 2012[47]	Cross sectional survey	Rural and urban, Ghana, Sub-Saharan Africa	204 children	June to August 2011	Local microfinance bank (previously with NGO support)	Credit and savings in group lending model	No MF	Anthropometric measurement of nutritional status in children aged 6-23 months based on proportion underweight (WAZ<-2), stunted (LAZ<-2) and wasted (WLZ<-2)	NOS 7/11
Marquis et al, 2015[7]	Quasi-experimental design with longitudinal follow-up	Rural, Ghana, Sub-Saharan Africa	608 caregivers with children	Approximately 4-monthly between April 2006 and Dec 2007	Credit and savings association	Credit and savings in self-help group model with additional health, nutrition and entrepreneur education	No MF	Anthropometric measurement of nutritional status in children aged 2-5 years based on WAZ, HAZ and BAZ scores	Cochrane Risk-of-Bias – High

*MF – Microfinance , FP- Family Planning, SHG – self-help group

For peer review only - http://bmjopen.bmj.com/site/about/guidelines.xhtml

Nature of the microfinance interventions evaluated: The most common microfinance model was group-lending as provided by formal microfinance institutions (MFIs)[9,10,30–34,36,37,41,42,45,47] and community-based organisations (CBOs)[7,8,29,35,44]. MFIs required clients to be women above the age of eighteen, own less than 0·5 decimals of land (435 square feet) and have at least one household member in casual employment. Self-help groups and CBOs had fewer eligibility criteria but with greater emphasis on accumulation of savings[7,38,39,43,46]. In some studies microfinance was coupled with additional social and health interventions[7,29,32,35].

Findings of Studies by Outcome

Contraceptive Use

Three studies[29–31] evaluated the impact of microfinance on self-reported use of contraception using data from household cross-sectional surveys. One study[29] evaluated an intervention that combined microfinance with family planning education in Ethiopia. The other two[30,31] recruited clients from non-commercial MFIs in Bangladesh.

The impact of microfinance in the Ethiopian study was estimated at the level of the *kebele* (a cluster of villages) and showed no significant change in the proportion of married women reporting contraceptive use; individual-level estimates of the impact of microfinance were not available. A fixed-effects meta-analysis of individual-level data from the two Bangladeshi studies showed that women participating in microfinance were 53% more likely to report contraceptive use than non-clients [OR=1·53, 95%CI 1·21-1·94; Figure 2]. There was no heterogeneity between the studies, which is plausible given the similarity in the average age and socio-economic status of participants.

Female empowerment

Sixteen studies evaluated the impact of microfinance on female empowerment. Eight were conventional cross-sectional studies[32–34,36,39,40,42,45], three were quasi-experimental[9,41,43] and five were cluster-randomised controlled trials (cluster RCTs)[10,35,37,38,44]. Twelve studies were from South Asia, three from SSA and one from LAC. These studies included evaluated different methods of empowerment.

Intimate partner violence (IPV): Four cross-sectional surveys[32–34,36] and one cluster RCT[35] reported this outcome. One survey[33] showed a significant 24% (95%CI 1·05-1·44) increase in odds of IPV among microfinance clients compared to non-clients. On the other hand, the cluster RCT[35]

For peer review only - http://bmjopen.bmj.com/site/about/guidelines.xhtml

demonstrated a significant decrease in IPV (adjusted risk ratio 0.45, 95%CI 0.23-0.91) and another survey[36] similarly showed reductions among clients of the two MFIs studied (OR=0.44, 95%CI 0.28-0.70 and OR=0.30, 95%CI 0.18-0.51). Dalal *et al*[34] found that microfinance clients with secondary and higher education were 2-3 times more likely to experience IPV than comparable non-clients (*p*=<0·001), while wealthier clients were twice as likely to experience IPV than comparable non-clients (*p*=<0·001); there were no changes in exposure to IPV amongst the least educated and poorest groups.

A meta-analysis was not conducted due to high heterogeneity (I^2=91·3%). This heterogeneity could have arisen because the threshold for reporting violence or the framing of the question may have differed between settings. The cluster RCT[35] was different both in design and in the add-on life skills training, which may have introduced further heterogeneity. The association between IPV and microfinance is therefore inconclusive.

Decision making agency: Seven studies were included for this outcome, five from South Asia[37,39,40,42,45] and two from SSA[38,44], with a similar number of cluster RCTs[37,38,44] and cross-sectional surveys[39,40,42,45]. This measure analysed a change from not being involved in decision making to being an active participant in household decisions. The outcome measures used were diverse and therefore unsuitable for meta-analysis. The results have been tabulated in more detail in Supplement 4 and include participation in financial and other household decisions (e.g. children's education and healthcare). Just over half the studies[37,40,42,45] showed a slightly higher degree of participation in certain household decisions by microfinance clients compared to non-clients. The other studies did not report any statistically significant changes. The impact of microfinance on women's decision making is therefore inconclusive.

***Freedom to travel (mobility)*:** In the one study that assessed mobility, non-clients were more mobile than clients in one region, but in the two other regions studied the reverse was true[41]. No formal statistical comparisons between groups were presented.

Overall empowerment: Four studies[9,10,41,43] reported an overall empowerment score, based on women's answers to questions on social and economic issues thought to have gender implications. As different statistical measures were used, meta-analysis was not possible. One study[43] demonstrated a significant increase in empowerment score over time for self-help group (SHG) members (mean difference 0.26, t=17.73, p<0.01) but no such change in the control group (mean

For peer review only - http://bmjopen.bmj.com/site/about/guidelines.xhtml

difference 0.076, t=1.71). Another[9] showed a positive and significant effect of female credit on women's overall empowerment, whilst a third[10] showed no significant changes in empowerment in women randomised to receive microfinance. The final study reported mixed results with two regions reporting higher empowerment in clients while the reverse was true in one region, though no measure of statistical significance was reported[41].

Childhood nutrition

Four studies, all from SSA, evaluated the effect of microfinance on childhood nutrition[7,8,46,47]. Three[8,46,47] were cross-sectional surveys while one[7] was a quasi-experimental study with a 16 month follow-up period. Two studies[7,47] included only children between 6-36 months of age while the other two included children under five years.

Doocy *et al* reported that children of women non-clients were 79% more likely to be wasted than children of clients (OR=1·79 95%CI 0·87-3·79)[8]. However, Friesen et al reported increased wasting among children of clients compared to non-clients (OR=1·15 95%CI 0·30-4·43)[47]. Neither association was statistically significant. As the baseline group used was different and there were no raw data available, it was not possible to recalculate the ORs for pooling by meta-analysis.

One cross-sectional study found that the prevalence of malnutrition, based on HAZ-scores, was lower amongst children of microfinance clients than those of non-clients[46]. A longitudinal study measured HAZ, WAZ and BAZ-scores every four months for 16 months[7]. The authors demonstrated a mean difference in WAZ scores of 0.28 at 8 to 12 months in favour of the intervention group and significant but smaller differences at four months and 16 months. At 16 months, HAZ-scores were significantly higher in the intervention group with a mean difference of 0·19 between the two groups. Meta-analysis was not possible as the studies used different statistical measures to present their results.

Publication Bias: A funnel plot found no evidence of publication bias in the studies that reported the impact of microfinance on IPV (Egger's test *p*-value=0·106). The possibility of publication bias could not be assessed for the other outcomes.

DISCUSSION

Summary of evidence: Table 3 summarises the impact of microfinance across the three outcome domains based on the quantitative and qualitative syntheses described above.

For peer review only - http://bmjopen.bmj.com/site/about/guidelines.xhtml

Table 3: Summary of Results of the Review

Outcome	Summary of impact of microfinance
Use of contraception	Women participating in microfinance schemes were significantly more likely to report using contraception.
Female empowerment	
Intimate partner violence	Conflicting results, with some studies reporting increased and others decreased IPV in microfinance participants.
Decision making agency	Most studies showed no effect but a minority showed a significant positive effect on some areas of decision-making.
Mobility	No statistically significant impact.
Overall empowerment score	Positive impact in two studies with mixed results and no change in two others.
Childhood nutrition	Positive impact in two of four studies, with no difference found in the remaining studies.

Fourteen of the 23 studies included in the review were from South Asia. This may limit the generalisability of the findings of this review to other geographical regions. However, this was expected as 84% of all microfinance clients are to be found in South Asia[48].

Proposed mechanisms

Microfinance (whilst primarily improving economic stability) might empower women and improve child nutrition though a number of mechanisms. A small source of income, which is available primarily to the woman in the household, could increase the "bargaining power" of female participants, in household decision making. Peer support and shared learning from other participants might have a similar effect. We have chosen the outcome measures most likely to reflect this increased bargaining power, including a woman's decisions about contraception and her feeling of empowerment. Furthermore, that women are often the primary household decision-makers on issues such as buying food (which will affect child nutrition) and on access to healthcare for children.

For peer review only - http://bmjopen.bmj.com/site/about/guidelines.xhtml

For peer review only

Contraceptive Use

Where individual-level data were available, the odds of reporting contraceptive use were higher in women participating in microfinance compared to those who did not. It has been argued that the women who self-select to join microfinance groups are more empowered than other women and this may in itself increase their likelihood of using contraception[4]. However, by comparing reported use in this group before and after the intervention[29,31], it may be possible to demonstrate any effect attributable to microfinance, even with an inherent empowered state.

Other markers of female empowerment

Gender-related violence is known to be most commonly perpetuated by a person close to the woman, usually an intimate partner[49]. Female empowerment has been tied to the ability of a woman to report and speak up against such violence. A reduction in IPV is therefore one of the expected benefits of empowerment of women through microfinance. One cluster RCT[35] reported a reduction in IPV among microfinance clients. However, the combined microfinance with life skills training may have resulted in an intervention group different from the standard client therefore limiting the generalisability of their findings. The authors of this study argued that their training empowered the women to reveal IPV, therefore reducing underreporting[35]. Underreporting of IPV is common in many studies due to its sensitive nature[50]. Studies used trained local female interviewers to limit underreporting, but despite this, the response rate to IPV questions in one study was only 41%[34]. Furthermore, women participating in microfinance may want to only highlight positive impacts of the intervention and not reveal any IPV. This raises ethical concerns that studies may fail to detect violence where it is actually present[50], compromising efforts to highlight legitimate concerns of prevailing IPV.

In most cases, the decision-making ability of women participating in microfinance was not significantly different from that of non-clients. However, most studies analysed women's perceived decision-making ability, which may be different to their actual decision-making capability. In addition, composite indices of decision-making ability make it hard to untangle any impact of microfinance on decisions which are typically male-dominated (such as child marriage and education) and decisions which are traditionally less so (such as those related to the purchase of food).

Childhood nutrition

For peer review only - http://bmjopen.bmj.com/site/about/guidelines.xhtml

Two studies[8,47] reported a lower likelihood of severe acute malnutrition in children of women participating in microfinance compared to non-participants, though were not statistically significant. Combining microfinance with nutritional education, as was the case in one study[7], showed improvement in nutritional status in children of participating care-givers than non-participating care-givers. However, it is then difficult to isolate the specific effect of microfinance. In one SHG study[46] no attempt was made to adjust for other variables, such as household resources or education status, which may be a source of confounding.

Additionally, the inclusion of HAZ scores as a measure of nutritional status[46,47] in a cross-sectional study may be misleading. Height-for-age measures the effect of poor nutrition on the growth of a child. Growth faltering is slow in reversal and requires a longer follow-up period to detect[51]. It may be more prudent to use acute measures of malnutrition such as wasting (WHZ) which are likely to be more sensitive to change in nutritional status over shorter periods.

Strengths and limitations

Five comprehensive databases were searched in this review, including a large economic database. The use of multiple indicators to measure women's empowerment and childhood nutrition also served to broaden the search to reduce the likelihood of missing relevant articles. The selection was carried out independently by two authors without any language restrictions, particularly important given the geographical regions studied.

The models used to deliver microfinance services varied across included studies. Some combined microfinance with education on family planning,[29] life skills[35] or health, nutrition and entrepreneurial skills,[7] which makes it difficult to evaluate the effect of microfinance alone. Although all interventions were taken to be similar for the purposes of this review, it is possible that the way the microfinance services were provided may have influenced the outcome. Given the small number of interventions of each type reviewed here, it is not possible to suggest a model of microfinance that is superior to others in terms of social performance.

In general, the most common source of bias in studies of the social impact of microfinance is selection bias, as participants *self-select* to either participate or not participate in the programme. Whilst a cluster RCT might guard against selection bias, a recent study[10] highlighted the current challenge in achieving randomisation due to the widespread diffusion of microfinance in some regions of South Asia leading to difficulties in identifying unexposed control clusters. Therefore, we

For peer review only - http://bmjopen.bmj.com/site/about/guidelines.xhtml

included non-randomised studies in this review in order to not limit the evidence considered. The non-randomised studies included dealt with self-selection bias in two main ways, using either panel data in a quasi-experimental design or propensity score matching (PSM). However, additional analysis in of one of the studies included in this review suggested that the reduction in intimate partner violence demonstrated using conventional statistical methods did not hold when PSM was used[33].

The average follow-up period of the studies included was three years. An alternative explanation for their statistically non-significant findings is that the observation period may have not been long enough to detect any change or may have missed any fleeting changes that occurred before the follow up survey. While changes in some measures of childhood malnutrition may be detectable within three years, changes in other outcomes requiring a shift in cultural and social norms may take much longer

Conclusions

In conclusion, our findings suggest that microfinance may be associated with increasing contraceptive use, improving female empowerment and better childhood nutrition. However, as only 5 of 23 studies included in this review were randomised trials any conclusions about direct causation must be guarded). However, the wide diversity in reported outcomes, study design, statistical methods and microfinance models makes it difficult to synthesise evaluation data statistically. Thus further studies are required to evaluate the social performance of microfinance. The design of future studies requires effective and clearly described randomisation, harmonisation of appropriate outcome measures and avoidance of confounders. Incorporating evaluation methods at the onset of a microfinance programme could help address many of the weaknesses identified here. While this may not be practical in areas where microfinance is fully established, areas with an increasing number of microfinance programmes, for example sub-Saharan Africa, would benefit.

ACKNOWLEDGEMENTS

We thank Magdalena Opazo Breton and Gabriella Zapata for their assistance in translating manuscripts written in Spanish and Portuguese.

CONTRIBUTION STATEMENT

For peer review only - http://bmjopen.bmj.com/site/about/guidelines.xhtml

WG, LS, SO and AS conceived and designed the study. WG and LS independently carried out the title, abstract and full text screening and quality assessment. WG conducted the meta-analyses and wrote the first draft of the paper. All authors critically revised subsequent drafts, and have approved the final version.

For peer review only

For peer review only - http://bmjopen.bmj.com/site/about/guidelines.xhtml

REFERENCES

1. Ledgerwood J. *Microfinance Handbook*. Washington, DC: World Bank; 1999.

2. Robinson MS. *The Microfinance Revolution: Sustainable Finance for the Poor*. Washington, DC: World Bank; 2001.

3. Schurmann AT, Johnston HB. The Group-lending Model and Social Closure: Microcredit, Exclusion, and Health in Bangladesh. *J Health Popul Nutr*. 2009;27(4):518-527.

4. Goldberg N. *Measuring the Impact of Microfinance, Taking Stock of What We Know*. Washington, DC: Grameen Foundation; 2005.

5. Pitt M, Khandker S. The Impact of Group-Based Credit Programs on Poor Households in Bangladesh: Does the Gender of Participants Matter? *J Polit Econ*. 1998;106(5):958-996.

6. MicroRate. *The State of Microfinance Investment 2013: Survey and Analysis of MIVs 8th Edition*. Lima, Peru: MicroRate; 2013. http://www.microrate.com/media/downloads/2013/11/MicroRate-The-State-of-Microfinance-Investment-2013.pdf.

7. Marquis GS, Colecraft EK, Sakyi-Dawson O, et al. An Integrated Microcredit, Entrepreneurial Training, and Nutrition Education Intervention Is Associated with Better Growth Among Preschool-Aged Children in Rural Ghana. *J Nutr*. 2015;145(2):335-343.

8. Doocy S, Teferra S, Norell D, Burnham G. Credit program outcomes: coping capacity and nutritional status in the food insecure context of Ethiopia. *Soc Sci Med*. 2005;60(10):2371-2382.

9. Pitt MM, Khandker SR, Cartwright J. *Does Micro-Credit Empower Women? Evidence from Bangladesh*. Washington, DC: World Bank; 2003.

10. Banerjee A, Duflo E, Glennerster R, Kinnan C. The miracle of microfinance? Evidence from a randomized evaluation. *Am Econ J Appl Econ*. 2015;7(1):22-53.

11. World Bank. World Bank: Countries. http://www.worldbank.org/en/country. Accessed March 3, 2016.

12. World Bank. Poverty Overview. *Poverty Overv*. 2015. http://www.worldbank.org/en/topic/poverty/overview. Accessed June 5, 2015.

13. Odell K. *Measuring the Impact of Microfinance – Taking Another Look*. Washington, DC: Grameen Foundation; 2010.

14. WHO. *Monitoring Maternal, Newborn and Child Health: Understanding Key Progress Indicators*. Geneva: World Health Organisation; 2011.

15. Murray C, Salomon J, Mathers C, Lopez A. *Summary Measures of Population Health: Concepts, Ethics, Measurement and Applications*. Geneva: World Health Organisation; 2002.

For peer review only - http://bmjopen.bmj.com/site/about/guidelines.xhtml

16. UN Millennium Project. *Investing in Development: A Practical Plan to Achieve the Millennium Development Goals*. New York: United National Development Programme; 2005.

17. Rustagi P. *Situation of Women in South Asia: Some Dimensions*. New Delhi: Centre for Women's Development Studies; 2015. w

18. UN Women. *UN Handbook for National Action Plans on Violence against Women*. New York: UN Women; 2012.

19. Kabeer N. Resources, agency, achievements: Reflections on the measurement of women's empowerment. *Dev Change*. 1999;30(3):435-464.

20. ECMIA. *Violence and Indigenous Women*. Peru: Continental Network of Indigenous Women of the Americas; 2013.

21. Meentzen A. *Gender Relationships, Changing Female Order and Identity: Social Order of Rural Peruvian Aymaras from the Female Viewpoint*. Centro Bartolomé de las Casas; 2007.

22. WHO. Monitoring maternal, newborn and child health: understanding key progress indicators. *Monit Matern Newborn Child Health Underst Key Prog Indic*. 2011.

23. Murray CL, Salomon J, Mathers C, lOPEZ A. Summary Measures of Population Health: Concepts, Ethics, Measurement and Applications. *Summ Meas Popul Health Concepts Ethics Meas Appl*. 2002.

24. UN Millennium Project 2005. Investing in Development: A Practical Plan to Achieve the Millennium Development Goals. *Invest Dev Pract Plan Achieve Millenn Dev Goals*. 2005.

25. CDC. Intimate Partner Violence. http://www.cdc.gov/violenceprevention/intimatepartnerviolence/. Accessed March 12, 2016.

26. WHO, UNICEF. *WHO Child Growth Standards and the Identification of Severe Acute Malnutrition in Infants and Children: A Joint Statement by the World Health Organization and the United Nations Children's Fund*. Geneva: WHO and UNICEF; 2009.

27. Higgins J, Green S, eds. *Cochrane Handbook for Systematic Reviews of Interventions*. Vol 5. The Cochrane Collaboration; 2011.

28. Wells G, Shea B, O'Connell D, et al. The Newcastle-Ottawa Scale (NOS) for assessing the quality of nonrandomised studies in meta-analyses.

29. Desai J, Tarozzi A. Microcredit, family planning programs, and contraceptive behavior: evidence from a field experiment in Ethiopia. *Demography*. 2011;48(2):749-782.

30. Pitt M, Khandker S. *Household and Intrahousehold Impact of the Grameen Bank and Similar Targeted Credit Programs in Bangladesh*. Washington, DC: World Bank; 1996.

31. Steele F, Amin S, Naved RT. Savings/credit group formation and change in contraception. *Demography*. 2001;38(2):267-282.

For peer review only

For peer review only - http://bmjopen.bmj.com/site/about/guidelines.xhtml

32. Ahmed SM. Intimate partner violence against women: experiences from a woman-focused development programme in Matlab, Bangladesh. *J Health Popul Nutr*. 2005;23(1):95-101.

33. Bajracharya A, Amin S. Microcredit and domestic violence in Bangladesh: an exploration of selection bias influences. *Demography*. 2013;50(5):1819-1843.

34. Dalal K, Dahlstrom O, Timpka T. Interactions between microfinance programmes and non-economic empowerment of women associated with intimate partner violence in Bangladesh: A cross-sectional study. *BMJ Open*. 2013;3(12):e002941.

35. Pronyk PM, Hargreaves JR, Kim JC, et al. Effect of a structural intervention for the prevention of intimate-partner violence and HIV in rural South Africa: a cluster randomised trial. *Lancet*. 2006;368(9551):1973-1983.

36. Schuler SR, Hashemi SM, Riley AP, Akhter S. Credit programs, patriarchy and men's violence against women in rural Bangladesh. *Soc Sci Med*. 1996;43(12):1729-1742.

37. Angelucci M, Karlan D, Zinman J. Microcredit Impacts: Evidence from a Randomized Microcredit Program Placement Experiment by Compartamos Banco. *Am Econ J Appl Econ*. 2015;7(1):151-182.

38. Beaman L, Karlan D, Thuysbaert B. *Saving for a (not So) Rainy Day: A Randomized Evaluation of Savings Groups in Mali*. Cambridge, MA: National Bureau of Economic Research; 2014.

39. Mohindra KS, Haddad S, Narayana D. Can microcredit help improve the health of poor women? Some findings from a cross-sectional study in Kerala, India. *Int J Equity Health*. 2008;7:2..

40. Montgomery H, Weiss J. Can Commercially-Oriented Microfinance Help Meet the Millennium Development Goals? Evidence from Pakistan. *World Dev*. 2011;39(1):87-109..

41. Rahman S, Junankar P, Mallik G. Factors Influencing Women's Empowerment on Microcredit Borrowers: A Case Study in Bangladesh. *Factors Influ Womens Empower Microcredit Borrowers Case Study Bangladesh*. 2009;14(3).

42. Sharif NR. Microcredit Programs and Women's Decision-Making Status: Further Evidence from Bangladesh. *Can J Dev Stud*. 2004;25(3):465-480.

43. Swain RB, Wallentin FY. Does Microfinance Empower Women? Evidence from Self-Help Groups in India. *Int Rev Appl Econ*. 2009;23(5):541-556.

44. Tarozzi A, Desai J, Johnson K. The Impacts of Microcredit: Evidence from Ethiopia. *Am Econ J Appl Econ*. 2015;7(1):54-89.

45. Zaman H. *Assessing the Impact of Micro-Credit on Poverty and Vulnerability in Bangladesh. Policy, Research Working Paper; No. WPS 2145*. Washington, DC: World Bank; 1999.

For peer review only - http://bmjopen.bmj.com/site/about/guidelines.xhtml

46. Abubakari A, Sadik BB, Keisan Y. Impact of Village Savings and Loans Associations on the Nutritional Status of Under-Five Children: A Case Study in the Sissala West District of Upper West Region. *Pak J Nutr*. 2014;13(7):390-396.

47. Friesen V, Marquis GS, Fallon K, Colecraft EK. *Tackling Poverty through Private Sector Microcredit Programs in Ghana: Does Infant and Young Child Nutrition Improve?* Montreal, Canada: McGill University; 2012.

48. Daley-Harris S. *State of the Microcredit Summit Campaign Report*. Washington, DC: Microcredit Summit Campaign; 2009.

49. Devries KM, Mak JYT, Garcia-Moreno C, et al. Global health. The global prevalence of intimate partner violence against women. *Glob Health Glob Preval Intim Partn Violence Women*. 2013;340(6140):1527-1528.

50. WHO. *Putting Women First: Ethical and Safety Recommendations for Research on Domestic Violence Against Women*. Geneva: World Health Organisation; 2001.

51. Shrimpton R, Victora CG, de Onis M, Lima RC, Blössner M, Clugston G. Worldwide timing of growth faltering: implications for nutritional interventions. *Pediatrics*. 2001;107(5):E75.

Figure legends:

Figure 1. PRISMA flow chart

Figure 2. Fixed effects meta-analysis of effect of microfinance participation on women reporting contraceptive use

For peer review only

For peer review only - http://bmjopen.bmj.com/site/about/guidelines.xhtml

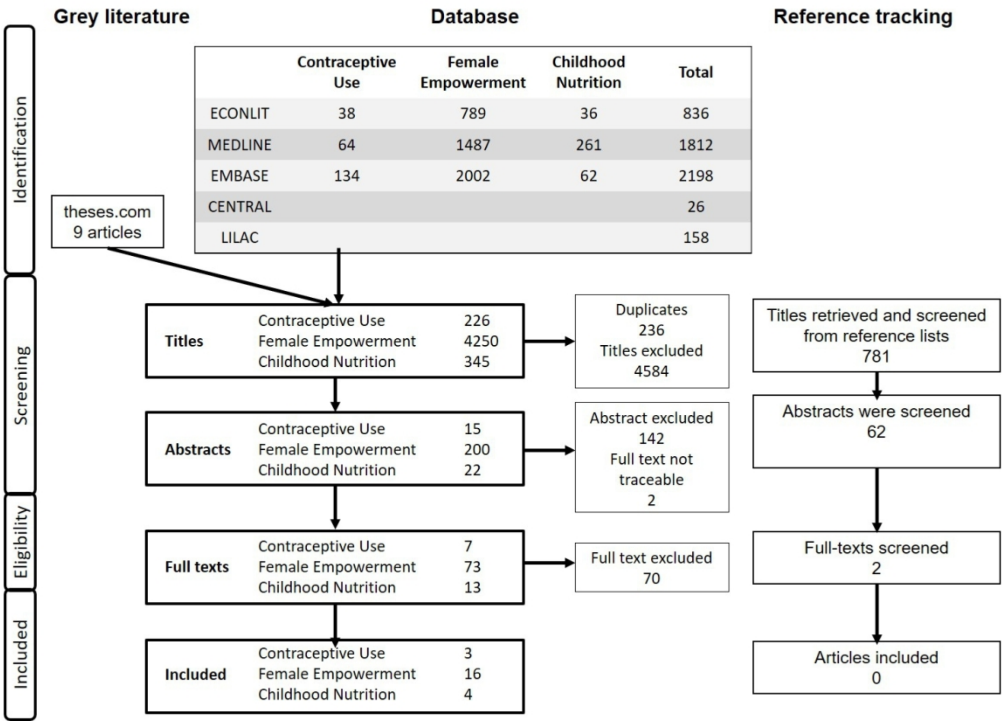

PRISMA flow chart

247x178mm (150 x 150 DPI)

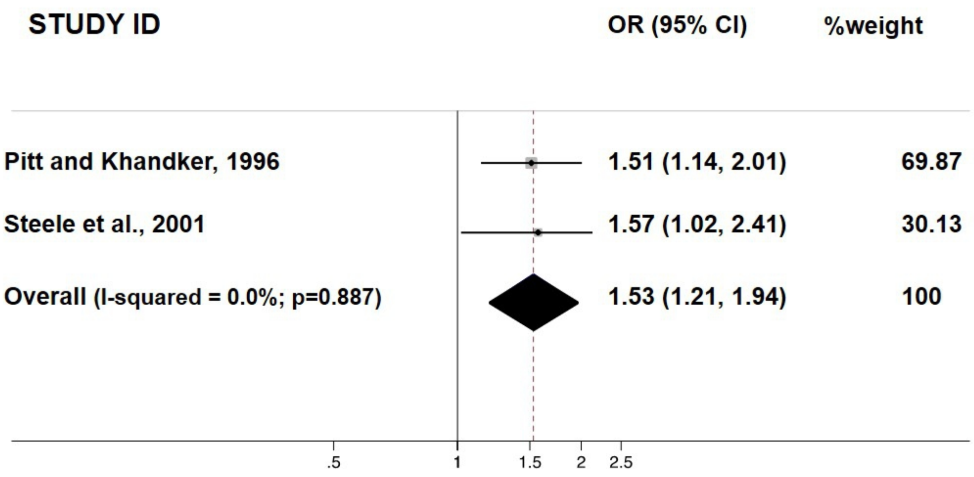

Fixed effects meta-analysis of effect of microfinance participation on women reporting contraceptive use

173x93mm (150 x 150 DPI)

Is microfinance associated with changes in women's wellbeing and childhood nutrition? A systematic review and meta-analysis

Wanjiku Gichuru[1] MPH (International Health), Shalini Ojha[2] PhD, Alan R Smyth[3] MD, Lisa Szatkowski PhD[1]

[1]University of Nottingham, Division of Epidemiology and Public Health, Clinical Sciences Building, Nottingham City Hospital, Nottingham, UK, NG5 1PB

[2]University of Nottingham, Division of Graduate Entry Medicine, Derby Medical School, Derby, DE22 3DT

[3]Child Health, Obstetrics and Gynaecology, E Floor, East Block, Queen's Medical Centre, Nottingham, UK, NG7 2UH

Corresponding author: shalini.ojha@nottingham.ac.uk

For peer review only

Supplementary material

Supplement 1: Search Terms

1. MEDLINE SEARCH STRATEGY

Contraceptive Use

1. (microfinanc* or microcredit or microloan or "micro-financ*" or "micro-credit" or "micro-loan" or "micro financ*" or "micro credit" or "micro loan" or "small loan" or "small lend" or "micro enterpris*" or "micro enterpreneur").mp. [mp=title, abstract, original title, name of substance word, subject heading word, keyword heading word, protocol supplementary concept word, rare disease supplementary concept word, unique identifier]
2. (random* or "randomi*ed control trial" or "randomi*ed cluster trial" or study or analys* or cohort or "cross section*" or "cross-section*" or survey or " pre test and post test" or "pre-test and post-test" or "before and after" or "interrupted time series" or "time series" or "time-series" or "time points").mp. [mp=title, abstract, original title, name of substance word, subject heading word, keyword heading word, protocol supplementary concept word, rare disease supplementary concept word, unique identifier]
3. ("contraceptive*" or "contraception" or "reproductive" or gynaecolog* or gynecolog* or "birth control" or fertility).mp. [mp=title, abstract, original title, name of substance word, subject heading word, keyword heading word, protocol supplementary concept word, rare disease supplementary concept word, unique identifier]
4. exp contraceptive behavior/
5. 3 or 4
6. economics/ or financial support/
7. 1 or 6
8. 2 and 5 and 7
9. limit 8 to (humans and yr="1990 -Current")

Female Empowerment

1. economics/ or financial support/
2. (microfinanc* or microcredit or microloan or "micro-financ*" or "micro-credit" or "micro-loan" or "micro financ*" or "micro credit" or "micro loan" or "small loan" or "small lend" or "micro enterpris*" or "micro enterpreneur").mp. [mp=title, abstract, original title, name of substance word,

For peer review only - http://bmjopen.bmj.com/site/about/guidelines.xhtml

subject heading word, keyword heading word, protocol supplementary concept word, rare disease supplementary concept word, unique identifier]

3. ("health" or "outcome" or "evaluat*" or "intervention" or "impact" or "result*" or "effect*").mp. [mp=title, abstract, original title, name of substance word, subject heading word, keyword heading word, protocol supplementary concept word, rare disease supplementary concept word, unique identifier]

4. (random* or "randomi*ed control trial" or "randomi*ed cluster trial" or study or analys* or cohort or "cross section*" or "cross-section*" or survey or " pre test and post test" or "pre-test and post-test" or "before and after" or "interrupted time series" or "time series" or "time-series" or "time points").mp. [mp=title, abstract, original title, name of substance word, subject heading word, keyword heading word, protocol supplementary concept word, rare disease supplementary concept word, unique identifier]

5. ("gender" or "female" or "gender violence" or "assault" or "women" or "woman" or "women's empowerment" or "empowerment" or " women's rights" or "gender equality" or "intimate partner violence" or " travel* without permission" or "girl* education" or "school enrollment" or "school enrolment" or "infanticide").mp. [mp=title, abstract, original title, name of substance word, subject heading word, keyword heading word, protocol supplementary concept word, rare disease supplementary concept word, unique identifier]

6. 1 or 2

7. 3 or 5

8. 4 and 6 and 7

9. limit 8 to (humans and yr="1990 -Current")

Nutrition

1. (microfinanc* or microcredit or microloan or "micro-financ*" or "micro-credit" or "micro-loan" or "micro financ*" or "micro credit" or "micro loan" or "small loan" or "small lend" or "micro enterpris*" or "micro enterpreneur").mp. [mp=title, abstract, original title, name of substance word, subject heading word, keyword heading word, protocol supplementary concept word, rare disease supplementary concept word, unique identifier]

2. economics/ or exp financial support/

3. Child Nutrition Disorders/ or Nutrition Disorders/ or Nutrition Surveys/

4. (nutrition or malnutrition or undernutrition or under-nutrition or "MUAC" or "mid-upper arm circumference" or "Z score*" or Z-scores or "weight-for-age" or stunting or "weight-for-height" or "weight for age" or "weight for height" or underweight or “height for age” or “height-for-age” or

For peer review only - http://bmjopen.bmj.com/site/about/guidelines.xhtml

wasting or “whz” or wasting).mp. [mp=title, abstract, original title, name of substance word, subject heading word, keyword heading word, protocol supplementary concept word, rare disease supplementary concept word, unique identifier]

5. (random* or "randomi*ed control trial" or "randomi*ed cluster trial" or study or analys* or cohort or "cross section*" or "cross-section*" or survey or "pre test and post test" or "pre-test and post-test" or "before and after" or "interrupted time series" or "time series" or "time-series" or "time points").mp. [mp=title, abstract, original title, name of substance word, subject heading word, keyword heading word, protocol supplementary concept word, rare disease supplementary concept word, unique identifier]

6. 1 or 2

7. 3 or 4

8. 6 and 7

9. 5 and 8

10. limit 9 to (yr="1990 -Current")

II EMBASE SEARCH STRATEGY

Contraceptive Use

1. (microfinanc* or microcredit or microloan or "micro-financ*" or "micro-credit" or "micro-loan" or "micro financ*" or "micro credit" or "micro loan" or "small loan" or "small lend" or "micro enterpris*" or "micro enterpreneur").mp. [mp=title, abstract, heading word, drug trade name, original title, device manufacturer, drug manufacturer, device trade name, keyword]

2. (random* or "randomi*ed control trial" or "randomi*ed cluster trial" or study or analys* or cohort or "cross section*" or "cross-section*" or survey or "pre test and post test" or "pre-test and post-test" or "before and after" or "interrupted time series" or "time series" or "time-series" or "time points").mp. [mp=title, abstract, heading word, drug trade name, original title, device manufacturer, drug manufacturer, device trade name, keyword]

3. exp finance/

4. 1 or 3

5. ("contraceptive*" or "contraception" or "reproductive" or gynaecolog* or gynecolog* or "birth control" or fertility).mp. [mp=title, abstract, heading word, drug trade name, original title, device manufacturer, drug manufacturer, device trade name, keyword]

6. exp contraceptive behavior/

7. 5 or 6

For peer review only - http://bmjopen.bmj.com/site/about/guidelines.xhtml

For peer review only

8. 4 and 7

9. 2 and 8

10. limit 9 to (human and yr="1990 -Current")

Female Empowerment

1. (microfinanc* or microcredit or microloan or "micro-financ*" or "micro-credit" or "micro-loan" or "micro financ*" or "micro credit" or "micro loan" or "small loan" or "small lend" or "micro enterpris*" or "micro enterpreneur").mp. [mp=title, abstract, heading word, drug trade name, original title, device manufacturer, drug manufacturer, device trade name, keyword]

2. ("health" or "outcome" or "evaluat*" or "intervention" or "impact" or "result*" or "effect*").mp. [mp=title, abstract, heading word, drug trade name, original title, device manufacturer, drug manufacturer, device trade name, keyword]

3. (random* or "randomi*ed control trial" or "randomi*ed cluster trial" or study or analys* or cohort or "cross section*" or "cross-section*" or survey or "pre test and post test" or "pre-test and post-test" or "before and after" or "interrupted time series" or "time series" or "time-series" or "time points").mp. [mp=title, abstract, heading word, drug trade name, original title, device manufacturer, drug manufacturer, device trade name, keyword]

4. ("gender" or "female" or "gender violence" or "assault" or "women" or "woman" or "women's empowerment" or "empowerment" or " women's rights" or "gender equality" or "intimate partner violence" or " travel* without permission" or "girl* education" or "school enrollment" or "school enrolment" or "infanticide").mp. [mp=title, abstract, heading word, drug trade name, original title, device manufacturer, drug manufacturer, device trade name, keyword]

5. exp finance/

6. 1 or 5

7. 2 or 4

8. 3 and 6 and 7

9. limit 8 to (human and yr="1990 -Current")

10. 1 and 3 and 7

11. limit 10 to (human and yr="1990 -Current")

Nutrition

1. (microfinanc* or microcredit or microloan or "micro-financ*" or "micro-credit" or "micro-loan" or "micro financ*" or "micro credit" or "micro loan" or "small loan" or "small lend" or "micro

For peer review only - http://bmjopen.bmj.com/site/about/guidelines.xhtml

enterpris*" or "micro enterpreneur").mp. [mp=title, abstract, heading word, drug trade name, original title, device manufacturer, drug manufacturer, device trade name, keyword]

2. Child Nutrition Disorders/ or Nutrition Disorders/ or Nutrition Surveys/

3. (nutrition or malnutrition or undernutrition or under-nutrition or "MUAC" or "mid-upper arm circumference" or "Z score*" or Z-scores or "weight-for-age" or stunting or "weight-for-height" or "weight for age" or "weight for height" or underweight or “height for age” or “height-for-age” or wasting or “whz” or wasting).mp. [mp=title, abstract, heading word, drug trade name, original title, device manufacturer, drug manufacturer, device trade name, keyword]

4. (random* or "randomi*ed control trial" or "randomi*ed cluster trial" or study or analys* or cohort or "cross section*" or "cross-section*" or survey or "pre test and post test" or "pre-test and post-test" or "before and after" or "interrupted time series" or "time series" or "time-series" or "time points").mp. [mp=title, abstract, heading word, drug trade name, original title, device manufacturer, drug manufacturer, device trade name, keyword]

5. 2 or 3

6. exp finance/

7. 1 or 6

8. 5 and 7

9. 4 and 8

10. limit 9 to (human and yr="1990 -Current")

III ECONLIT SEARCH STRATEGY

Female Empowerment

microfinanc* or microcredit or microloan or "micro-financ*" or "micro-credit" or "micro-loan" or "micro financ*" or "micro credit" or "micro loan" or "small loan" or "small lend" or "micro enterpris*" or "micro entrepreneur”

AND

"health" or "outcome" or "evaluat*" or "intervention" or "impact" or "result*" or "effect*" or "gender" or "female" or "gender violence" or "assault" or "women" or "woman" or "women's empowerment" or "empowerment" or " women's rights" or "gender equality" or "intimate partner violence" or " travel* without permission" or "girl* education" or "school enrollment" or "school enrolment" or "infanticide"

AND

For peer review only

random* or "randomi*ed control trial" or "randomi*ed cluster trial" or study or analys* or cohort or "cross section*" or "cross-section*" or survey or " pre test and post test" or "pre-test and post-test" or "before and after" or "interrupted time series" or "time series" or "time-series" or "time points”

Contraceptive Use

microfinanc* or microcredit or microloan or "micro-financ*" or "micro-credit" or "micro-loan" or "micro financ*" or "micro credit" or "micro loan" or "small loan" or "small lend" or "micro enterpris*" or "micro entrepreneur”

AND

random* or "randomi*ed control trial" or "randomi*ed cluster trial" or study or analys* or cohort or "cross section*" or "cross-section*" or survey or "pre test and post test" or "pre-test and post-test" or "before and after" or "interrupted time series" or "time series" or "time-series" or "time points”

AND

"contraceptive*" or "contraception" or "reproductive" or gynaecolog* or gynecolog* or "birth control" or fertility

Nutrition

microfinanc* or microcredit or microloan or "micro-financ*" or "micro-credit" or "micro-loan" or "micro financ*" or "micro credit" or "micro loan" or "small loan" or "small lend" or "micro enterpris*" or "micro entrepreneur”

AND

nutrition OR malnutrition OR undernutrition OR under-nutrition OR underweight OR "MUAC" OR "mid-upper arm circumference" OR stunting OR "weight-for-age" OR "height for age" OR "height-for-age" OR wasting OR whz OR "Z score"

AND

random* or "randomi*ed control trial" or "randomi*ed cluster trial" or study or analys* or cohort or "cross section*" or "cross-section*" or survey or "pre test and post test" or "pre-test and post-test" or "before and after" or "interrupted time series" or "time series" or "time-series" or "time points"

IV CENTRAL

microfinanc* or microcredit or microloan or "micro-financ*" or "micro-credit" or "micro-loan" or "micro financ*" or "micro credit" or "micro loan" or "small loan" or "small lend" or "micro enterpris*" or "micro enterpreneur"

For peer review only - http://bmjopen.bmj.com/site/about/guidelines.xhtml

V LILAC

Microfinan$ OR microcredit$ OR microenterprise$ OR microentrepreneur$ OR microemp$

OR

(micro AND (enterprise$ ORcredit$ OR entrepreneur$ OR finan$ OR empres$ OR companhia$))

OR

Index microfinanzas

For peer review only

For peer review only - http://bmjopen.bmj.com/site/about/guidelines.xhtml

Supplement 2: REVISED NEWCASTLE-OTTAWA SCALE ADAPTED FOR CROSS-SECTIONAL STUDIES

Selection: (Maximum 5 stars) /6

1) Representativeness of the sample: **

a) Truly representative of the average in the target population. ** (all subjects or random sampling)

b) Somewhat representative of the average in the target population. * (non-random sampling)

c) Selected group of users.

d) No description of the sampling strategy.

2) Sample size:

a) Justified and satisfactory. *

b) Not justified.

3) Non-respondents:

a) Comparability between respondents and non-respondents characteristics is established, and the response rate is satisfactory. *

b) The response rate is unsatisfactory, or the comparability between respondents and non-respondents is unsatisfactory.

c) No description of the response rate or the characteristics of the responders and the non-responders.

4) Ascertainment of the exposure (risk factor):**

a) Validated – based on individual exposure. **

b) Non-validated measurement tool, but the tool is available or described – based on group exposure e.g. village level.*

c) No description of the measurement tool.

Comparability: (Maximum 2 stars) – /2

1) The subjects in different outcome groups are comparable, based on the study design or analysis. Confounding factors are controlled.

a) The study controls for the most important factors – age, education level, social status (select one). * *

b) The study displays data on the above factors comparing intervention and non-intervention groups but does not adjust*

For peer review only - http://bmjopen.bmj.com/site/about/guidelines.xhtml

For peer review only

c) No data on above factors collected

Outcome: (Maximum 2 stars) /3

1) Assessment of the outcome:

a) Assessment through self-reported anonymised questionnaires or blinded independent assessors. **

b) Record linkage. **

c) Systematic assessment without blinding or independent assessors and self-reported through interviewer. *

d) No description

2) Statistical test: -

a) The statistical test used to analyze the data is clearly described and appropriate, and the measurement of the association is presented, including confidence intervals and the probability level (p value). *

b) The statistical test is not appropriate, not described or incomplete.

Total # of stars: /11

This scale has been adapted from the Newcastle-Ottawa Quality Assessment Scale for cohort studies to perform a quality assessment of cross-sectional studies for this systematic review.

For peer review only

Supplement 3: Studies Excluded at Full-Text Screening

Reason For Exclusion	Number excluded
Contraceptive Use	
No results for outcome of interest	3
No comparison group included in the study	1
Childhood Nutrition	
No results for outcome of interest	4
Inappropriate measure provided at result stage (pooled result)	1
Study results already presented in another included article	1
Not traced in print and online editions of journal referenced	3
Female Empowerment	
No non-economic outcome presented in results	29
Outcome of interest only presented for intervention group but not for comparison group	1
No comparison group included in the study	12
Comparison group included but were also exposed to the intervention in some capacity	4
Exposure included other credit sources as well as microfinance	1
No empirical quantitative data presented (theoretical framework)	3
Study results already presented in another included article (including critiques of existing studies)	3
Study protocol only, no results provided	1
Not traced in print and online editions of journal referenced	3
Primary exposure of interest not microfinance	2
Not traced in the British library catalogue and other sources	2

For peer review only

Supplement 4: Impact of Microfinance on Household Decision Making Agency among Female Clients

Study	Measure of Decision Making Agency	Statistical measure of effect used	Results (*p*=*p-value*, *n*=sample size)	Direction of effect
Angelucci *et al, 2015*[37]	Proportion of women who participate in any financial decision<br>Number of household issues women have a say on	Regression coefficients (SE)	0.008 (0.003)<br>*p*<0.01 *n*=12183<br>0.071 (0.030)<br>*p*<0.05 *n*=12379	Positive
Beaman *et al, 2014*[38]	Proportion of women free to decide about a) food expenses b) education expenses c) business.<br><br>d) Standardised index of intra-household decision making power derived from 3 individual measures	Regression coefficients (SE) for outcomes a-c<br><br>Regression coefficient (SE) for change in standardised index d (i.e. change in deviations from mean)	-0.006 (0.016) n=5425<br>0.010 (0.014) n=4440<br>0.012 (0.020) n=4180<br>0.02 (0.03) *n*=5425	No significant change
Mohindra *et al, 2008*[39]	Decision-making agency based on at least one situation of male decision making versus no male decision making in: seeking health care of a family member; daily household expenditures; child's education at school; family planning; voting in an election.	Adjusted odds ratio for early joiner (>2 years membership) compared to non-clients	0.90 (95%CI 0.53-1.74)<br>n=928	No significant change
Montgomery & Weiss, 2011[40]	Women between 15-40 asked about their involvement in family decisions regarding:<br>Child's schooling<br>Child's marriage<br>Whether to have another child<br>Repair/construction of house<br>Sale-purchase of livestock<br>Borrowing money	Logit, SE and ORs for female clients compared to non-clients	0.22 (0.30) OR=1.25<br>0.45 (0.34) OR=1.57<br>-0.01 (0.51) OR=0.99<br>0.36 (0.40) OR=1.43<br>-0.12 (0.57) OR=0.88<br>0.96 (0.38) OR=2.62**<br>-1.16 (1.08) OR=0.31<br>-0.60 (0.60) OR=0.55<br>**significant at 5% level *n*=2876	Positive change in involvement in decisions regarding borrowing money. No significant changes in other domains.

For peer review only - http://bmjopen.bmj.com/site/about/guidelines.xhtml

	Woman's participation in community political activity<br>Woman's decision to work outside home			
Sharif, 2004[42]	Degree of participation in decisions regarding:<br>Daily food purchases<br>Large purchases e.g. house, furniture<br>Health expenditure<br>Education of children<br>Marriage of children and social events<br>Fertility<br>Five point ranking given for each domain, 1 being least able, to 5, able to make decisions on her own	Means and standard deviation, Wilcoxon Z statistic and significance for difference between groups	Clients Non-clients<br>4.2 (1.15) 3.8 (1.45)<br>Z=1.83, p<0.05<br>3.1 (0.78) 2.7 (0.99)<br>Z=2.43, p<0.05<br>3.1 (0.87) 2.9 (0.91)<br>Z=0.68<br>3.2 (0.82) 2.9 (0.81)<br>Z=1.43, p<0.05<br>2.9 (0.61) 2.9 (0.67)<br>Z=2.14<br>2.9 (0.39) 2.9 (0.54)<br>Z=0.39	Positive change in decisions on purchase of food, large purchases and education of children
Tarozzi *et al*, *2015*[44]	Standardised index of fraction of decision across 20 domains women involved in:<br>All issues<br>Economic issues<br>(standardised using mean and SD of the outcome estimated from control areas at endline)	Regression coefficients (SEs) for change in standardised index (i.e. change in deviations from mean)	-0.043 (0.030)<br>n=10500 women<br>-0.038 (0.032)<br>n=10497 women	No significant change
Zaman, 1999[45]	Decision making agency:<br>If owns poultry % that can sell poultry independently<br>If owns livestock % that can sell livestock independently<br>If owns jewellry % that can sell jewellry independently<br>If has savings % can use savings independently	Coefficient estimates	-0.103 (*n*=980)<br>-0.178 (*n*= 103)<br>0.017 (*n*= 694)<br>-0.345*** (*n*=379)<br><br>***significant at 1% level	Positive change only in decisions on use of savings

For peer review only

For peer review only - http://bmjopen.bmj.com/site/about/guidelines.xhtml

PRISMA 2009 Checklist

Section/topic	#	Checklist item	Reported on page #
**TITLE**			
Title	1	Identify the report as a systematic review, meta-analysis, or both.	1
**ABSTRACT**			
Structured summary	2	Provide a structured summary including, as applicable: background; objectives; data sources; study eligibility criteria, participants, and interventions; study appraisal and synthesis methods; results; limitations; conclusions and implications of key findings; systematic review registration number.	2
**INTRODUCTION**			
Rationale	3	Describe the rationale for the review in the context of what is already known.	4
Objectives	4	Provide an explicit statement of questions being addressed with reference to participants, interventions, comparisons, outcomes, and study design (PICOS).	5
**METHODS**			
Protocol and registration	5	Indicate if a review protocol exists, if and where it can be accessed (e.g., Web address), and, if available, provide registration information including registration number.	5
Eligibility criteria	6	Specify study characteristics (e.g., PICOS, length of follow-up) and report characteristics (e.g., years considered, language, publication status) used as criteria for eligibility, giving rationale.	5
Information sources	7	Describe all information sources (e.g., databases with dates of coverage, contact with study authors to identify additional studies) in the search and date last searched.	6
Search	8	Present full electronic search strategy for at least one database, including any limits used, such that it could be repeated.	Supplement 1
Study selection	9	State the process for selecting studies (i.e., screening, eligibility, included in systematic review, and, if applicable, included in the meta-analysis).	7
Data collection process	10	Describe method of data extraction from reports (e.g., piloted forms, independently, in duplicate) and any processes for obtaining and confirming data from investigators.	7
Data items	11	List and define all variables for which data were sought (e.g., PICOS, funding sources) and any assumptions and simplifications made.	6
Risk of bias in individual studies	12	Describe methods used for assessing risk of bias of individual studies (including specification of whether this was done at the study or outcome level), and how this information is to be used in any data synthesis.	Supplement 2
Summary measures	13	State the principal summary measures (e.g., risk ratio, difference in means).	7

For peer review only - http://bmjopen.bmj.com/site/about/guidelines.xhtml

PRISMA 2009 Checklist

Synthesis of results	14	Describe the methods of handling data and combining results of studies, if done, including measures of consistency (e.g., I^2) for each meta-analysis.	7

Section/topic	#	Checklist item	Reported on page #
Risk of bias across studies	15	Specify any assessment of risk of bias that may affect the cumulative evidence (e.g., publication bias, selective reporting within studies).	14
Additional analyses	16	Describe methods of additional analyses (e.g., sensitivity or subgroup analyses, meta-regression), if done, indicating which were pre-specified.	7
**RESULTS**			
Study selection	17	Give numbers of studies screened, assessed for eligibility, and included in the review, with reasons for exclusions at each stage, ideally with a flow diagram.	7 [Figure 1]
Study characteristics	18	For each study, present characteristics for which data were extracted (e.g., study size, PICOS, follow-up period) and provide the citations.	8 – 11 Table 2
Risk of bias within studies	19	Present data on risk of bias of each study and, if available, any outcome level assessment (see item 12).	8 – 11 [Table 2]
Results of individual studies	20	For all outcomes considered (benefits or harms), present, for each study: (a) simple summary data for each intervention group (b) effect estimates and confidence intervals, ideally with a forest plot.	12 - 14
Synthesis of results	21	Present results of each meta-analysis done, including confidence intervals and measures of consistency.	12 [Figure 2]
Risk of bias across studies	22	Present results of any assessment of risk of bias across studies (see Item 15).	14
Additional analysis	23	Give results of additional analyses, if done (e.g., sensitivity or subgroup analyses, meta-regression [see Item 16]).	
**DISCUSSION**			
Summary of evidence	24	Summarize the main findings including the strength of evidence for each main outcome; consider their relevance to key groups (e.g., healthcare providers, users, and policy makers).	15
Limitations	25	Discuss limitations at study and outcome level (e.g., risk of bias), and at review-level (e.g., incomplete retrieval of identified research, reporting bias).	17
Conclusions	26	Provide a general interpretation of the results in the context of other evidence, and implications for future research.	18
**FUNDING**			
Funding	27	Describe sources of funding for the systematic review and other support (e.g., supply of data); role of funders for the systematic review.	2

For peer review only - http://bmjopen.bmj.com/site/about/guidelines.xhtml

PRISMA 2009 Checklist

From: Moher D, Liberati A, Tetzlaff J, Altman DG, The PRISMA Group (2009). Preferred Reporting Items for Systematic Reviews and Meta-Analyses: The PRISMA Statement. PLoS Med 6(6): e1000097. doi:10.1371/journal.pmed1000097

For more information, visit: **www.prisma-statement.org**.

For peer review only

For peer review only - http://bmjopen.bmj.com/site/about/guidelines.xhtml

BMJ Open

Is microfinance associated with changes in women's wellbeing and children's nutrition? A systematic review and meta-analysis

Journal:	*BMJ Open*
Manuscript ID	bmjopen-2018-023658.R1
Article Type:	Research
Date Submitted by the Author:	01-Aug-2018
Complete List of Authors:	Gichuru, Wanjiku; University of Nottingham, Academic Division of Child Health;<br>Ojha, Shalini; University of Nottingham, Division of Graduate Entry Medicine; University of Nottingham, Academic Division of Child Health<br>Smith, Sherie; University of Nottingham School of Medicine, Division of Child Health, Obstetrics and Gynecology<br>Smyth, Alan; University of Nottingham, Division of Child Health, Obstetrics & Gynaecology,<br>Szatkowski, Lisa; University of Nottingham, Division of Epidemiology and Public Health
**Primary Subject Heading**:	Global health
Secondary Subject Heading:	Evidence based practice, Health policy, Paediatrics
Keywords:	Microfinance, Female empowerment, Community child health < PAEDIATRICS, Global health

SCHOLARONE™
Manuscripts

Is microfinance associated with changes in women's wellbeing and children's nutrition? A systematic review and meta-analysis

Wanjiku Gichuru[1] MPH (International Health), Shalini Ojha[2] PhD, Sherie Smith[3], Alan R Smyth[3] MD, Lisa Szatkowski PhD[1]

[1]University of Nottingham, Division of Epidemiology and Public Health, Clinical Sciences Building, Nottingham City Hospital, Nottingham, UK, NG5 1PB

[2]University of Nottingham, Division of Graduate Entry Medicine, Derby Medical School, Derby, DE22 3DT

[3]Division of Child Health, Obstetrics and Gynaecology, E Floor, East Block, Queen's Medical Centre, Nottingham, UK, NG7 2UH

Corresponding author: shalini.ojha@nottingham.ac.uk

Data sharing agreement

This is a secondary analysis of published data. We do not hold any unpublished data from the study. Further information about the data analysis can be obtained by contacting the corresponding author.

For peer review only

ABSTRACT

Background: Microfinance is the provision of savings and small loans services, with no physical collateral. Most recipients are disadvantaged women. The social and health impacts of microfinance have not been comprehensively evaluated.

Objective: To explore the impact of microfinance on contraceptive use, female empowerment and children's nutrition in South Asia, Sub-Saharan Africa and Latin America and the Caribbean.

Design: We conducted a systematic search of published and grey literature (1990-2018), with no language restrictions. We conducted meta-analysis, where possible, to calculate pooled odds ratios. Where studies could not be combined we described these qualitatively.

Data Sources: EMBASE, MEDLINE, LILACS, CENTRAL and ECONLIT were searched (1990-June 2018).

Eligibility Criteria: We included controlled trials, observational studies, and panel data analyses investigating microfinance involving women and children.

Data extraction and synthesis: Two independent reviewers extracted data and assessed risk of bias. The methodological quality of included studies was assessed using the Cochrane Risk-of-Bias tool for controlled trials and quasi-experimental studies and a modified Newcastle Ottawa Scale (NOS) for cross-sectional surveys and analyses of panel data. Meta-analyses were conducted using STATA v15 (StataCorp, College Station, TX).

Results: We included 27 studies. Microfinance was associated with a 64% increase in the number of women using contraceptives [OR 1·64, 95%CI 1.45 1.86]. We found mixed results for the association between microfinance and intimate partner violence. Some positive changes were noted in female empowerment. Improvements in children's nutrition were noted in three studies.

Conclusion: Microfinance has the potential to generate changes in contraceptive use, female empowerment and children's nutrition. It was not possible to compare microfinance models due to the small numbers of studies. More rigorous evidence is needed to evaluate the association between microfinance and social and health outcomes.

Funding: WG received a Commonwealth Scholarship to support her study. There was no other funding for this work.

PROSPERO registration number: CRD42015026018

For peer review only - http://bmjopen.bmj.com/site/about/guidelines.xhtml

Strengths of the study:

A critical evaluation of the limited evidence of the effects of microfinance on social and health outcomes.

Encompasses all regions of the low-and-middle income countries where microfinance is most likely to impact health and wellbeing of vulnerable populations.

Broad search terms used to capture all types of microfinance and a range of terminologies for the chosen outcomes.

No language restrictions – captured all Latin American literature which is vital in the field of microfinance.

Limitations of the study:

We found few randomised controlled trials in the field and relied upon the inclusion of quasi-experimental studies.

For peer review only

For peer review only - http://bmjopen.bmj.com/site/about/guidelines.xhtml

INTRODUCTION

Rationale: Microfinance is the provision of financial services, including savings, deposit, and credit services, to the poor[1]. The term was first used in the early 1990s though schemes have been in operation in the developing world since the 1970s[2]. 'Microfinance' is subtly distinct from 'microcredit,' which refers to only small loans to poor people without a savings component. Microfinance may also include provision of micro-insurance as an "add on" to the loans and saving component. Distinct characteristics of microfinance schemes are that they are short-term, have simple application procedures and do not require loan security but instead rely on a 'collective' guarantee from an enrolled group[3]. The purpose of microfinance is that the loans should reach the poor and move them out of poverty[4].

The financial viability of microfinance programmes may be assessed by factors such as loan size, number of loans per person and repayment rates. One of the first studies to evaluate the economic impact of microfinance on participants was a quasi-experimental survey from Bangladesh[5]. This showed a reduction in moderate and extreme poverty and an increase in annual household expenditure of 18% among female, and 11% among male, borrowers. Institutions such as the World Bank, International Monetary Fund and the United Nations have since supported microfinance. There are currently over 3,500 microfinance institutions providing financial support to 170 million people worldwide, mostly in South Asia, Sub-Saharan Africa (SSA), Latin America and the Caribbean (LAC)[6] .

There is an emerging body of literature, including both experimental and quasi-experimental studies, looking at the social and health outcomes of microfinance programmes. In some cases, individual studies from the same region have reported contradictory results. For example, one study in Ghana demonstrated that combining microfinance and nutritional education led to improved indicators of children's nutrition in the intervention group[7], while a study in Ethiopia failed to demonstrate any difference in nutrition status between the children of clients and non-clients[8]. The two studies used different nutritional outcome measures as well as different age limits which makes synthesis of the findings difficult. Similarly, a study from Bangladesh reported improved female empowerment fifteen years later[9], but there was no significant effect in a study in Hyderabad, India[10]. Most available studies are small and have insufficient power to detect small changes in outcomes. Therefore, this systematic review brings together results from existing studies to assess whether receiving microfinance is associated with changes in women's empowerment and the well-being of their children.

Objectives: We aimed to evaluate the impact of microfinance schemes on health and social outcomes, specifically female contraceptive use and measures of female empowerment (intimate partner violence, decision making ability and mobility), as well as the effects on child nutrition.

For peer review only - http://bmjopen.bmj.com/site/about/guidelines.xhtml

METHODS

The protocol for this review is registered with PROSPERO, registration number CRD42015026018, and is available from http://www.crd.york.ac.uk/PROSPERO.

Eligibility Criteria: We included all controlled trials, observational studies, and analyses of panel data from South Asia, SSA and LAC[11] in women over the age of 15 and children under five. We included quasi-experimental studies (empirical studies used to estimate the causal impact of an intervention without randomisation). In most cases, panel data were longitudinal or "before and after" studies. We also put in a geographical limitation to studies in countries within three World Bank regions with the highest number of developing countries[12]. Studies were included where the microfinance intervention comprised both savings and credit services, without physical collateral, to a poor or otherwise vulnerable population. Studies where microfinance was introduced and measured for expected change in outcome were included. Studies where an additional intervention was delivered in addition to microfinance were also included, provided that there was an intervention group where a microfinance intervention was assessed in comparison to the control group. In studies with more than one comparison group, the group without microfinance was considered as the main comparator. Studies were excluded where there were no suitable comparison data – either from a population who had not received microfinance, or pre-intervention data from those who went on to receive microfinance.

Patient and Public Involvement: There was no PPI involvement in the design or conduct of this review. The results were presented and discussed at a dissemination workshop in Patna, Bihar.

We conducted a workshop "Women's Empowerment and Child Health: Exploring the Impact of Rojiroti Microfinance in Poor Communities in Bihar- An Indo-UK collaboration" in Patna, India on May 22, 2018. It was attended by more than 30 women who participate in microfinance, and a wide range of local stakeholders. The results of this review and other work were presented and discussed it this meeting and women's views were noted to enable further research in this area.

Outcome measures: Table 1 lists the outcome measures used to assess the impact of microfinance. The Grameen foundation proposed three variables as indicators of the social performance of microfinance[13]: female use of contraceptives, female empowerment and children's nutrition. [14-19]

The World Health Organisation (WHO) considers the health and wellbeing of women to be tied to their ability to access healthcare and have a say in decisions related to their health[14]. Improved health status could therefore be a possible consequence and proxy indicator of female empowerment. The WHO provides some standardised measures for use in assessing the health of women in a population. These include deaths from pregnancy-related complications, uptake of contraceptives and utilisation of perinatal services[14 15]. Uptake of contraceptives is one of the measures proposed by the Grameen Foundation. [16]

For peer review only - http://bmjopen.bmj.com/site/about/guidelines.xhtml

Due to the broadness of the term "female empowerment", indicators collated from definitions used by the WHO[14 15] and the UN Millennium taskforce on gender equality[16] and also from literature on social measures of female empowerment[17 19] were used to inform the selection of the three outcome measures of female empowerment used in this systematic review. These were self-reported intimate partner violence, decision-making ability and mobility.

Table 1: Definitions of outcome measures

Contraceptive Use

Self-reported use of any contraceptive method to prevent or plan for pregnancy.

Female empowerment

Intimate Partner Violence (IPV): Self-reported intimate partner violence described as physical, sexual, or psychological harm by a current or former partner[20].

Sole decision-making ability: Self-reported independent decision-making ability where the woman is not the head of household; including but not limited to, household expenditure, children's education or as a combined measure of empowerment as defined by individual study authors.

Mobility: Self-reported freedom to travel out of the village or to attend social events without the permission or accompaniment of a male relative.

Children's nutrition

Standard nutritional measures for children aged <5 as defined by the WHO Global Database on Child Growth and Malnutrition (WHO). Moderate undernutrition (malnutrition) was defined as a Z-score <-2 but > -3 standard deviations (SD) from the mean. Severe undernutrition (malnutrition) was defined as a Z-score<-3 SD from the mean.

Weight-for-age Z-score (WAZ)

Height (or length)-for-age Z-score (HAZ) – the most indicative measure of chronic undernutrition over a prolonged period leading to growth retardation known as stunting.

Weight-for-height (or length) (WHZ) – most indicative measure of acute undernutrition known as wasting. This distinguishes short children of normal weight and tall children of low weight that may not be captured by WAZ or HAZ.

Body mass index (BMI)-for-age Z-score (BAZ).

Mid-upper arm circumference (MUAC) – an absolute measure where a MUAC <11.5cm in children 6-60 months is considered as severe acute malnutrition (wasting) and MUAC 11-12.5cm moderate acute malnutrition[26].

Information sources:

EMBASE, MEDLINE, LILACS, CENTRAL and ECONLIT were searched from 1990 (when microfinance was first described[2] to 9th September 2015. These were accessed through www.theses.com, and the references of included studies were tracked to identify other relevant papers. No language restrictions were applied. Searches were conducted using MESH headings and free text, as described in Supplement 1.

For peer review only - http://bmjopen.bmj.com/site/about/guidelines.xhtml

Study selection, data extraction and quality assessment: Two authors (WG and LS) independently screened the titles and abstracts of retrieved studies against the study eligibility criteria. The search was updated in June 2018. For the updated search, two authors again screened the titles and abstracts (SS and SO) of the retrieved studies and two authors (SS and WG) screened the full text and extracted data, where possible. Discrepancies were resolved by discussion and duplicates removed. Retrieved studies were translated into English, where necessary, and data were extracted by the two authors independently using a standard data extraction form. The methodological quality of included studies was assessed independently by WG and LS using the Cochrane Risk-of-Bias tool[21] for controlled trials and quasi-experimental studies and a modified Newcastle Ottawa Scale (NOS)[22] for cross-sectional surveys and analyses of panel data (Supplement 2).

Data synthesis and analysis: Meta-analyses were conducted using STATA v15 (StataCorp, College Station, TX) to pool the measures of effects from eligible studies. Where available, adjusted measures of effect were preferred over unadjusted measures. Statistical significance was set at a *p*-value of <0·05. A random effects model was initially fitted for each meta-analysis. For studies with low heterogeneity analysis was repeated using a fixed effects model. Publication bias was assessed using funnel plots and Egger's asymmetry test (where at least five studies were available). Descriptive synthesis was carried out where studies could not be meta-analysed.

For peer review only

RESULTS

Study selection: A total of 5659 titles were identified across the three groups of outcome measures, which reduced to 5298 after removal of duplicates. From these, 5023 titles were excluded as not being on microfinance as agreed mutually by two authors; 275 abstracts were subsequently screened. A total of 17 abstracts were translated for the authors to review. Each author screened the abstracts individually then came together to compare findings. The authors disagreed on 2 abstracts under contraceptive use, 4 under children's nutrition, and 36 under female empowerment. These were discussed further jointly and agreed upon by mutual consensus. A total of 97 progressed to full-text screening. Reference tracking identified 2 additional studies for full-text screening. We included 27 articles in the final review (Figure 1). Seventy titles were excluded after full-text screening with reasons for exclusion outlined in Supplement 3. Of the 27 included articles, 4 reported on contraceptive use, 5 on childrens' nutrition and 18 on indicators of female empowerment. Eighteen were from South Asia, 8 from SSA and 1 from LAC. Table 2 summarises the characteristics of the included studies.

For peer review only - http://bmjopen.bmj.com/site/about/guidelines.xhtml

Table 2: Summary of included studies

Author, year of publication	Study design	Study setting (urban/rural, country, region)	Number of participants included in analysis	Data collection time points	Intervention provider	Services provided	Comparison group (MF, microfinance)	Outcome measured	Quality assessment score
**Studies with outcome measure of contraceptive use**									
Desai & Tarozzi, 2011[23]	Baseline and follow up surveys from a panel of villages: the impact of the program was estimated using a difference-in difference approach	Rural, Ethiopia, Sub-Saharan Africa	7712 women at baseline; 7949 women at follow-up	2003 and 2006	CBO supported by an international NGO	Credit and savings in group-lending model, with additional family planning (FP) education	Two comparison groups – 1. No MF or FP (used as the controls in this review) 2. FP only	Married women aged 15-49 reporting current use of any form of contraception	NOS 7/11
Pitt & Khandker, 1996[24]	Quasi-experimental study using an econometric approach to account for non-random placement of credit programs and unmeasured village and household attributes	Rural, Bangladesh, South Asia	1731 women	1991, 1992	MFI - Grameen, BRAC, BRDC	Credit and savings in group lending model	No MF	Married women aged 14-50 reporting current use of any form of contraception	NOS 4/11
Steele et al, 2001[25]	Quasi-experimental study. Analysis accounted for non-random placement and self-selection by taking a random sample of women and classifying them according to their eligibility for program membership to form target and non-target groups and considered demographic and socioeconomic variables in the analysis	Rural, Bangladesh, South Asia	6456 women at baseline; 5696 women at follow-up	1993 and 1995	International NGO and MFI-ASA	Credit and savings in group lending model	Two comparison groups – 1. No MF (used as the controls in this review) 2. Savings with no credit	Married women reporting current use of any form of contraception	NOS 7/11
Murshid &Ely 2017[26]	Quasi-experimental study – a logistic regression model adjusted for socio-economic variables	Rural, Bangladesh, South Asia	7325 women	2011	Grameen, BRAC, ASA, Proshika, Mother's Club, BRDB or other	Credit and savings in group lending model	Non participants	Married women aged 14-50 reporting any form of contraception	NOS 7/11
**Studies with outcome measure of female empowerment**									
Ahmed, 2005[27]	Data subset from cross sectional survey.	Not reported, Bangladesh,	2044 women	1999	MFI - BRAC	Credit and savings in group lending	Two comparison groups –	All women reporting either physical or	NOS 7/11

For peer review only - http://bmjopen.bmj.com/site/about/guidelines.xhtml

	Conduced bivariate analysis to characterize group level differences followed by a logistic regression with variables at the individual and household levels and one "BRAC membership status" variable to account for eligibility, savings and credit	South Asia				model with unspecified skilled training offered to some clients	1. No MF (used as the controls in this review) 2. Skilled training and MF	verbal abuse between herself the client and her husband in the preceding 4 months	
Bajracharya & Amin, 2013[28]	Cross sectional survey – used propensity score matching to address selection bias	Rural and urban, Bangladesh, South Asia	4195 women	2007 Demographic and Health Survey	Any MFI - Grameen, BRAC, ASA, Prosika	Credit and Savings in group lending Model	No MF	Married women reporting any form of violence by her partner in preceding 12 months	NOS 8/11
Dalal et al, 2013[29]	Cross sectional Survey – used chi-squared test to examine difference in IPV exposure and microfinance and demographic variables (age, residence, education, religion and wealth index)	Rural and urban, Bangladesh, South Asia	4465 women	2007 Demographic and Health Survey	Any MFI - Grameen, BRAC, ASA, Proshika	Credit and Savings in group lending Model	No MF	All women reporting any form of violence by her partner in preceding 12 months	NOS 8/11
Murshid et al. 2016[30]	Cross sectional Survey data was used to investigate association between microfinance and domestic violence with predictor variables including economic status, decision making power and demographic variables	Rural and urban, Bangladesh, South Asia	4163 women	2007 Demographic and Health Survey	Any MFI - Grameen, BRAC, ASA, Proshika	Credit and Savings in group lending Model	No MF	Conflicts Tactics Scale based on the battery of questions that asked respondents whether they experienced a number of violent acts that constituted physical and sexual violence	NOS 8/11
Pronyk et al, 2006[31]	Cluster RCT: per-protocol analysis. As only 8 villages were randomised, baseline imbalances were adjusted prior to analysis	Rural, South Africa, Sub-Saharan Africa	538 women (290 intervention, 248 control)	2001, 2005	Local NGO	Credit and savings in group lending model with additional life skills training	No MF	All women reporting intimate partner violence in preceding 12 months	Cochrane Risk-of-Bias – High
Schuler et al, 1996[32]	Cross sectional survey. Conducted multivariate analysis using a logistic regression model with independent variables age, education, religion, whether respondent had any surviving sons or	Rural, Bangladesh, South Asia	1225 women	1992	MFI - Grameen and BRAC	Credit and savings in group lending model	No MF	Women reporting physical beating by husband in the preceding 12 months	NOS 7/11

For peer review only - http://bmjopen.bmj.com/site/about/guidelines.xhtml

	daughters, geographic region, economic level of household, respondent's contribution to family support and, exposure to credit programs.								
Angelucci et al, 2015[33]	Cluster RCT: intent-to-treat analysis on all respondents.	Rural, Mexico, Latin and Central America	1823 women	2009-12	MFI – Compartamos Banco	Credit and savings in group lending model	No MF	Decision-making ability: participation in financial decisions and household issues by non-single women aged 18-60 who are not the only adult in their household.	Cochrane Risk-of-Bias – High
Banerjee et al, 2015[10]	Cluster RCT: intent-to-treat analysis: constructed an equally weighted average z-score of 16 social outcomes to detect any difference.	Urban, India, South Asia	6862 women at first follow-up; 6142 women at second follow-up	2005, 2010	MFI – Spandana	Credit and savings in group lending model	No MF	Index of empowerment encompassing scores across 16 domains, covering decision making, levels of health and education expenditure and school enrollment	Cochrane Risk-of-Bias – High
Beaman et al, 2014[34]	Cluster RCT – intention to treat analysis. The econometric baseline characteristics and variable used in the randomisation process such as household and village characteristics.	Rural, Mali, Sub-Saharan Africa	5425 women	2009, 2012	SHG with NGO support	Credit and savings in self-help group model	No MF	Decision making ability: women's freedom to decide about food and educational expenses and take decisions about business. Index of intra-household decision making power combining individual measures	Cochrane Risk-of-Bias – High
Karlan 2017[35]	Cluster RCT<br>- A polled model controlling for baseline values and district was estimated by an "intention to treat" method.	Rural: Ghana, Malawi, and Uganda	15,000 households	Baseline 2008 to survey at endline in 2011	Cooperative for Assistance and Relief Everywhere (CARE)	Village savings and loan associations	No MF	Decision making ability: women's empowerment index capturing self-reported influence on household decisions, particularly in relation to food expenses for the household, education and health care expenses for the	Cochrane Risk-of-Bias – High

For peer review only - http://bmjopen.bmj.com/site/about/guidelines.xhtml

								children, business expenses if the household operates a business and the women's ability to visit friends	
Mohindra et al, 2008[36]	Cross sectional survey. A three step model including only SHG participation, socioeconomic characteristics and caste was examined with a goodness-of-fit test and odds ratios.	Rural, India, South Asia	928 women	2003	SHG with NGO support	Credit and savings in self-help group model	No MF	Decision-making ability – whether women aged 18-59 reported at least 1 situation (of 5 asked) in which her husband or a male relative was the sole decision-maker	NOS 7/11
Montgomery & Weiss, 2011[37]	Cross sectional survey: analysis accounted for income variables, consumption-expenditure variables, and household characteristics and explored differential effects on urban and rural households	Rural and urban, Pakistan, South Asia	2876 women	2005	Commercial MFI - Khushali	Credit and savings in group lending model	No MF	Decision making ability – women between 15-40 asked whether their opinion is taken into account in a series of household decisions	NOS 7/11
Pitt et al, 2003[9]	Quasi-experimental study using econometric methods similar to Pitt and Kandker et al.[5]	Rural, Bangladesh, South Asia	2074 women	1991/1992, 1998/1999	MFI – Grameen, BRAC, BRDC, ASA	Credit and savings in group lending model	No MF	Empowerment score combining empowerment indicators across several domains of decision making, discussion, finance and mobility	NOS 7/11
Rahman et al, 2009[38]	Quasi-experimental cross-sectional survey. Considered age, education level, spouse's age and education level, household income, asset accumulation and locality in the analysis.	Rural and urban, Bangladesh, South Asia	571 recruited and analysed	Not indicated	MFI - Grameen and BRAC	Credit and Savings in group lending model	No MF	Mobility index; empowerment index	NOS 6/11
Sharif, 2004[39]	Cross sectional survey data were used for econometric analysis with a range of socioeconomic and demographic variables.	Not reported, Bangladesh, South Asia	483 women	1997	MFI - ASA	Credit and savings in group lending model	No MF	Decision making ability – Likert-type responses on women's extent of decision making	NOS 7/11

For peer review only

For peer review only - http://bmjopen.bmj.com/site/about/guidelines.xhtml

								across 6 domains	
Swain & Wallentin, 2009[40]	Quasi-experimental cross-sectional survey. Used the robust maximum likelihood method.	Rural and urban, India, South Asia	961 women	2000 and 2003	SHG with MFI linkage	Savings at group level and credit from MFI in group lending model	No MF	Empowerment score	NOS 5/11
Tarozzi et al, 2015[41]	Cluster RCT Panel of villages data for used for an intent-to-treat analysis to identify the impact of giving access to microcredit rather than actual borrowing	Rural, Ethiopia, Sub-Saharan Africa	6412 households at baseline; 6263 households at follow-up	2003 and 2006	CBOs supported by international NGO	Credit and savings in group lending model	No MF	Decision making ability – fraction of decisions across 20 domains women aged 15-49 were involved in making	Cochrane Risk-of-Bias -High
Zaman, 1999[42]	Cross sectional survey data were used in a multivariate analysis with considerations for the number of eligible households in the village, membership length, and socio-economic differences.	Rural, Bangladesh, South Asia	1568 women	1995	MFI - BRAC	Credit and savings in group lending model	No MF	Decision making ability	NOS 2/11
**Studies with outcome measures of children's nutrition**									
Abubakari et al, 2014[43]	Cross sectional survey – analysis accounted for food acquisition behaviours and demographic characteristics of the households	Rural, Ghana, Sub-Saharan Africa	180 children	2011	Village Savings and Loans Association	Credit and savings in self-help group model	No MF	Anthropometric measurement of nutritional status in children <5 years based on HAZ scores: >-2 well nourished; <-2 to -3 moderate malnutrition; <-3 severe malnutrition	NOS 4/10
Doocy et al, 2005[8]	Cross sectional survey with community controls who were matched by sex and selected by proximity of residence via systematic random sampling.	Rural and urban, Ethiopia Sub-Saharan Africa	608 children	2003	NGO - WISDOM	Credit and Savings in group lending model	Two comparison groups: 1. No MF (used as the controls in this review) 2. New clients <1 cycle of MF	Anthropometric measurement of nutritional status in children aged 6-59 months based on MUAC: <11cm severe malnutrition; 11-12.5cm moderate malnutrition	NOS 6/11
Friesen et al, 2012[44]	Cross sectional survey. Analysis included socioeconomic and demographic factors including household and	Rural and urban, Ghana, Sub-Saharan Africa	204 children	June to August 2011	Local microfinance bank (previously with NGO support)	Credit and savings in group lending model	No MF	Anthropometric measurement of nutritional status in children aged 6-23 months based on	NOS 7/11

For peer review only - http://bmjopen.bmj.com/site/about/guidelines.xhtml

	maternal characteristics and child's age and sex.							proportion underweight (WAZ<-2), stunted (LAZ<-2) and wasted (WLZ<-2)	
Marquis et al, 2015[7]	Quasi-experimental design with longitudinal follow-up. Bivariate analysis between anthropometric measures and explanatory variables and sensitivity analysis was performed to examine within subject variations	Rural, Ghana, Sub-Saharan Africa	608 caregivers with children	Approximately 4-monthly between April 2006 and Dec 2007	Credit and savings association	Credit and savings in self-help group model with additional health, nutrition and entrepreneur education	No MF	Anthropometric measurement of nutritional status in children aged 2-5 years based on WAZ, HAZ and BAZ scores	Cochrane Risk-of-Bias – High
Ojha et al, 2017[45]	Cluster randomized controlled trial with cross sectional follow up and intention to treat analysis	Rural, India, South Asia	1377 children	August 2013 to March 2016	Rojiroti microfinance programme	Savings and credit in peer led self-help groups	No MF	Anthropometric measures of children 0-5 years of age WHZ, HAZ, WAZ, MUAC	Cochrane Risk of Bias - High

*MF – Microfinance , FP- Family Planning, SHG – self-help group

For peer review only - http://bmjopen.bmj.com/site/about/guidelines.xhtml

Nature of the microfinance interventions evaluated: The most common microfinance model was group-lending as provided by formal microfinance institutions (MFIs)[9 10 24-29 32 33 38 39 41 44 45] and community-based organisations (CBOs)[7 8 23 31 35 41]. MFIs required clients to be women above the age of eighteen, own less than 0·5 decimals of land (435 square feet) and have at least one household member in casual employment. Self-help groups and CBOs had fewer eligibility criteria but with greater emphasis on accumulation of savings [7 26 30 34 36 40 43 45]. In some studies microfinance was coupled with additional social and health interventions[7 23 27 31].

Findings of Studies by Outcome

Contraceptive Use

Four studies[5 23 25 26] evaluated the impact of microfinance on self-reported use of contraception using data from household cross-sectional surveys. One study[23] evaluated an intervention that combined microfinance with family planning education in Ethiopia. The other 3 studies[24-26] recruited clients from non-commercial MFIs in Bangladesh.

The impact of microfinance in the Ethiopian study was estimated at the level of the *kebele* (a cluster of villages) and showed no significant change in the proportion of married women reporting contraceptive use; individual-level estimates of the impact of microfinance were not available. A fixed-effects meta-analysis of individual-level data from the three Bangladeshi studies showed that women participating in microfinance were 64% more likely to report contraceptive use than non-clients [OR=1.64, 95%CI 1.45 1.86; Figure 2]. There was no heterogeneity between the studies, which is plausible given the similarity in the average age and socio-economic status of participants.

Female empowerment

Seventeen studies evaluated the impact of microfinance on female empowerment. Eight were conventional cross-sectional studies[27-30 32 36 37 39 42], 3 were quasi-experimental[9 38 40] and 6 were cluster-randomised controlled trials (cluster RCTs)[10 31 33 34 41]. Twelve studies were from South Asia, 3 from SSA and 1 from LAC. These studies included evaluated different methods of empowerment.

Intimate partner violence (IPV): Five cross-sectional surveys[27-30 32] and 1 cluster RCT[31] reported this outcome. One survey[28] showed a significant 24% (95%CI 1·05-1·44) increase in odds of IPV among microfinance clients compared to non-clients. On the other hand, the cluster RCT[31] demonstrated a significant decrease in IPV (adjusted risk ratio 0.45, 95%CI 0.23-0.91) and another survey[32] similarly

For peer review only - http://bmjopen.bmj.com/site/about/guidelines.xhtml

showed reductions among clients of the two MFIs studied (OR=0.44, 95%CI 0.28-0.70 and OR=0.30, 95%CI 0.18-0.51). Dalal *et al*[29] found that microfinance clients with secondary and higher education were 2-3 times more likely to experience IPV than comparable non-clients (*p*=<0·001), while wealthier clients were twice as likely to experience IPV than comparable non-clients (*p*=<0·001); there were no changes in exposure to IPV amongst the least educated and poorest groups. This finding was confirmed by Murshid et al.[30] who also analysed the data from the same Bangladeshi Demographic Health Survey of 2007.

A meta-analysis was not conducted due to high heterogeneity (I^2=91·3%). This heterogeneity could have arisen because the threshold for reporting violence or the framing of the question may have differed between settings. The cluster RCT[31] was different both in design and in the add-on life skills training, which may have introduced further heterogeneity. The association between IPV and microfinance is therefore inconclusive.

Decision making ability: Eight studies were included for this outcome, 5 from South Asia[33 36 37 39 42] and 3 from SSA[34 41], with 4 cluster RCTs[33-35 41], and 4 cross-sectional surveys[36 37 39 42]. This measure analysed a change from not being involved in decision making to being an active participant in household decisions. The outcome measures used were diverse and therefore unsuitable for meta-analysis. The results have been tabulated in more detail in Supplement 4 and include participation in financial and other household decisions (e.g. children's education and healthcare). Just over half the studies[33 37 39 42] showed a slightly higher degree of participation in certain household decisions by microfinance clients compared to non-clients. The other studies did not report any statistically significant changes. The impact of microfinance on women's decision making is therefore inconclusive.

***Freedom to travel (mobility)*:** In the one study that assessed mobility, non-clients were more mobile than clients in one region, but in the two other regions studied the reverse was true[38]. No formal statistical comparisons between groups were presented.

Children's nutrition

Five studies, 4 from SSA[7 8 43 44] and 1 from India[45], evaluated the effect of microfinance on children's nutrition. Three[8 43 44] were cross-sectional surveys, 1 was a quasi-experimental study with a 16 month follow-up period[7] while 1 was a cluster randomised controlled trial[45]. Two studies[7 44] included only children between 6-36 months of age while the other 3 included children under five years.

For peer review only - http://bmjopen.bmj.com/site/about/guidelines.xhtml

Doocy *et al* reported that children of women non-clients were 79% more likely to be wasted than children of clients (OR=1·79 95%CI 0·87-3·79)[8]. However, Friesen et al reported increased wasting among children of clients compared to non-clients (OR=1·15 95%CI 0·30-4·43)[44]. Neither association was statistically significant. As the baseline group used was different and there were no raw data available, it was not possible to recalculate the ORs for pooling by meta-analysis.

One cross-sectional study found that the prevalence of malnutrition, based on HAZ-scores, was lower amongst children of microfinance clients than those of non-clients[43]. A longitudinal study measured HAZ, WAZ and BAZ-scores every four months for 16 months[7]. The authors demonstrated a mean difference in WAZ scores of 0.28 at 8 to 12 months in favour of the intervention group and significant but smaller differences at four months and 16 months. At 16 months, HAZ-scores were significantly higher in the intervention group with a mean difference of 0·19 between the two groups. Meta-analysis was not possible as the studies used different statistical measures to present their results.

Ojha et al. reported that in a cross-sectional survey conducted 18 months after random allocation to received immediate microfinance vs. delayed microfinance (after 18 months), 0-5 year old children in the villages that received immediate microfinance had a significantly better WHZ compared to children in the villages that did not receive microfinance with a mean difference of 0.35 SD[45]. They found similar differences in WAZ, and prevalence of wasting, underweight and moderate and severe malnutrition as measured by mid-upper arm circumferences but there was no difference in HAZ or prevalence of stunting between the two groups.

Publication Bias: A funnel plot found no evidence of publication bias in the studies that reported the impact of microfinance on IPV (Egger's test *p*-value=0·106). The possibility of publication bias could not be assessed for the other outcomes.

DISCUSSION

Summary of evidence: Table 3 summarises the impact of microfinance across the three outcome domains based on the quantitative and qualitative syntheses described above.

Table 3: Summary of Results of the Review

For peer review only - http://bmjopen.bmj.com/site/about/guidelines.xhtml

Outcome	Summary of impact of microfinance
Use of contraception	Women participating in microfinance schemes were significantly more likely to report using contraception.
Female empowerment	
Intimate partner violence	Conflicting results, with some studies reporting increased and others decreased IPV in microfinance participants.
Decision making ability	Most studies showed no effect but a minority showed a significant positive effect on some areas of decision-making.
Mobility	No statistically significant impact.
Overall empowerment score	Positive impact in two studies with mixed results and no change in two others.
Children's nutrition	Positive impact in three of five studies, with no difference found in the remaining studies.

Seventeen of the 27 studies included in the review were from South Asia. This may limit the generalisability of the findings of this review to other geographical regions. However, this was expected as 84% of all microfinance clients are to be found in South Asia[46].

Proposed mechanisms

Microfinance (whilst primarily improving economic stability) might empower women and improve child nutrition though a number of mechanisms. A small source of income, which is available primarily to the woman in the household, could increase the "bargaining power" of female participants, in household decision making. Peer support and shared learning from other participants might have a similar effect. We have chosen the outcome measures most likely to reflect this increased bargaining power, including a woman's decisions about contraception and her self-reported empowerment. Furthermore, that women are often the primary household decision-makers on issues such as buying food (which will affect child nutrition) and on access to healthcare for children. These factors could interact to enable women to overcome social, cultural and economic barriers that affect their status (Figure 3)

Contraceptive Use

Where individual-level data were available, the odds of reporting contraceptive use were higher in women participating in microfinance compared to those who did not. It has been argued that the

For peer review only - http://bmjopen.bmj.com/site/about/guidelines.xhtml

women who self-select to join microfinance groups are more empowered than other women and this may in itself increase their likelihood of using contraception[4]. However, by comparing reported use in this group before and after the intervention[23 25], it is possible to demonstrate a positive effect attributable to microfinance, even with an inherent empowered state.

Markers of female empowerment

Intimate Partner Violence

Gender-related violence is known to be most commonly perpetuated by a person close to the woman, usually an intimate partner[46]. Although a reduction in IPV is one of the expected benefits of empowerment of women through microfinance, empowerment may also enable women to report more IPV, thus increasing the rate of reported IPV. One cluster RCT[31] reported a reduction in IPV among microfinance clients. However, the combined microfinance with life skills training may have resulted in an intervention group different from the standard client therefore limiting the generalisability of their findings. The authors of this study argued that their training empowered the women to reveal IPV, therefore reducing underreporting[31]. Underreporting of IPV is common in many studies due to its sensitive nature[47]. Studies used trained local female interviewers to limit underreporting, but despite this, the response rate to IPV questions in one study was only 41%[29]. Furthermore, women participating in microfinance may want to only highlight positive impacts of the intervention and not reveal any IPV. This raises ethical concerns that studies may fail to detect violence where it is actually present[47].

Studies that have reported increase in IPV linked to microfinance programmes[29] have also argued that microfinance loans may have caused more economic stress in the family leading to greater occasions for conflict. Some authors explain this as the "status inconsistency theory" where in status differentials may lead to dysfunctional behaviour when and individual who expects to have a higher status in a relationship is threatened by the increase in the status of another[30]. Previously there may have been fewer conflicts as the man would have managed finances single-handedly while with empowerment, the wife becomes involved in these decisions, generating more occasions where conflict leading to IPV could occur.

Decision Making Ability

In most cases, the decision-making ability of women participating in microfinance was not significantly different from that of non-clients. However, most studies analysed women's perceived decision-making ability, which may be different to their actual decision-making capability. In addition, composite indices of decision-making ability make it hard to untangle any impact of

For peer review only - http://bmjopen.bmj.com/site/about/guidelines.xhtml

microfinance on decisions which are typically male-dominated (such as child marriage and education) and decisions which are traditionally less so (such as those related to the purchase of food).

Children's nutrition

Three studies[8 44 45] reported a lower likelihood of severe acute malnutrition in children of women participating in microfinance compared to non-participants, including one that showed a statistically significant reduction in malnutrition[45]. Combining microfinance with nutritional education, as was the case in one study[7], showed improvement in nutritional status in children of participating care-givers than non-participating care-givers. However, it is then difficult to isolate the specific effect of microfinance. In one SHG study[43] no attempt was made to adjust for other variables, such as household resources or education status, which may be a source of confounding.

Additionally, the inclusion of HAZ scores as a measure of nutritional status[43 44] in a cross-sectional study may be misleading. In their cluster randomised trial, Ojha et al. report an improvement in all other indices of malnutrition other than HAZ and stunting after an 18 month period[45]. Height-for-age measures the effect of poor nutrition on the growth of a child. Growth faltering is slow in reversal and requires a longer follow-up period to detect[48]. It may be more prudent to use acute measures of malnutrition such as wasting (WHZ) which are likely to be more sensitive to change in nutritional status over shorter periods.

Strengths and limitations

Five comprehensive databases were searched in this review, including a large economic database. The use of multiple indicators to measure women's empowerment and children's nutrition also served to broaden the search to reduce the likelihood of missing relevant articles. The selection was carried out independently by two authors without any language restrictions, particularly important given the geographical regions studied.

The models used to deliver microfinance services varied across included studies. Some combined microfinance with education on family planning[23], life skills[31] or health, nutrition and entrepreneurial skills[7], which makes it difficult to evaluate the effect of microfinance alone. Although all interventions were taken to be similar for the purposes of this review, it is possible that the way the microfinance services were provided may have influenced the outcome. Given the small number

For peer review only - http://bmjopen.bmj.com/site/about/guidelines.xhtml

of interventions of each type reviewed here, it is not possible to suggest a model of microfinance that is superior to others in terms of social performance.

In general, the most common source of bias in studies of the social impact of microfinance is selection bias, as participants *self-select* to either participate or not participate in the programme. Although, it may be argued that it would be difficult to randomise people to microfinance as the intervention may not be desired by all; therefore measuring effectiveness in those who did not desire it to begin with, may be problematic. Whilst a cluster RCT might guard against selection bias, a recent study[10] highlighted the current challenge in achieving randomisation due to the widespread diffusion of microfinance in some regions of South Asia leading to difficulties in identifying unexposed control clusters. Therefore, we included non-randomised studies in this review in order to not limit the evidence considered. The non-randomised studies included dealt with self-selection bias in two main ways, using either panel data in a quasi-experimental design or propensity score matching (PSM). However, additional analysis in of one of the studies included in this review suggested that the reduction in intimate partner violence demonstrated using conventional statistical methods did not hold when PSM was used[28].

The average follow-up period of the studies included was three years. An alternative explanation for their statistically non-significant findings is that the observation period may have not been long enough to detect any change or may have missed any fleeting changes that occurred before the follow up survey. While changes in some measures of children's malnutrition may be detectable within three years, changes in other outcomes requiring a shift in cultural and social norms may take much longer

Conclusions

In conclusion, our findings suggest that for the types of microfinance interventions assessed in this study, there may be an association between microfinance and increasing contraceptive use, improving female empowerment and better children's nutrition. However, as only 6 of 27 studies included in this review were randomised trials any conclusions about direct causation must be guarded. However, the wide diversity in reported outcomes, study design, statistical methods and microfinance models makes it difficult to synthesise evaluation data statistically. Thus further studies are required to evaluate the social performance of microfinance. The design of future studies requires effective and clearly described randomisation, harmonisation of appropriate outcome

For peer review only - http://bmjopen.bmj.com/site/about/guidelines.xhtml

measures and avoidance of confounders. Incorporating evaluation methods at the onset of a microfinance programme could help address many of the weaknesses identified here. While this may not be practical in areas where microfinance is fully established, areas with an increasing number of microfinance programmes, for example sub-Saharan Africa, would benefit.

ACKNOWLEDGEMENTS

We thank Magdalena Opazo Breton and Gabriella Zapata for their assistance in translating manuscripts written in Spanish and Portuguese.

We thank Dr Rajeev Kamal and his team at the A N Sinha Institute of Social Sciences, Patna, India for organising the public involvement workshop.

CONTRIBUTION STATEMENT

WG, LS, SO and AS conceived and designed the study. WG and LS independently carried out the title, abstract and full text screening and quality assessment. WG conducted the meta-analyses and wrote the first draft of the paper. SS updated the search in 2018 and completed the updated title and abstract screening. The updated full text screening was performed by SO, SS and WG. All authors critically revised subsequent drafts, and have approved the final version.

For peer review only

REFERENCES

1. Ledgerwood J, Earne J, Nelson C. *The New Microfinance Handbook: A Financial Market System*: The International Bank for Reconstruction and Development/The World Bank, 2013.
2. Robinson MS, Marguerite S, Fidler, et al. The microfinance revolution : sustainable finance for the poor 2001; 1(Report number: 23250). http://documents.worldbank.org/curated/en/226941468049448875/sustainable-finance-for-the-poor.
3. Schurmann AT, Johnston HB. The group-lending model and social closure: microcredit, exclusion, and health in Bangladesh. J Health Popul Nutr 2009;**27**(4):518-27.
4. Goldberg N. Measuring the Impact of Microfinance: Taking Stock of What We Know. Washinton DC, 2005.
5. Pitt MM, Khandker SR. The impact of group-based credit programs on poor households in Bangladesh: Does the gender of participants matter? J Polit Econ 1998;**106**(5):958-96.
6. MicroRate. The State of Microfinance Investment 2013. Secondary The State of Microfinance Investment 2013 2013. http://www.microrate.com/media/downloads/2013/11/MicroRate-The-State-of-Microfinance-Investment-2013.pdf.
7. Marquis GS, Colecraft EK, Sakyi-Dawson O, et al. An Integrated Microcredit, Entrepreneurial Training, and Nutrition Education Intervention Is Associated with Better Growth Among Preschool-Aged Children in Rural Ghana. J Nutr 2015;**145**(2):335-43.
8. Doocy S, Teferra S, Norell D, et al. Credit program outcomes: coping capacity and nutritional status in the food insecure context of Ethiopia. Soc Sci Med 2005;**60**(10):2371-82.
9. Pitt MM, Khandker SR, Shahidur R, et al. Does Micro-Credit Empower Women : Evidence from Bangladesh. Policy Research Working Paper;No. 2998. . 2003.
10. Banerjee A, Duflo E, Glennerster R, et al. The miracle of microfinance? Evidence from a randomized evaluation. American Economic Journal: Applied Economics 2015;**7**(1):22-53.
11. Bank W. World Bank: countries. Secondary World Bank: countries 2016. http://www.worldbank.org/en/country.
12. Bank W. Poverty Overview. Secondary Poverty Overview 2018. http://worldbank.org/en/topic/poverty/overview.
13. Odell K. Measuring the Impact of Microfinance: Taking another look. 2010. https://grameenfoundation.org/blog/measuring-impact-microfinance-taking-another-look#.W1iROdVKiUk.
14. WHO. Monitoring Maternal, Newborn and Child Health: Understanding Key Progress Indicators. 2011.
15. Murray CJL, Salomon JA, Mathers C, et al. Summary Measures of Population Health: Concepts, Ethics, Measurement and Applications. 2002. http://apps.who.int/iris/bitstream/handle/10665/42439/9241545518.pdf?sequence=1&isAllowed=y.
16. Project UM. Investing in Development: A Practical Plan to Achieve the Millennium Development Goals. . 2005.
17. Rustagi P. Situation of Women in South Asia: Some Dimensions. . 2015.
18. ECMIA. Violence and Indigenous Women. 2013. http://www.ncdsv.org/images/Ecmia-Chirapaq_Violence-and-Indigenous-Women_3-2013.pdf.
19. Kabeer N. Resources, agency, achievements: Reflections on the measurement of women's empowerment. Dev Change 1999;**30**(3):435-64.
20. CDC. Intimate Partner Violence. Secondary Intimate Partner Violence 2018. https://www.cdc.gov/violenceprevention/intimatepartnerviolence/.
21. *Cochrane Handbook for Systematic Reviews of Interventions*: The Cochrane Collaboration, 2011.

For peer review only - http://bmjopen.bmj.com/site/about/guidelines.xhtml

22. Wells GA, Shea B, O'Connell, et al. The Newcastle-Ottawa Scale (NOS) for assessing the quality of nonrandomised studies in meta-analyses. Secondary The Newcastle-Ottawa Scale (NOS) for assessing the quality of nonrandomised studies in meta-analyses. http://www.ohri.ca/programs/clinical_epidemiology/oxford.asp.
23. Desai J, Tarozzi A. Microcredit, Family Planning Programs, and Contraceptive Behavior: Evidence From a Field Experiment in Ethiopia. Demography 2011;**48**(2):749-82.
24. Pitt MM, Khandker SR. Household and Intrahousehold Impact of the Grameen Bank and Similar Targeted Credit Programs in Bangladesh. 1996. http://documents.worldbank.org/curated/en/385431468740690795/pdf/multi-page.pdf.
25. Steele F, Amin S, Naved RT. Savings/credit group formation and change in contraception. Demography 2001;**38**(2):267-82.
26. Murshid NS, Ely GE. Microfinance participation and contraceptive use: does control over resources matter? J Public Health (Oxf) 2017;**39**(2):e27-e32.
27. Ahmed SM. Intimate partner violence against women: Experiences from a woman-focused development programme in Matlab, Bangladesh. Journal of Health Population and Nutrition 2005;**23**(1):95-101.
28. Bajracharya A, Amin S. Microcredit and Domestic Violence in Bangladesh: An Exploration of Selection Bias Influences. Demography 2013;**50**(5):1819-43.
29. Dalal K, Dahlstrom O, Timpka T. Interactions between microfinance programmes and non-economic empowerment of women associated with intimate partner violence in Bangladesh: a cross-sectional study. Bmj Open 2013;**3**(12).
30. Murshid NS, Akincigil A, Zippay A. Microfinance Participation and Domestic Violence in Bangladesh: Results From a Nationally Representative Survey. J Interpers Violence 2016;**31**(9):1579-96.
31. Pronyk PM, Hargreaves JR, Kim JC, et al. Effect of a structural intervention for the prevention of intimate-partner violence and HIV in rural South Africa: a cluster randomised trial. Lancet 2006;**368**(9551):1973-83.
32. Schuler SR, Hashemi SM, Riley AP, et al. Credit programs, patriarchy and men's violence against women in rural Bangladesh. Soc Sci Med 1996;**43**(12):1729-42.
33. Angelucci M, Karlan D, Zinman J. Microcredit Impacts: Evidence from a Randomized Microcredit Program Placement Experiment by Compartamos Banco. Am Econ J-Appl Econ 2015;**7**(1):151-82.
34. Beaman L, Karlan D, Thuysbaert B. Saving for a (not So) Rainy Day: A Randomized Evaluation of Savings Groups in Mali. 2014. http://www.nber.org/papers/w20600.pdf.
35. Karlan D, Savonitto B, Thuysbaert B, et al. Impact of savings groups on the lives of the poor. P Natl Acad Sci USA 2017;**114**(12):3079-84.
36. Mohindra KS, Haddad S, Narayana D. Can microcredit help improve the health of poor women? Some findings from a cross-sectional study in Kerala, India. Int J Equity Health 2008;**7**.
37. Montgomery H, Weiss J. Can Commercially-oriented Microfinance Help Meet the Millennium Development Goals? Evidence from Pakistan. World Dev 2011;**39**(1):87-109.
38. Rahman S, Junankar PN, Mallik G. Factors influencing women's empowerment on microcredit borrowers: a case study in Bangladesh. J Asia Pac Econ 2009;**14**(3):287-303.
39. Sharif NR. Microcredit programs and women's decision-making status: Further evidence from Bangladesh. Can J Dev Stud 2004;**25**(3):465-80.
40. Swain RB, Wallentin FY. Does microfinance empower women? Evidence from self-help groups in India. International Review of Applied Economics 2009;**23**(5):541-56.
41. Tarozzi A, Desai J, Johnson K. The Impacts of Microcredit: Evidence from Ethiopia. Am Econ J-Appl Econ 2015;**7**(1):54-89.
42. Zaman H. Assessing the Impact of Micro-Credit on Poverty and Vulnerability in Bangladesh. Policy,. 1999.

For peer review only - http://bmjopen.bmj.com/site/about/guidelines.xhtml

http://documents.worldbank.org/curated/en/630461468741915080/Assessing-the-impact-of-micro-credit-on-poverty-and-vulnerability-in-Bangladesh.

43. Abubakari A, Sadik Bb, Keisan Y. Impact of Village Savings and Loans Associations on the Nutritional Status of Under-Five Children: A Case Study in the Sissala West District of Upper West Region. Pakistan Journal of Nutrition 2014;**13**(7):309-96.

44. Friesen V, Marquis GS, Fallon K, et al. Tackling poverty through private sector microcredit programs in Ghana: does infant and young child nutrition improve? McGill University, 2012.

45. Ojha S, Szatkowski L, Sinha R, et al. PO5 Effects of the rojiroti microfinance programme on nutrition in very poor children under five in india. Archives of Disease in Childhood 2017;**102**(A3).

46. Devries KM, Mak JYT, Garcia-Moreno C, et al. The Global Prevalence of Intimate Partner Violence Against Women. Science 2013;**340**(6140):1527-28.

47. WHO. Ethical and Safety Recommendations for Research on Domestic Violence Against Women. 2001. http://www.who.int/gender/violence/womenfirtseng.pdf.

48. Shrimpton R, Victora CG, de Onis M, et al. Worldwide timing of growth faltering: implications for nutritional interventions. Pediatrics 2001;**107**(5):E75.

For peer review only

For peer review only - http://bmjopen.bmj.com/site/about/guidelines.xhtml

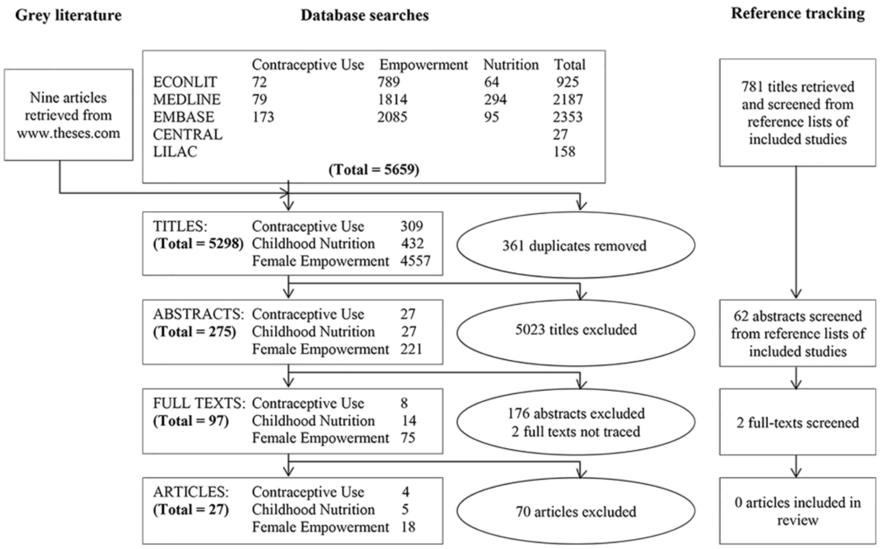

Figure 1. PRISMA flow chart

287x179mm (300 x 300 DPI)

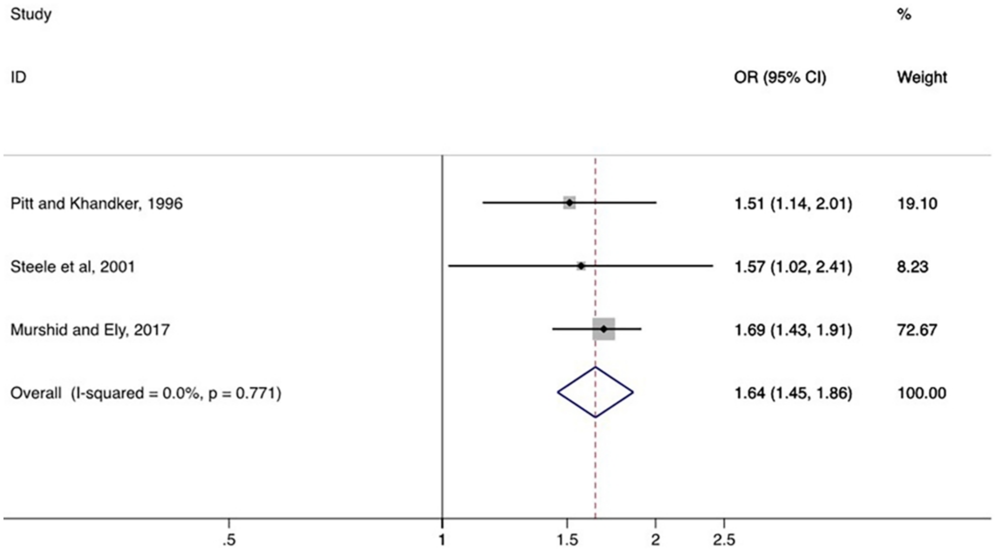

Figure 2.Fixed effects meta-analysis of effect of microfinance participation on women reporting contraceptive use

287x179mm (300 x 300 DPI)

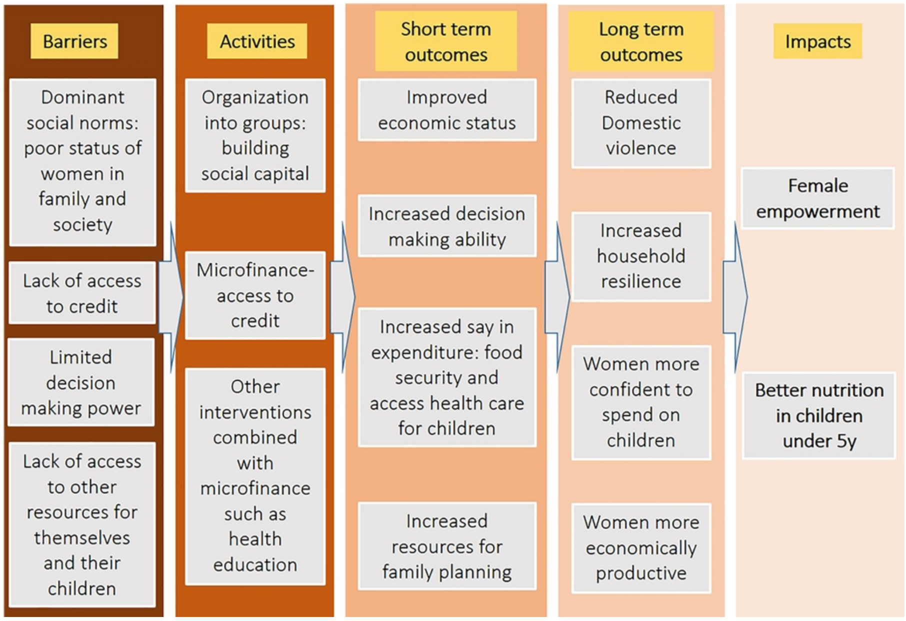

Figure 3. Theory of change model linking microfinance to women's wellbeing and children's nutrition

287x179mm (300 x 300 DPI)

Is microfinance associated with changes in women's wellbeing and childhood nutrition? A systematic review and meta-analysis

Wanjiku Gichuru[1] MPH (International Health), Shalini Ojha[2] PhD, Alan R Smyth[3] MD, Lisa Szatkowski PhD[1]

[1]University of Nottingham, Division of Epidemiology and Public Health, Clinical Sciences Building, Nottingham City Hospital, Nottingham, UK, NG5 1PB

[2]University of Nottingham, Division of Graduate Entry Medicine, Derby Medical School, Derby, DE22 3DT

[3]Child Health, Obstetrics and Gynaecology, E Floor, East Block, Queen's Medical Centre, Nottingham, UK, NG7 2UH

Corresponding author: shalini.ojha@nottingham.ac.uk

For peer review only

For peer review only - http://bmjopen.bmj.com/site/about/guidelines.xhtml

Supplementary material

Supplement 1: Search Terms

1. MEDLINE SEARCH STRATEGY

Contraceptive Use

1. (microfinanc* or microcredit or microloan or "micro-financ*" or "micro-credit" or "micro-loan" or "micro financ*" or "micro credit" or "micro loan" or "small loan" or "small lend" or "micro enterpris*" or "micro enterpreneur").mp. [mp=title, abstract, original title, name of substance word, subject heading word, keyword heading word, protocol supplementary concept word, rare disease supplementary concept word, unique identifier]
2. (random* or "randomi*ed control trial" or "randomi*ed cluster trial" or study or analys* or cohort or "cross section*" or "cross-section*" or survey or " pre test and post test" or "pre-test and post-test" or "before and after" or "interrupted time series" or "time series" or "time-series" or "time points").mp. [mp=title, abstract, original title, name of substance word, subject heading word, keyword heading word, protocol supplementary concept word, rare disease supplementary concept word, unique identifier]
3. ("contraceptive*" or "contraception" or "reproductive" or gynaecolog* or gynecolog* or "birth control" or fertility).mp. [mp=title, abstract, original title, name of substance word, subject heading word, keyword heading word, protocol supplementary concept word, rare disease supplementary concept word, unique identifier]
4. exp contraceptive behavior/
5. 3 or 4
6. economics/ or financial support/
7. 1 or 6
8. 2 and 5 and 7
9. limit 8 to (humans and yr="1990 -Current")

Female Empowerment

1. economics/ or financial support/
2. (microfinanc* or microcredit or microloan or "micro-financ*" or "micro-credit" or "micro-loan" or "micro financ*" or "micro credit" or "micro loan" or "small loan" or "small lend" or "micro enterpris*" or "micro enterpreneur").mp. [mp=title, abstract, original title, name of substance word,

For peer review only - http://bmjopen.bmj.com/site/about/guidelines.xhtml

subject heading word, keyword heading word, protocol supplementary concept word, rare disease supplementary concept word, unique identifier]

3. ("health" or "outcome" or "evaluat*" or "intervention" or "impact" or "result*" or "effect*").mp. [mp=title, abstract, original title, name of substance word, subject heading word, keyword heading word, protocol supplementary concept word, rare disease supplementary concept word, unique identifier]

4. (random* or "randomi*ed control trial" or "randomi*ed cluster trial" or study or analys* or cohort or "cross section*" or "cross-section*" or survey or " pre test and post test" or "pre-test and post-test" or "before and after" or "interrupted time series" or "time series" or "time-series" or "time points").mp. [mp=title, abstract, original title, name of substance word, subject heading word, keyword heading word, protocol supplementary concept word, rare disease supplementary concept word, unique identifier]

5. ("gender" or "female" or "gender violence" or "assault" or "women" or "woman" or "women's empowerment" or "empowerment" or " women's rights" or "gender equality" or "intimate partner violence" or " travel* without permission" or "girl* education" or "school enrollment" or "school enrolment" or "infanticide").mp. [mp=title, abstract, original title, name of substance word, subject heading word, keyword heading word, protocol supplementary concept word, rare disease supplementary concept word, unique identifier]

6. 1 or 2

7. 3 or 5

8. 4 and 6 and 7

9. limit 8 to (humans and yr="1990 -Current")

Nutrition

1. (microfinanc* or microcredit or microloan or "micro-financ*" or "micro-credit" or "micro-loan" or "micro financ*" or "micro credit" or "micro loan" or "small loan" or "small lend" or "micro enterpris*" or "micro enterpreneur").mp. [mp=title, abstract, original title, name of substance word, subject heading word, keyword heading word, protocol supplementary concept word, rare disease supplementary concept word, unique identifier]

2. economics/ or exp financial support/

3. Child Nutrition Disorders/ or Nutrition Disorders/ or Nutrition Surveys/

4. (nutrition or malnutrition or undernutrition or under-nutrition or "MUAC" or "mid-upper arm circumference" or "Z score*" or Z-scores or "weight-for-age" or stunting or "weight-for-height" or "weight for age" or "weight for height" or underweight or “height for age” or “height-for-age” or

For peer review only - http://bmjopen.bmj.com/site/about/guidelines.xhtml

wasting or “whz” or wasting).mp. [mp=title, abstract, original title, name of substance word, subject heading word, keyword heading word, protocol supplementary concept word, rare disease supplementary concept word, unique identifier]

5. (random* or "randomi*ed control trial" or "randomi*ed cluster trial" or study or analys* or cohort or "cross section*" or "cross-section*" or survey or "pre test and post test" or "pre-test and post-test" or "before and after" or "interrupted time series" or "time series" or "time-series" or "time points").mp. [mp=title, abstract, original title, name of substance word, subject heading word, keyword heading word, protocol supplementary concept word, rare disease supplementary concept word, unique identifier]

6. 1 or 2

7. 3 or 4

8. 6 and 7

9. 5 and 8

10. limit 9 to (yr="1990 -Current")

II EMBASE SEARCH STRATEGY

Contraceptive Use

1. (microfinanc* or microcredit or microloan or "micro-financ*" or "micro-credit" or "micro-loan" or "micro financ*" or "micro credit" or "micro loan" or "small loan" or "small lend" or "micro enterpris*" or "micro enterpreneur").mp. [mp=title, abstract, heading word, drug trade name, original title, device manufacturer, drug manufacturer, device trade name, keyword]

2. (random* or "randomi*ed control trial" or "randomi*ed cluster trial" or study or analys* or cohort or "cross section*" or "cross-section*" or survey or "pre test and post test" or "pre-test and post-test" or "before and after" or "interrupted time series" or "time series" or "time-series" or "time points").mp. [mp=title, abstract, heading word, drug trade name, original title, device manufacturer, drug manufacturer, device trade name, keyword]

3. exp finance/

4. 1 or 3

5. ("contraceptive*" or "contraception" or "reproductive" or gynaecolog* or gynecolog* or "birth control" or fertility).mp. [mp=title, abstract, heading word, drug trade name, original title, device manufacturer, drug manufacturer, device trade name, keyword]

6. exp contraceptive behavior/

7. 5 or 6

For peer review only - http://bmjopen.bmj.com/site/about/guidelines.xhtml

For peer review only

8. 4 and 7

9. 2 and 8

10. limit 9 to (human and yr="1990 -Current")

Female Empowerment

1. (microfinanc* or microcredit or microloan or "micro-financ*" or "micro-credit" or "micro-loan" or "micro financ*" or "micro credit" or "micro loan" or "small loan" or "small lend" or "micro enterpris*" or "micro enterpreneur").mp. [mp=title, abstract, heading word, drug trade name, original title, device manufacturer, drug manufacturer, device trade name, keyword]

2. ("health" or "outcome" or "evaluat*" or "intervention" or "impact" or "result*" or "effect*").mp. [mp=title, abstract, heading word, drug trade name, original title, device manufacturer, drug manufacturer, device trade name, keyword]

3. (random* or "randomi*ed control trial" or "randomi*ed cluster trial" or study or analys* or cohort or "cross section*" or "cross-section*" or survey or "pre test and post test" or "pre-test and post-test" or "before and after" or "interrupted time series" or "time series" or "time-series" or "time points").mp. [mp=title, abstract, heading word, drug trade name, original title, device manufacturer, drug manufacturer, device trade name, keyword]

4. ("gender" or "female" or "gender violence" or "assault" or "women" or "woman" or "women's empowerment" or "empowerment" or " women's rights" or "gender equality" or "intimate partner violence" or " travel* without permission" or "girl* education" or "school enrollment" or "school enrolment" or "infanticide").mp. [mp=title, abstract, heading word, drug trade name, original title, device manufacturer, drug manufacturer, device trade name, keyword]

5. exp finance/

6. 1 or 5

7. 2 or 4

8. 3 and 6 and 7

9. limit 8 to (human and yr="1990 -Current")

10. 1 and 3 and 7

11. limit 10 to (human and yr="1990 -Current")

Nutrition

1. (microfinanc* or microcredit or microloan or "micro-financ*" or "micro-credit" or "micro-loan" or "micro financ*" or "micro credit" or "micro loan" or "small loan" or "small lend" or "micro

For peer review only - http://bmjopen.bmj.com/site/about/guidelines.xhtml

enterpris*" or "micro enterpreneur").mp. [mp=title, abstract, heading word, drug trade name, original title, device manufacturer, drug manufacturer, device trade name, keyword]

2. Child Nutrition Disorders/ or Nutrition Disorders/ or Nutrition Surveys/

3. (nutrition or malnutrition or undernutrition or under-nutrition or "MUAC" or "mid-upper arm circumference" or "Z score*" or Z-scores or "weight-for-age" or stunting or "weight-for-height" or "weight for age" or "weight for height" or underweight or “height for age” or “height-for-age” or wasting or “whz” or wasting).mp. [mp=title, abstract, heading word, drug trade name, original title, device manufacturer, drug manufacturer, device trade name, keyword]

4. (random* or "randomi*ed control trial" or "randomi*ed cluster trial" or study or analys* or cohort or "cross section*" or "cross-section*" or survey or "pre test and post test" or "pre-test and post-test" or "before and after" or "interrupted time series" or "time series" or "time-series" or "time points").mp. [mp=title, abstract, heading word, drug trade name, original title, device manufacturer, drug manufacturer, device trade name, keyword]

5. 2 or 3

6. exp finance/

7. 1 or 6

8. 5 and 7

9. 4 and 8

10. limit 9 to (human and yr="1990 -Current")

III ECONLIT SEARCH STRATEGY

Female Empowerment

microfinanc* or microcredit or microloan or "micro-financ*" or "micro-credit" or "micro-loan" or "micro financ*" or "micro credit" or "micro loan" or "small loan" or "small lend" or "micro enterpris*" or "micro entrepreneur”

AND

"health" or "outcome" or "evaluat*" or "intervention" or "impact" or "result*" or "effect*" or "gender" or "female" or "gender violence" or "assault" or "women" or "woman" or "women's empowerment" or "empowerment" or " women's rights" or "gender equality" or "intimate partner violence" or " travel* without permission" or "girl* education" or "school enrollment" or "school enrolment" or "infanticide"

AND

For peer review only - http://bmjopen.bmj.com/site/about/guidelines.xhtml

random* or "randomi*ed control trial" or "randomi*ed cluster trial" or study or analys* or cohort or "cross section*" or "cross-section*" or survey or " pre test and post test" or "pre-test and post-test" or "before and after" or "interrupted time series" or "time series" or "time-series" or "time points”

Contraceptive Use

microfinanc* or microcredit or microloan or "micro-financ*" or "micro-credit" or "micro-loan" or "micro financ*" or "micro credit" or "micro loan" or "small loan" or "small lend" or "micro enterpris*" or "micro entrepreneur”

AND

random* or "randomi*ed control trial" or "randomi*ed cluster trial" or study or analys* or cohort or "cross section*" or "cross-section*" or survey or "pre test and post test" or "pre-test and post-test" or "before and after" or "interrupted time series" or "time series" or "time-series" or "time points”

AND

"contraceptive*" or "contraception" or "reproductive" or gynaecolog* or gynecolog* or "birth control" or fertility

Nutrition

microfinanc* or microcredit or microloan or "micro-financ*" or "micro-credit" or "micro-loan" or "micro financ*" or "micro credit" or "micro loan" or "small loan" or "small lend" or "micro enterpris*" or "micro entrepreneur”

AND

nutrition OR malnutrition OR undernutrition OR under-nutrition OR underweight OR "MUAC" OR "mid-upper arm circumference" OR stunting OR "weight-for-age" OR "height for age" OR "height-for-age" OR wasting OR whz OR "Z score"

AND

random* or "randomi*ed control trial" or "randomi*ed cluster trial" or study or analys* or cohort or "cross section*" or "cross-section*" or survey or "pre test and post test" or "pre-test and post-test" or "before and after" or "interrupted time series" or "time series" or "time-series" or "time points"

IV CENTRAL

microfinanc* or microcredit or microloan or "micro-financ*" or "micro-credit" or "micro-loan" or "micro financ*" or "micro credit" or "micro loan" or "small loan" or "small lend" or "micro enterpris*" or "micro enterpreneur"

For peer review only - http://bmjopen.bmj.com/site/about/guidelines.xhtml

V LILAC

Microfinan$ OR microcredit$ OR microenterprise$ OR microentrepreneur$ OR microemp$

OR

(micro AND (enterprise$ ORcredit$ OR entrepreneur$ OR finan$ OR empres$ OR companhia$))

OR

Index microfinanzas

For peer review only

For peer review only - http://bmjopen.bmj.com/site/about/guidelines.xhtml

Supplement 2: REVISED NEWCASTLE-OTTAWA SCALE ADAPTED FOR CROSS-SECTIONAL STUDIES

Selection: (Maximum 5 stars) /6

1) Representativeness of the sample: **

a) Truly representative of the average in the target population. ** (all subjects or random sampling)

b) Somewhat representative of the average in the target population. * (non-random sampling)

c) Selected group of users.

d) No description of the sampling strategy.

2) Sample size:

a) Justified and satisfactory. *

b) Not justified.

3) Non-respondents:

a) Comparability between respondents and non-respondents characteristics is established, and the response rate is satisfactory. *

b) The response rate is unsatisfactory, or the comparability between respondents and non-respondents is unsatisfactory.

c) No description of the response rate or the characteristics of the responders and the non-responders.

4) Ascertainment of the exposure (risk factor):**

a) Validated – based on individual exposure. **

b) Non-validated measurement tool, but the tool is available or described – based on group exposure e.g. village level.*

c) No description of the measurement tool.

Comparability: (Maximum 2 stars) – /2

1) The subjects in different outcome groups are comparable, based on the study design or analysis. Confounding factors are controlled.

a) The study controls for the most important factors – age, education level, social status (select one). * *

b) The study displays data on the above factors comparing intervention and non-intervention groups but does not adjust*

For peer review only - http://bmjopen.bmj.com/site/about/guidelines.xhtml

c) No data on above factors collected

Outcome: (Maximum 2 stars) /3

1) Assessment of the outcome:

a) Assessment through self-reported anonymised questionnaires or blinded independent assessors. **

b) Record linkage. **

c) Systematic assessment without blinding or independent assessors and self-reported through interviewer. *

d) No description

2) Statistical test: -

a) The statistical test used to analyze the data is clearly described and appropriate, and the measurement of the association is presented, including confidence intervals and the probability level (p value). *

b) The statistical test is not appropriate, not described or incomplete.

Total # of stars: /11

This scale has been adapted from the Newcastle-Ottawa Quality Assessment Scale for cohort studies to perform a quality assessment of cross-sectional studies for this systematic review.

For peer review only

For peer review only - http://bmjopen.bmj.com/site/about/guidelines.xhtml

Supplement 3: Studies Excluded at Full-Text Screening

Reason For Exclusion	Number excluded
Contraceptive Use	
No results for outcome of interest	3
No comparison group included in the study	1
Childhood Nutrition	
No results for outcome of interest	4
Inappropriate measure provided at result stage (pooled result)	1
Study results already presented in another included article	1
Not traced in print and online editions of journal referenced	3
Female Empowerment	
No non-economic outcome presented in results	29
Outcome of interest only presented for intervention group but not for comparison group	1
No comparison group included in the study	12
Comparison group included but were also exposed to the intervention in some capacity	4
Exposure included other credit sources as well as microfinance	1
No empirical quantitative data presented (theoretical framework)	3
Study results already presented in another included article (including critiques of existing studies)	3
Study protocol only, no results provided	1
Not traced in print and online editions of journal referenced	3
Primary exposure of interest not microfinance	2
Not traced in the British library catalogue and other sources	2

For peer review only

For peer review only - http://bmjopen.bmj.com/site/about/guidelines.xhtml

Supplement 4: Impact of Microfinance on Household Decision Making Agency among Female Clients

Study	Measure of Decision Making Agency	Statistical measure of effect used	Results<br>(*p=p-value, n*=sample size)	Direction of effect
Angelucci *et al, 2015*[37]	Proportion of women who participate in any financial decision<br>Number of household issues women have a say on	Regression coefficients (SE)	0.008 (0.003)<br>*p*<0.01 *n*=12183<br>0.071 (0.030)<br>*p*<0.05 *n*=12379	Positive
Beaman *et al, 2014*[38]	Proportion of women free to decide about a) food expenses b) education expenses c) business.<br><br>d) Standardised index of intra-household decision making power derived from 3 individual measures	Regression coefficients (SE) for outcomes a-c<br><br>Regression coefficient (SE) for change in standardised index d (i.e. change in deviations from mean)	-0.006 (0.016) n=5425<br>0.010 (0.014) n=4440<br>0.012 (0.020) n=4180<br>0.02 (0.03) *n*=5425	No significant change
Mohindra *et al, 2008*[39]	Decision-making agency based on at least one situation of male decision making versus no male decision making in: seeking health care of a family member; daily household expenditures; child's education at school; family planning; voting in an election.	Adjusted odds ratio for early joiner (>2 years membership) compared to non-clients	0.90 (95%CI 0.53-1.74)<br>n=928	No significant change
Montgomery & Weiss, 2011[40]	Women between 15-40 asked about their involvement in family decisions regarding:<br>Child's schooling<br>Child's marriage<br>Whether to have another child<br>Repair/construction of house<br>Sale-purchase of livestock<br>Borrowing money	Logit, SE and ORs for female clients compared to non-clients	0.22 (0.30) OR=1.25<br>0.45 (0.34) OR=1.57<br>-0.01 (0.51) OR=0.99<br>0.36 (0.40) OR=1.43<br>-0.12 (0.57) OR=0.88<br>0.96 (0.38) OR=2.62**<br>-1.16 (1.08) OR=0.31<br>-0.60 (0.60) OR=0.55<br>**significant at 5% level *n*=2876	Positive change in involvement in decisions regarding borrowing money. No significant changes in other domains.

For peer review only - http://bmjopen.bmj.com/site/about/guidelines.xhtml

	Woman's participation in community political activity<br>Woman's decision to work outside home			
Sharif, 2004[42]	Degree of participation in decisions regarding:<br>Daily food purchases<br>Large purchases e.g. house, furniture<br>Health expenditure<br>Education of children<br>Marriage of children and social events<br>Fertility<br>Five point ranking given for each domain, 1 being least able, to 5, able to make decisions on her own	Means and standard deviation, Wilcoxon Z statistic and significance for difference between groups	Clients Non-clients<br>4.2 (1.15) 3.8 (1.45)<br>Z=1.83, *p*<0.05<br>3.1 (0.78) 2.7 (0.99)<br>Z=2.43, p<0.05<br>3.1 (0.87) 2.9 (0.91)<br>Z=0.68<br>3.2 (0.82) 2.9 (0.81)<br>Z=1.43, p<0.05<br>2.9 (0.61) 2.9 (0.67)<br>Z=2.14<br>2.9 (0.39) 2.9 (0.54)<br>Z=0.39	Positive change in decisions on purchase of food, large purchases and education of children
Tarozzi *et al*, *2015*[44]	Standardised index of fraction of decision across 20 domains women involved in:<br>All issues<br>Economic issues<br>(standardised using mean and SD of the outcome estimated from control areas at endline)	Regression coefficients (SEs) for change in standardised index (i.e. change in deviations from mean)	-0.043 (0.030)<br>n=10500 women<br>-0.038 (0.032)<br>n=10497 women	No significant change
Zaman, 1999[45]	Decision making agency:<br>If owns poultry % that can sell poultry independently<br>If owns livestock % that can sell livestock independently<br>If owns jewellry % that can sell jewellry independently<br>If has savings % can use savings independently	Coefficient estimates	-0.103 (*n*=980)<br>-0.178 (*n*= 103)<br>0.017 (*n*= 694)<br>-0.345*** (*n*=379)<br><br>***significant at 1% level	Positive change only in decisions on use of savings

For peer review only

For peer review only - http://bmjopen.bmj.com/site/about/guidelines.xhtml

PROSPERO
International prospective register of systematic reviews

National Institute for Health Research

Is microfinance associated with changes in women's empowerment and childhood nutrition, and does this vary by geographical region? A systematic review and meta-analysis

Wanjiku J Gichuru, Lisa Szatkowski, Alan Smyth, Shalini Ojha

Citation

Wanjiku J Gichuru, Lisa Szatkowski, Alan Smyth, Shalini Ojha. Is microfinance associated with changes in women's empowerment and childhood nutrition, and does this vary by geographical region? A systematic review and meta-analysis. PROSPERO 2015 CRD42015026018 Available from: http://www.crd.york.ac.uk/PROSPERO/display_record.php?ID=CRD42015026018

Review question

Through systematic review and, if possible, meta-analysis assess whether microfinance programmes are associated with changes in female empowerment and the wellbeing of women over the age of 15 years

Through systematic review and, if possible, meta-analysis assess whether microfinance programmes are associated with changes in use of a contraception method among women of reproductive age

Through systematic review and, if possible, meta-analysis assess whether microfinance programmes are associated with changes in childhood nutrition and whether this varies by the sex of the child

Searches

The Cochrane Central Register of Controlled Trials (CENTRAL),

Ovid MEDLINE,

EMBASE,

Latin American and Caribbean Health Sciences (LILACS),

ECONLIT.

An attempt will be made to access unpublished studies and dissertations through a search of grey literature through www.thesis.com.

The search will be limited to studies carried out after 1990.

No language restrictions will be imposed.

Types of study to be included

Cross-sectional surveys, cohort studies, controlled before-and-after studies, interrupted time series, quasi-experimental studies, randomised and non-randomised control/cluster trials.

Condition or domain being studied

For peer review only - http://bmjopen.bmj.com/site/about/guidelines.xhtml

Women's empowerment as measured by intimate partner violence, decision making agency, mobility and distinctly as uptake of a contraceptive method. Empowerment of women may also be linked to improved childhood nutrition. This will be measured by weight-for-age Z-scores, height-for-age Z-scores, weight-for-height Z scores and mid-upper arm circumference.

Participants/population

Inclusion: Women above the age of fifteen and children under-five for the outcome on childhood nutrition.

Exclusion: Men, children above five years

Intervention(s), exposure(s)

Intervention: Microfinance schemes defined as a combination of savings and credit services offered without physical collateral to a population thought to be poor or otherwise vulnerable through any organisation or institution.

The provider may be non-profit, e.g. NGO, self-help group (SHG), community-based organisation or microfinance bank, or a for-profit micro-finance institution, e.g., commercial bank.

Studies having an additional intervention will also be considered, provided that the primary intervention is microfinance.

Comparator(s)/control

Populations without any microfinance services or the same population prior to receiving microfinance. In studies with more than one comparator group, the group without microfinance will be considered as the main comparator.

Context

Developing countries in South Asia, sub Saharan Africa and Latin America and the Caribbean as defined by the Word Bank

Primary outcome(s)

1. Use of contraception

2. Childhood nutrition measured as the rate of malnutrition in girls and boys under-five years of age

3. Female empowerment and well-being

Timing and effect measures

1. Use of contraception method

2. Weight-for age Z score, Height-for-age Z score, Weight-for-height Z score, mid-upper arm circumference

3. Intimate partner violence (IPV), decision making agency,mobility

Secondary outcome(s)

None

Data extraction (selection and coding)

The search will be conducted and subsequent papers reviewed for eligibility independently by two researchers in three stages; title, abstract and full-text.

A data extraction form will be completed for each selected study by one researcher under the following sub-headings; publication details, study details, nature of study, intervention and results. The data extraction forms will then be reviewed by the second researcher. This is to be used in further analysis and synthesis of the data.

Any disparities will be solved be mutual consensus between the two primary researchers. If this is not possible, the input of the third researcher will be sought.

Risk of bias (quality) assessment

The selected studies will be assessed for risk of bias by two researchers using the Cochrane Collaboration's tool for assessing risk of bias in randomised controlled trials and for quality by the Newcastle-Ottawa Quality Assessment Scale in non-randomised studies.

Any disparities will be resolved be mutual consensus between the two primary researchers. If this is not possible, the input of the third researcher will be sought.

Strategy for data synthesis

Outcome measures will be extracted from the studies and used in the meta-analyses. The studies providing an appropriate measure of effect will be weighted using a quality rating system and then stratified by quality score. A descriptive analysis will be done for studies providing quantitative outcome measures not suitable for meta-analysis.

A fixed-effects or a random-effects model will be used in pooling of the data and a suitable method of estimating variance in studies will be applied. The summary estimate of the effect size will be done in each stratum according to quality score, i.e. high, medium and low quality score, and statistical tests (I-squared) used to check for heterogeneity.

Analysis of subgroups or subsets

A sub-group analysis of the measures of effect chosen will be done according to region to detect any variations between regions. The three regions will be geographically specified as Sub-Saharan Africa, South Asia and South America. The results will be presented by tables within the text of the review or if possible in forest plots in the meta-analysis

Contact details for further information

Dr Gichuru

wanjiku.gichuru@gmail.com

Organisational affiliation of the review

University of Nottingham

www.nottingham.ac.uk

Review team members and their organisational affiliations

Dr Wanjiku J Gichuru. University of Nottingham
Dr Lisa Szatkowski. University of Nottingham
Professor Alan Smyth. University of Nottingham
Dr Shalini Ojha. University of Nottingham

Anticipated or actual start date

20 April 2015

Anticipated completion date

08 December 2015

Funding sources/sponsors

The Commonwealth Scholars and Fellows Scheme funded the Masters' course of which this review formed part of the dissertation

Conflicts of interest

None known

Language

English

Country

England

Stage of review

Review_Completed_not_published

Subject index terms status

Subject indexing assigned by CRD

Subject index terms

Female; Humans; Nutritional Status; Power (Psychology)

Date of registration in PROSPERO

09 September 2015

Date of publication of this version

12 January 2016

Revision note for this version

Update to reflect the completion of the review.

Details of any existing review of the same topic by the same authors

Stage of review at time of this submission

Stage	Started	Completed
Preliminary searches	Yes	Yes
Piloting of the study selection process	Yes	Yes
Formal screening of search results against eligibility criteria	Yes	Yes
Data extraction	Yes	Yes
Risk of bias (quality) assessment	Yes	Yes

For peer review only - http://bmjopen.bmj.com/site/about/guidelines.xhtml

Stage	Started	Completed
Data analysis	Yes	Yes

Revision note

Update to reflect the completion of the review.

Versions

09 September 2015

12 January 2016

PROSPERO
This information has been provided by the named contact for this review. CRD has accepted this information in good faith and registered the review in PROSPERO. CRD bears no responsibility or liability for the content of this registration record, any associated files or external websites.

PRISMA 2009 Checklist

Section/topic	#	Checklist item	Reported on page #
**TITLE**			
Title	1	Identify the report as a systematic review, meta-analysis, or both.	1
**ABSTRACT**			
Structured summary	2	Provide a structured summary including, as applicable: background; objectives; data sources; study eligibility criteria, participants, and interventions; study appraisal and synthesis methods; results; limitations; conclusions and implications of key findings; systematic review registration number.	2
**INTRODUCTION**			
Rationale	3	Describe the rationale for the review in the context of what is already known.	4
Objectives	4	Provide an explicit statement of questions being addressed with reference to participants, interventions, comparisons, outcomes, and study design (PICOS).	5
**METHODS**			
Protocol and registration	5	Indicate if a review protocol exists, if and where it can be accessed (e.g., Web address), and, if available, provide registration information including registration number.	5
Eligibility criteria	6	Specify study characteristics (e.g., PICOS, length of follow-up) and report characteristics (e.g., years considered, language, publication status) used as criteria for eligibility, giving rationale.	5
Information sources	7	Describe all information sources (e.g., databases with dates of coverage, contact with study authors to identify additional studies) in the search and date last searched.	6
Search	8	Present full electronic search strategy for at least one database, including any limits used, such that it could be repeated.	Supplement 1
Study selection	9	State the process for selecting studies (i.e., screening, eligibility, included in systematic review, and, if applicable, included in the meta-analysis).	7
Data collection process	10	Describe method of data extraction from reports (e.g., piloted forms, independently, in duplicate) and any processes for obtaining and confirming data from investigators.	7
Data items	11	List and define all variables for which data were sought (e.g., PICOS, funding sources) and any assumptions and simplifications made.	6
Risk of bias in individual studies	12	Describe methods used for assessing risk of bias of individual studies (including specification of whether this was done at the study or outcome level), and how this information is to be used in any data synthesis.	Supplement 2
Summary measures	13	State the principal summary measures (e.g., risk ratio, difference in means).	7

For peer review only - http://bmjopen.bmj.com/site/about/guidelines.xhtml

PRISMA 2009 Checklist

Synthesis of results	14	Describe the methods of handling data and combining results of studies, if done, including measures of consistency (e.g., I^2) for each meta-analysis.	7

Section/topic	#	Checklist item	Reported on page #
Risk of bias across studies	15	Specify any assessment of risk of bias that may affect the cumulative evidence (e.g., publication bias, selective reporting within studies).	14
Additional analyses	16	Describe methods of additional analyses (e.g., sensitivity or subgroup analyses, meta-regression), if done, indicating which were pre-specified.	7
**RESULTS**			
Study selection	17	Give numbers of studies screened, assessed for eligibility, and included in the review, with reasons for exclusions at each stage, ideally with a flow diagram.	7 [Figure 1]
Study characteristics	18	For each study, present characteristics for which data were extracted (e.g., study size, PICOS, follow-up period) and provide the citations.	8 – 11 Table 2
Risk of bias within studies	19	Present data on risk of bias of each study and, if available, any outcome level assessment (see item 12).	8 – 11 [Table 2]
Results of individual studies	20	For all outcomes considered (benefits or harms), present, for each study: (a) simple summary data for each intervention group (b) effect estimates and confidence intervals, ideally with a forest plot.	12 - 14
Synthesis of results	21	Present results of each meta-analysis done, including confidence intervals and measures of consistency.	12 [Figure 2]
Risk of bias across studies	22	Present results of any assessment of risk of bias across studies (see Item 15).	14
Additional analysis	23	Give results of additional analyses, if done (e.g., sensitivity or subgroup analyses, meta-regression [see Item 16]).	
**DISCUSSION**			
Summary of evidence	24	Summarize the main findings including the strength of evidence for each main outcome; consider their relevance to key groups (e.g., healthcare providers, users, and policy makers).	15
Limitations	25	Discuss limitations at study and outcome level (e.g., risk of bias), and at review-level (e.g., incomplete retrieval of identified research, reporting bias).	17
Conclusions	26	Provide a general interpretation of the results in the context of other evidence, and implications for future research.	18
**FUNDING**			
Funding	27	Describe sources of funding for the systematic review and other support (e.g., supply of data); role of funders for the systematic review.	2

For peer review only - http://bmjopen.bmj.com/site/about/guidelines.xhtml

PRISMA 2009 Checklist

From: Moher D, Liberati A, Tetzlaff J, Altman DG, The PRISMA Group (2009). Preferred Reporting Items for Systematic Reviews and Meta-Analyses: The PRISMA Statement. PLoS Med 6(6): e1000097. doi:10.1371/journal.pmed1000097

For more information, visit: **www.prisma-statement.org**.

For peer review only

For peer review only - http://bmjopen.bmj.com/site/about/guidelines.xhtml

BMJ Open

Is microfinance associated with changes in women's wellbeing and children's nutrition? A systematic review and meta-analysis

Journal:	*BMJ Open*
Manuscript ID	bmjopen-2018-023658.R2
Article Type:	Research
Date Submitted by the Author:	13-Nov-2018
Complete List of Authors:	Gichuru, Wanjiku; University of Nottingham, Academic Division of Child Health;<br>Ojha, Shalini; University of Nottingham, Division of Graduate Entry Medicine; University of Nottingham, Academic Division of Child Health<br>Smith, Sherie; University of Nottingham School of Medicine, Division of Child Health, Obstetrics and Gynecology<br>Smyth, Alan; University of Nottingham, Division of Child Health, Obstetrics & Gynaecology,<br>Szatkowski, Lisa; University of Nottingham, Division of Epidemiology and Public Health
**Primary Subject Heading**:	Global health
Secondary Subject Heading:	Evidence based practice, Health policy, Paediatrics
Keywords:	Microfinance, Female empowerment, Community child health < PAEDIATRICS, Global health

SCHOLARONE™
Manuscripts

Is microfinance associated with changes in women's wellbeing and children's nutrition? A systematic review and meta-analysis

Wanjiku Gichuru[1] MPH (International Health), Shalini Ojha[2] PhD, Sherie Smith[3], Alan R Smyth[3] MD, Lisa Szatkowski PhD[1]

[1]University of Nottingham, Division of Epidemiology and Public Health, Clinical Sciences Building, Nottingham City Hospital, Nottingham, UK, NG5 1PB

[2]University of Nottingham, Division of Graduate Entry Medicine, Derby Medical School, Derby, DE22 3DT

[3]Division of Child Health, Obstetrics and Gynaecology, E Floor, East Block, Queen's Medical Centre, Nottingham, UK, NG7 2UH

Corresponding author: shalini.ojha@nottingham.ac.uk

Competing interests statement

There are no competing interests for any authors.

Data sharing agreement

This is a secondary analysis of published data. We do not hold any unpublished data from the study. Further information about the data analysis can be obtained by contacting the corresponding author.

For peer review only

ABSTRACT

Background: Microfinance is the provision of savings and small loans services, with no physical collateral. Most recipients are disadvantaged women. The social and health impacts of microfinance have not been comprehensively evaluated.

Objective: To explore the impact of microfinance on contraceptive use, female empowerment and children's nutrition in South Asia, Sub-Saharan Africa and Latin America and the Caribbean.

Design: We conducted a systematic search of published and grey literature (1990-2018), with no language restrictions. We conducted meta-analysis, where possible, to calculate pooled odds ratios. Where studies could not be combined we described these qualitatively.

Data Sources: EMBASE, MEDLINE, LILACS, CENTRAL and ECONLIT were searched (1990-June 2018).
Eligibility Criteria: We included controlled trials, observational studies, and panel data analyses investigating microfinance involving women and children.

Data extraction and synthesis: Two independent reviewers extracted data and assessed risk of bias. The methodological quality of included studies was assessed using the Cochrane Risk-of-Bias tool for controlled trials and quasi-experimental studies and a modified Newcastle Ottawa Scale (NOS) for cross-sectional surveys and analyses of panel data. Meta-analyses were conducted using STATA v15 (StataCorp, College Station, TX).

Results: We included 27 studies. Microfinance was associated with a 64% increase in the number of women using contraceptives [OR 1·64, 95%CI 1.45 1.86]. We found mixed results for the association between microfinance and intimate partner violence. Some positive changes were noted in female empowerment. Improvements in children's nutrition were noted in three studies.

Conclusion: Microfinance has the potential to generate changes in contraceptive use, female empowerment and children's nutrition. It was not possible to compare microfinance models due to the small numbers of studies. More rigorous evidence is needed to evaluate the association between microfinance and social and health outcomes.

Funding: "This work was supported by the Medical Research Council [grant number MR/M021904/1], UK."

PROSPERO registration number: CRD42015026018

For peer review only

Strengths of the study:

A critical evaluation of the limited evidence of the effects of microfinance on social and health outcomes.

Encompasses all regions of the low-and-middle income countries where microfinance is most likely to impact health and wellbeing of vulnerable populations.

Broad search terms used to capture all types of microfinance and a range of terminologies for the chosen outcomes.

No language restrictions – captured all Latin American literature which is vital in the field of microfinance.

Limitations of the study:

We found few randomised controlled trials in the field and relied upon the inclusion of quasi-experimental studies.

For peer review only

For peer review only - http://bmjopen.bmj.com/site/about/guidelines.xhtml

INTRODUCTION

Rationale: Microfinance is the provision of financial services, including savings, deposit, and credit services, to the poor[1]. The term was first used in the early 1990s though schemes have been in operation in the developing world since the 1970s[2]. ‘Microfinance’ is subtly distinct from ‘microcredit,’ which refers to only small loans to poor people without a savings component. Microfinance may also include provision of micro-insurance as an “add on” to the loans and saving component. Distinct characteristics of microfinance schemes are that they are short-term, have simple application procedures and do not require loan security but instead rely on a ‘collective’ guarantee from an enrolled group[3]. The purpose of microfinance is that the loans should reach the poor and move them out of poverty[4].

The financial viability of microfinance programmes may be assessed by factors such as loan size, number of loans per person and repayment rates. One of the first studies to evaluate the economic impact of microfinance on participants was a quasi-experimental survey from Bangladesh[5]. This showed a reduction in moderate and extreme poverty and an increase in annual household expenditure of 18% among female, and 11% among male, borrowers. Institutions such as the World Bank, International Monetary Fund and the United Nations have since supported microfinance. There are currently over 3,500 microfinance institutions providing financial support to 170 million people worldwide, mostly in South Asia, Sub-Saharan Africa (SSA), Latin America and the Caribbean (LAC)[6] .

There is an emerging body of literature, including both experimental and quasi-experimental studies, looking at the social and health outcomes of microfinance programmes. In some cases, individual studies from the same region have reported contradictory results. For example, one study in Ghana demonstrated that combining microfinance and nutritional education led to improved indicators of children’s nutrition in the intervention group[7], while a study in Ethiopia failed to demonstrate any difference in nutrition status between the children of clients and non-clients[8]. The two studies used different nutritional outcome measures as well as different age limits which makes synthesis of the findings difficult. Similarly, a study from Bangladesh reported improved female empowerment fifteen years later[9], but there was no significant effect in a study in Hyderabad, India[10]. Most available studies are small and have insufficient power to detect small changes in outcomes. Therefore, this systematic review brings together results from existing studies to assess whether receiving microfinance is associated with changes in women’s empowerment and the well-being of their children.

Objectives: We aimed to evaluate the impact of microfinance schemes on health and social outcomes, specifically female contraceptive use and measures of female empowerment (intimate partner violence, decision making ability and mobility), as well as the effects on child nutrition.

For peer review only

For peer review only - http://bmjopen.bmj.com/site/about/guidelines.xhtml

METHODS

The protocol for this review is registered with PROSPERO, registration number CRD42015026018, and is available from http://www.crd.york.ac.uk/PROSPERO(Supplementary file: Gichuru et al. PROSPERO protocol).

Eligibility Criteria: We included all controlled trials, observational studies, and analyses of panel data from South Asia, SSA and LAC[11] in women over the age of 15 and children under five. We included quasi-experimental studies (empirical studies used to estimate the causal impact of an intervention without randomisation). In most cases, panel data were longitudinal or "before and after" studies. We also put in a geographical limitation to studies in countries within three World Bank regions with the highest number of developing countries[12]. Studies were included where the microfinance intervention comprised both savings and credit services, without physical collateral, to a poor or otherwise vulnerable population. Studies where microfinance was introduced and measured for expected change in outcome were included. Studies where an additional intervention was delivered in addition to microfinance were also included, provided that there was an intervention group where a microfinance intervention was assessed in comparison to the control group. In studies with more than one comparison group, the group without microfinance was considered as the main comparator. Studies were excluded where there were no suitable comparison data – either from a population who had not received microfinance, or pre-intervention data from those who went on to receive microfinance.

Patient and Public Involvement: There was no PPI involvement in the design or conduct of this review. The results were presented and discussed at a dissemination workshop in Patna, Bihar.
We conducted a workshop "Women's Empowerment and Child Health: Exploring the Impact of Rojiroti Microfinance in Poor Communities in Bihar- An Indo-UK collaboration" in Patna, India on May 22, 2018. It was attended by more than 30 women who participate in microfinance, and a wide range of local stakeholders. The results of this review and other work were presented and discussed it this meeting and women's views were noted to enable further research in this area.

Outcome measures: Table 1 lists the outcome measures used to assess the impact of microfinance. The Grameen foundation proposed three variables as indicators of the social performance of microfinance[13]: female use of contraceptives, female empowerment and children's nutrition. [14-19]
The World Health Organisation (WHO) considers the health and wellbeing of women to be tied to their ability to access healthcare and have a say in decisions related to their health[14]. Improved health status could therefore be a possible consequence and proxy indicator of female empowerment. The WHO provides some standardised measures for use in assessing the health of women in a population. These include deaths from pregnancy-related complications, uptake of contraceptives and utilisation of perinatal services[14 15]. Uptake of contraceptives is one of the measures proposed by the Grameen Foundation. [16]

For peer review only - http://bmjopen.bmj.com/site/about/guidelines.xhtml

For peer review only

Due to the broadness of the term "female empowerment", indicators collated from definitions used by the WHO[14 15] and the UN Millennium taskforce on gender equality[16] and also from literature on social measures of female empowerment[17 19] were used to inform the selection of the three outcome measures of female empowerment used in this systematic review. These were self-reported intimate partner violence, decision-making ability and mobility.

Table 1: Definitions of outcome measures

**Contraceptive Use**<br>Self-reported use of any contraceptive method to prevent or plan for pregnancy.
**Female empowerment**<br>**Intimate Partner Violence (IPV):** Self-reported intimate partner violence described as physical, sexual, or psychological harm by a current or former partner[20].<br>**Sole decision-making ability:** Self-reported independent decision-making ability where the woman is not the head of household; including but not limited to, household expenditure, children's education or as a combined measure of empowerment as defined by individual study authors.<br>**Mobility:** Self-reported freedom to travel out of the village or to attend social events without the permission or accompaniment of a male relative.
**Children's nutrition**<br>Standard nutritional measures for children aged <5 as defined by the WHO Global Database on Child Growth and Malnutrition (WHO). Moderate undernutrition (malnutrition) was defined as a Z-score <-2 but > -3 standard deviations (SD) from the mean. Severe undernutrition (malnutrition) was defined as a Z-score<-3 SD from the mean.<br>**Weight-for-age Z-score (WAZ)**<br>**Height (or length)-for-age Z-score (HAZ)** – the most indicative measure of chronic undernutrition over a prolonged period leading to growth retardation known as stunting.<br>**Weight-for-height (or length) (WHZ)** – most indicative measure of acute undernutrition known as wasting. This distinguishes short children of normal weight and tall children of low weight that may not be captured by WAZ or HAZ.<br>**Body mass index (BMI)-for-age Z-score (BAZ).**<br>**Mid-upper arm circumference (MUAC)** – an absolute measure where a MUAC <11.5cm in children 6-60 months is considered as severe acute malnutrition (wasting) and MUAC 11-12.5cm moderate acute malnutrition.

Information sources:

EMBASE, MEDLINE, LILACS, CENTRAL and ECONLIT were searched from 1990 (when microfinance was first described[2] to 9th September 2015. These were accessed through www.theses.com, and the references of included studies were tracked to identify other relevant papers. No language restrictions were applied. Searches were conducted using MESH headings and free text, as described in Supplement 1.

For peer review only

For peer review only - http://bmjopen.bmj.com/site/about/guidelines.xhtml

Study selection, data extraction and quality assessment: Two authors (WG and LS) independently screened the titles and abstracts of retrieved studies against the study eligibility criteria. The search was updated in June 2018. For the updated search, two authors again screened the titles and abstracts (SS and SO) of the retrieved studies and two authors (SS and WG) screened the full text and extracted data, where possible. Discrepancies were resolved by discussion and duplicates removed. Retrieved studies were translated into English, where necessary, and data were extracted by the two authors independently using a standard data extraction form. The methodological quality of included studies was assessed independently by WG and LS using the Cochrane Risk-of-Bias tool[21] for controlled trials and quasi-experimental studies and a modified Newcastle Ottawa Scale (NOS)[22] for cross-sectional surveys and analyses of panel data (Supplement 2).

Data synthesis and analysis: Meta-analyses were conducted using STATA v15 (StataCorp, College Station, TX) to pool the measures of effects from eligible studies. Where available, adjusted measures of effect were preferred over unadjusted measures. Statistical significance was set at a *p*-value of <0·05. A random effects model was initially fitted for each meta-analysis. For studies with low heterogeneity analysis was repeated using a fixed effects model. Publication bias was assessed using funnel plots and Egger's asymmetry test (where at least five studies were available). Descriptive synthesis was carried out where studies could not be meta-analysed.

RESULTS

Study selection: A total of 5659 titles were identified across the three groups of outcome measures, which reduced to 5298 after removal of duplicates. From these, 5023 titles were excluded as not being on microfinance as agreed mutually by two authors; 275 abstracts were subsequently screened. A total of 17 abstracts were translated for the authors to review. Each author screened the abstracts individually then came together to compare findings. The authors disagreed on 2 abstracts under contraceptive use, 4 under children's nutrition, and 36 under female empowerment. These were discussed further jointly and agreed upon by mutual consensus. A total of 97 progressed to full-text screening. Reference tracking identified 2 additional studies for full-text screening. We included 27 articles in the final review (Figure 1). Seventy titles were excluded after full-text screening with reasons for exclusion outlined in Supplement 3. Of the 27 included articles, 4 reported on contraceptive use, 5 on childrens' nutrition and 18 on indicators of female empowerment. Eighteen were from South Asia, 8 from SSA and 1 from LAC. Table 2 summarises the characteristics of the included studies.

For peer review only - http://bmjopen.bmj.com/site/about/guidelines.xhtml

Table 2: Summary of included studies

Author, year of publication	Study design	Study setting (urban/rural, country, region)	Number of participants included in analysis	Data collection time points	Intervention provider	Services provided	Comparison group (MF, microfinance)	Outcome measured	Quality assessment score
**Studies with outcome measure of contraceptive use**									
Desai & Tarozzi, 2011[23]	Baseline and follow up surveys from a panel of villages: the impact of the program was estimated using a difference-in difference approach	Rural, Ethiopia, Sub-Saharan Africa	7712 women at baseline; 7949 women at follow-up	2003 and 2006	CBO supported by an international NGO	Credit and savings in group-lending model, with additional family planning (FP) education	Two comparison groups – 1. No MF or FP (used as the controls in this review) 2. FP only	Married women aged 15-49 reporting current use of any form of contraception	NOS 7/11
Pitt & Khandker, 1996[24]	Quasi-experimental study using an econometric approach to account for non-random placement of credit programs and unmeasured village and household attributes	Rural, Bangladesh, South Asia	1731 women	1991, 1992	MFI - Grameen, BRAC, BRDC	Credit and savings in group lending model	No MF	Married women aged 14-50 reporting current use of any form of contraception	NOS 4/11
Steele et al, 2001[25]	Quasi-experimental study. Analysis accounted for non-random placement and self-selection by taking a random sample of women and classifying them according to their eligibility for program membership to form target and non-target groups and considered demographic and socioeconomic variables in the analysis	Rural, Bangladesh, South Asia	6456 women at baseline; 5696 women at follow-up	1993 and 1995	International NGO and MFI-ASA	Credit and savings in group lending model	Two comparison groups – 1. No MF (used as the controls in this review) 2. Savings with no credit	Married women reporting current use of any form of contraception	NOS 7/11
Murshid &Ely 2017[26]	Quasi-experimental study – a logistic regression model adjusted for socio-economic variables	Rural, Bangladesh, South Asia	7325 women	2011	Grameen, BRAC, ASA, Proshika, Mother's Club, BRDB or other	Credit and savings in group lending model	Non participants	Married women aged 14-50 reporting any form of contraception	NOS 7/11
**Studies with outcome measure of female empowerment**									
Ahmed, 2005[27]	Data subset from cross sectional survey.	Not reported, Bangladesh,	2044 women	1999	MFI - BRAC	Credit and savings in group lending	Two comparison groups –	All women reporting either physical or	NOS 7/11

For peer review only

	Conduced bivariate analysis to characterize group level differences followed by a logistic regression with variables at the individual and household levels and one "BRAC membership status" variable to account for eligibility, savings and credit	South Asia				model with unspecified skilled training offered to some clients	1. No MF (used as the controls in this review)<br>2. Skilled training and MF	verbal abuse between herself the client and her husband in the preceding 4 months	
Bajracharya & Amin, 2013[28]	Cross sectional survey – used propensity score matching to address selection bias	Rural and urban, Bangladesh, South Asia	4195 women	2007 Demographic and Health Survey	Any MFI - Grameen, BRAC, ASA, Prosika	Credit and Savings in group lending Model	No MF	Married women reporting any form of violence by her partner in preceding 12 months	NOS 8/11
Dalal et al, 2013[29]	Cross sectional Survey – used chi-squared test to examine difference in IPV exposure and microfinance and demographic variables (age, residence, education, religion and wealth index)	Rural and urban, Bangladesh, South Asia	4465 women	2007 Demographic and Health Survey	Any MFI - Grameen, BRAC, ASA, Proshika	Credit and Savings in group lending Model	No MF	All women reporting any form of violence by her partner in preceding 12 months	NOS 8/11
Murshid et al. 2016[30]	Cross sectional Survey data was used to investigate association between microfinance and domestic violence with predictor variables including economic status, decision making power and demographic variables	Rural and urban, Bangladesh, South Asia	4163 women	2007 Demographic and Health Survey	Any MFI - Grameen, BRAC, ASA, Proshika	Credit and Savings in group lending Model	No MF	Conflicts Tactics Scale based on the battery of questions that asked respondents whether they experienced a number of violent acts that constituted physical and sexual violence	NOS 8/11
Pronyk et al, 2006[31]	Cluster RCT: per-protocol analysis. As only 8 villages were randomised, baseline imbalances were adjusted prior to analysis	Rural, South Africa, Sub-Saharan Africa	538 women (290 intervention, 248 control)	2001, 2005	Local NGO	Credit and savings in group lending model with additional life skills training	No MF	All women reporting intimate partner violence in preceding 12 months	Cochrane Risk-of-Bias – High
Schuler et al, 1996[32]	Cross sectional survey. Conducted multivariate analysis using a logistic regression model with independent variables age, education, religion, whether respondent had any surviving sons or	Rural, Bangladesh, South Asia	1225 women	1992	MFI - Grameen and BRAC	Credit and savings in group lending model	No MF	Women reporting physical beating by husband in the preceding 12 months	NOS 7/11

For peer review only - http://bmjopen.bmj.com/site/about/guidelines.xhtml

	daughters, geographic region, economic level of household, respondent's contribution to family support and, exposure to credit programs.								
Angelucci et al, 2015[33]	Cluster RCT: intent-to-treat analysis on all respondents.	Rural, Mexico, Latin and Central America	1823 women	2009-12	MFI – Compartamos Banco	Credit and savings in group lending model	No MF	Decision-making ability: participation in financial decisions and household issues by non-single women aged 18-60 who are not the only adult in their household.	Cochrane Risk-of-Bias – High
Banerjee et al, 2015[10]	Cluster RCT: intent-to-treat analysis: constructed an equally weighted average z-score of 16 social outcomes to detect any difference.	Urban, India, South Asia	6862 women at first follow-up; 6142 women at second follow-up	2005, 2010	MFI – Spandana	Credit and savings in group lending model	No MF	Index of empowerment encompassing scores across 16 domains, covering decision making, levels of health and education expenditure and school enrollment	Cochrane Risk-of-Bias – High
Beaman et al, 2014[34]	Cluster RCT – intention to treat analysis. The econometric baseline characteristics and variable used in the randomisation process such as household and village characteristics.	Rural, Mali, Sub-Saharan Africa	5425 women	2009, 2012	SHG with NGO support	Credit and savings in self-help group model	No MF	Decision making ability: women's freedom to decide about food and educational expenses and take decisions about business. Index of intra-household decision making power combining individual measures	Cochrane Risk-of-Bias – High
Karlan 2017[35]	Cluster RCT<br>- A polled model controlling for baseline values and district was estimated by an "intention to treat" method.	Rural: Ghana, Malawi, and Uganda	15,000 households	Baseline 2008 to survey at endline in 2011	Cooperative for Assistance and Relief Everywhere (CARE)	Village savings and loan associations	No MF	Decision making ability: women's empowerment index capturing self-reported influence on household decisions, particularly in relation to food expenses for the household, education and health care expenses for the	Cochrane Risk-of-Bias – High

For peer review only - http://bmjopen.bmj.com/site/about/guidelines.xhtml

								children, business expenses if the household operates a business and the women's ability to visit friends	
Mohindra et al, 2008[36]	Cross sectional survey. A three step model including only SHG participation, socioeconomic characteristics and caste was examined with a goodness-of-fit test and odds ratios.	Rural, India, South Asia	928 women	2003	SHG with NGO support	Credit and savings in self-help group model	No MF	Decision-making ability – whether women aged 18-59 reported at least 1 situation (of 5 asked) in which her husband or a male relative was the sole decision-maker	NOS 7/11
Montgomery & Weiss, 2011[37]	Cross sectional survey: analysis accounted for income variables, consumption-expenditure variables, and household characteristics and explored differential effects on urban and rural households	Rural and urban, Pakistan, South Asia	2876 women	2005	Commercial MFI - Khushali	Credit and savings in group lending model	No MF	Decision making ability – women between 15-40 asked whether their opinion is taken into account in a series of household decisions	NOS 7/11
Pitt et al, 2003[9]	Quasi-experimental study using econometric methods similar to Pitt and Kandker et al.[5]	Rural, Bangladesh, South Asia	2074 women	1991/1992, 1998/1999	MFI – Grameen, BRAC, BRDC, ASA	Credit and savings in group lending model	No MF	Empowerment score combining empowerment indicators across several domains of decision making, discussion, finance and mobility	NOS 7/11
Rahman et al, 2009[38]	Quasi-experimental cross-sectional survey. Considered age, education level, spouse's age and education level, household income, asset accumulation and locality in the analysis.	Rural and urban, Bangladesh, South Asia	571 recruited and analysed	Not indicated	MFI - Grameen and BRAC	Credit and Savings in group lending model	No MF	Mobility index; empowerment index	NOS 6/11
Sharif, 2004[39]	Cross sectional survey data were used for econometric analysis with a range of socioeconomic and demographic variables.	Not reported, Bangladesh, South Asia	483 women	1997	MFI - ASA	Credit and savings in group lending model	No MF	Decision making ability – Likert-type responses on women's extent of	NOS 7/11

For peer review only - http://bmjopen.bmj.com/site/about/guidelines.xhtml

								decision making across 6 domains	
Swain & Wallentin, 2009[40]	Quasi-experimental cross-sectional survey. Used the robust maximum likelihood method.	Rural and urban, India, South Asia	961 women	2000 and 2003	SHG with MFI linkage	Savings at group level and credit from MFI in group lending model	No MF	Empowerment score	NOS 5/11
Tarozzi et al, 2015[41]	Cluster RCT Panel of villages data for used for an intent-to-treat analysis to identify the impact of giving access to microcredit rather than actual borrowing	Rural, Ethiopia, Sub-Saharan Africa	6412 households at baseline; 6263 households at follow-up	2003 and 2006	CBOs supported by international NGO	Credit and savings in group lending model	No MF	Decision making ability – fraction of decisions across 20 domains women aged 15-49 were involved in making	Cochrane Risk-of-Bias -High
Zaman, 1999[42]	Cross sectional survey data were used in a multivariate analysis with considerations for the number of eligible households in the village, membership length, and socio-economic differences.	Rural, Bangladesh, South Asia	1568 women	1995	MFI - BRAC	Credit and savings in group lending model	No MF	Decision making ability	NOS 2/11
**Studies with outcome measures of children's nutrition**									
Abubakari et al, 2014[43]	Cross sectional survey – analysis accounted for food acquisition behaviours and demographic characteristics of the households	Rural, Ghana, Sub-Saharan Africa	180 children	2011	Village Savings and Loans Association	Credit and savings in self-help group model	No MF	Anthropometric measurement of nutritional status in children <5 years based on HAZ scores: >-2 well nourished; <-2 to -3 moderate malnutrition; <-3 severe malnutrition	NOS 4/10
Doocy et al, 2005[8]	Cross sectional survey with community controls who were matched by sex and selected by proximity of residence via systematic random sampling.	Rural and urban, Ethiopia Sub-Saharan Africa	608 children	2003	NGO - WISDOM	Credit and Savings in group lending model	Two comparison groups:<br>1. No MF (used as the controls in this review)<br>2. New clients <1 cycle of MF	Anthropometric measurement of nutritional status in children aged 6-59 months based on MUAC: <11cm severe malnutrition; 11-12.5cm moderate malnutrition	NOS 6/11
Friesen et al, 2012[44]	Cross sectional survey. Analysis included socioeconomic and demographic factors	Rural and urban, Ghana, Sub-Saharan Africa	204 children	June to August 2011	Local microfinance bank (previously	Credit and savings in group lending model	No MF	Anthropometric measurement of nutritional status in children aged 6-23	NOS 7/11

For peer review only - http://bmjopen.bmj.com/site/about/guidelines.xhtml

	including household and maternal characteristics and child's age and sex.				with NGO support)			months based on proportion underweight (WAZ<-2), stunted (LAZ<-2) and wasted (WLZ<-2)	
Marquis et al, 2015[7]	Quasi-experimental design with longitudinal follow-up. Bivariate analysis between anthropometric measures and explanatory variables and sensitivity analysis was performed to examine within subject variations	Rural, Ghana, Sub-Saharan Africa	608 caregivers with children	Approximately 4-monthly between April 2006 and Dec 2007	Credit and savings association	Credit and savings in self-help group model with additional health, nutrition and entrepreneur education	No MF	Anthropometric measurement of nutritional status in children aged 2-5 years based on WAZ, HAZ and BAZ scores	Cochrane Risk-of-Bias – High
Ojha et al, 2017[45]	Cluster randomized controlled trial with cross sectional follow up and intention to treat analysis	Rural, India, South Asia	1377 children	August 2013 to March 2016	Rojiroti microfinance programme	Savings and credit in peer led self-help groups	No MF	Anthropometric measures of children 0-5 years of age WHZ, HAZ, WAZ, MUAC	Cochrane Risk of Bias - High

*MF – Microfinance , FP- Family Planning, SHG – self-help group

For peer review only

For peer review only - http://bmjopen.bmj.com/site/about/guidelines.xhtml

Nature of the microfinance interventions evaluated: The most common microfinance model was group-lending as provided by formal microfinance institutions (MFIs)[9 10 24-29 32 33 38 39 41 44 45] and community-based organisations (CBOs)[7 8 23 31 35 41]. MFIs required clients to be women above the age of eighteen, own less than 0·5 decimals of land (435 square feet) and have at least one household member in casual employment. Self-help groups and CBOs had fewer eligibility criteria but with greater emphasis on accumulation of savings [7 26 30 34 36 40 43 45]. In some studies microfinance was coupled with additional social and health interventions[7 23 27 31].

Findings of Studies by Outcome

Contraceptive Use

Four studies[5 23 25 26] evaluated the impact of microfinance on self-reported use of contraception using data from household cross-sectional surveys. One study[23] evaluated an intervention that combined microfinance with family planning education in Ethiopia. The other 3 studies[24-26] recruited clients from non-commercial MFIs in Bangladesh.

The impact of microfinance in the Ethiopian study was estimated at the level of the *kebele* (a cluster of villages) and showed no significant change in the proportion of married women reporting contraceptive use; individual-level estimates of the impact of microfinance were not available. A fixed-effects meta-analysis of individual-level data from the three Bangladeshi studies showed that women participating in microfinance were 64% more likely to report contraceptive use than non-clients [OR=1.64, 95%CI 1.45 1.86; Figure 2]. There was no heterogeneity between the studies, which is plausible given the similarity in the average age and socio-economic status of participants.

Female empowerment

Seventeen studies evaluated the impact of microfinance on female empowerment. Eight were conventional cross-sectional studies[27-30 32 36 37 39 42], 3 were quasi-experimental[9 38 40] and 6 were cluster-randomised controlled trials (cluster RCTs)[10 31 33 34 41]. Twelve studies were from South Asia, 3 from SSA and 1 from LAC. These studies included evaluated different methods of empowerment.

Intimate partner violence (IPV): Five cross-sectional surveys[27-30 32] and 1 cluster RCT[31] reported this outcome. One survey[28] showed a significant 24% (95%CI 1·05-1·44) increase in odds of IPV among microfinance clients compared to non-clients. On the other hand, the cluster RCT[31] demonstrated a significant decrease in IPV (adjusted risk ratio 0.45, 95%CI 0.23-0.91) and another survey[32] similarly

For peer review only - http://bmjopen.bmj.com/site/about/guidelines.xhtml

showed reductions among clients of the two MFIs studied (OR=0.44, 95%CI 0.28-0.70 and OR=0.30, 95%CI 0.18-0.51). Dalal *et al*[29] found that microfinance clients with secondary and higher education were 2-3 times more likely to experience IPV than comparable non-clients (p=<0·001), while wealthier clients were twice as likely to experience IPV than comparable non-clients (p=<0·001); there were no changes in exposure to IPV amongst the least educated and poorest groups. This finding was confirmed by Murshid et al.[30] who also analysed the data from the same Bangladeshi Demographic Health Survey of 2007.

A meta-analysis was not conducted due to high heterogeneity (I^2=91·3%). This heterogeneity could have arisen because the threshold for reporting violence or the framing of the question may have differed between settings. The cluster RCT[31] was different both in design and in the add-on life skills training, which may have introduced further heterogeneity. The association between IPV and microfinance is therefore inconclusive.

Decision making ability: Eight studies were included for this outcome, 5 from South Asia[33 36 37 39 42] and 3 from SSA[34 41], with 4 cluster RCTs[33-35 41], and 4 cross-sectional surveys[36 37 39 42]. This measure analysed a change from not being involved in decision making to being an active participant in household decisions. The outcome measures used were diverse and therefore unsuitable for meta-analysis. The results have been tabulated in more detail in Supplement 4 and include participation in financial and other household decisions (e.g. children's education and healthcare). Just over half the studies[33 37 39 42] showed a slightly higher degree of participation in certain household decisions by microfinance clients compared to non-clients. The other studies did not report any statistically significant changes. The impact of microfinance on women's decision making is therefore inconclusive.

***Freedom to travel (mobility)*:** In the one study that assessed mobility, non-clients were more mobile than clients in one region, but in the two other regions studied the reverse was true[38]. No formal statistical comparisons between groups were presented.

Children's nutrition

Five studies, 4 from SSA[7 8 43 44] and 1 from India[45], evaluated the effect of microfinance on children's nutrition. Three[8 43 44] were cross-sectional surveys, 1 was a quasi-experimental study with a 16 month follow-up period[7] while 1 was a cluster randomised controlled trial[45]. Two studies[7 44] included only children between 6-36 months of age while the other 3 included children under five years.

For peer review only - http://bmjopen.bmj.com/site/about/guidelines.xhtml

Doocy *et al* reported that children of women non-clients were 79% more likely to be wasted than children of clients (OR=1·79 95%CI 0·87-3·79)[8]. However, Friesen et al reported increased wasting among children of clients compared to non-clients (OR=1·15 95%CI 0·30-4·43)[44]. Neither association was statistically significant. As the baseline group used was different and there were no raw data available, it was not possible to recalculate the ORs for pooling by meta-analysis.

One cross-sectional study found that the prevalence of malnutrition, based on HAZ-scores, was lower amongst children of microfinance clients than those of non-clients[43]. A longitudinal study measured HAZ, WAZ and BAZ-scores every four months for 16 months[7]. The authors demonstrated a mean difference in WAZ scores of 0.28 at 8 to 12 months in favour of the intervention group and significant but smaller differences at four months and 16 months. At 16 months, HAZ-scores were significantly higher in the intervention group with a mean difference of 0·19 between the two groups. Meta-analysis was not possible as the studies used different statistical measures to present their results.

Ojha et al. reported that in a cross-sectional survey conducted 18 months after random allocation to received immediate microfinance vs. delayed microfinance (after 18 months), 0-5 year old children in the villages that received immediate microfinance had a significantly better WHZ compared to children in the villages that did not receive microfinance with a mean difference of 0.35 SD[45]. They found similar differences in WAZ, and prevalence of wasting, underweight and moderate and severe malnutrition as measured by mid-upper arm circumferences but there was no difference in HAZ or prevalence of stunting between the two groups.

Publication Bias: A funnel plot found no evidence of publication bias in the studies that reported the impact of microfinance on IPV (Egger's test *p*-value=0·106). The possibility of publication bias could not be assessed for the other outcomes.

DISCUSSION

Summary of evidence: Table 3 summarises the impact of microfinance across the three outcome domains based on the quantitative and qualitative syntheses described above.

Table 3: Summary of Results of the Review

For peer review only - http://bmjopen.bmj.com/site/about/guidelines.xhtml

Outcome	Summary of impact of microfinance
Use of contraception	Women participating in microfinance schemes were significantly more likely to report using contraception.
Female empowerment	
Intimate partner violence	Conflicting results, with some studies reporting increased and others decreased IPV in microfinance participants.
Decision making ability	Most studies showed no effect but a minority showed a significant positive effect on some areas of decision-making.
Mobility	No statistically significant impact.
Overall empowerment score	Positive impact in two studies with mixed results and no change in two others.
Children's nutrition	Positive impact in three of five studies, with no difference found in the remaining studies.

Seventeen of the 27 studies included in the review were from South Asia. This may limit the generalisability of the findings of this review to other geographical regions. However, this was expected as 84% of all microfinance clients are to be found in South Asia[46]. Other included studies, nine from Africa and one from Latin America, are geographically heterogeneous but catered to women of a similar economic background. These populations are potentially comparable for the purposes of a study looking at the impact of microfinance. However, it is of note that the review includes populations from a wider geographical range, with diverse political, cultural and social backgrounds.

Proposed mechanisms

Microfinance (whilst primarily improving economic stability) might empower women and improve child nutrition though a number of mechanisms. A small source of income, which is available primarily to the woman in the household, could increase the "bargaining power" of female participants, in household decision making. Peer support and shared learning from other participants might have a similar effect. We have chosen the outcome measures most likely to reflect this increased bargaining power, including a woman's decisions about contraception and her self-reported empowerment. Furthermore, that women are often the primary household decision-makers on issues such as buying food (which will affect child nutrition) and on access to healthcare for children. These factors could interact to enable women to overcome social, cultural and economic barriers that affect their status (Figure 3)

For peer review only

For peer review only - http://bmjopen.bmj.com/site/about/guidelines.xhtml

Contraceptive Use

Where individual-level data were available, the odds of reporting contraceptive use were higher in women participating in microfinance compared to those who did not. It has been argued that the women who self-select to join microfinance groups are more empowered than other women and this may in itself increase their likelihood of using contraception[4]. However, by comparing reported use in this group before and after the intervention[23 25], it is possible to demonstrate a positive effect attributable to microfinance, even with an inherent empowered state.

Markers of female empowerment

Intimate Partner Violence

Gender-related violence is known to be most commonly perpetuated by a person close to the woman, usually an intimate partner[46]. Although a reduction in IPV is one of the expected benefits of empowerment of women through microfinance, empowerment may also enable women to report more IPV, thus increasing the rate of reported IPV. One cluster RCT[31] reported a reduction in IPV among microfinance clients. However, the combined microfinance with life skills training may have resulted in an intervention group different from the standard client therefore limiting the generalisability of their findings. The authors of this study argued that their training empowered the women to reveal IPV, therefore reducing underreporting[31]. Underreporting of IPV is common in many studies due to its sensitive nature[47]. Studies used trained local female interviewers to limit underreporting, but despite this, the response rate to IPV questions in one study was only 41%[29]. Furthermore, women participating in microfinance may want to only highlight positive impacts of the intervention and not reveal any IPV. This raises ethical concerns that studies may fail to detect violence where it is actually present[47].

Studies that have reported increase in IPV linked to microfinance programmes[29] have also argued that microfinance loans may have caused more economic stress in the family leading to greater occasions for conflict. Some authors explain this as the "status inconsistency theory" where in status differentials may lead to dysfunctional behaviour when and individual who expects to have a higher status in a relationship is threatened by the increase in the status of another[30]. Previously there may have been fewer conflicts as the man would have managed finances single-handedly while with empowerment, the wife becomes involved in these decisions, generating more occasions where conflict leading to IPV could occur.

For peer review only

For peer review only - http://bmjopen.bmj.com/site/about/guidelines.xhtml

Decision Making Ability

In most cases, the decision-making ability of women participating in microfinance was not significantly different from that of non-clients. However, most studies analysed women's perceived decision-making ability, which may be different to their actual decision-making capability. In addition, composite indices of decision-making ability make it hard to untangle any impact of microfinance on decisions which are typically male-dominated (such as child marriage and education) and decisions which are traditionally less so (such as those related to the purchase of food).

Children's nutrition

Three studies[8 44 45] reported a lower likelihood of severe acute malnutrition in children of women participating in microfinance compared to non-participants, including one that showed a statistically significant reduction in malnutrition[45]. Combining microfinance with nutritional education, as was the case in one study[7], showed improvement in nutritional status in children of participating care-givers than non-participating care-givers. However, it is then difficult to isolate the specific effect of microfinance. In one SHG study[43] no attempt was made to adjust for other variables, such as household resources or education status, which may be a source of confounding.

Additionally, the inclusion of HAZ scores as a measure of nutritional status[43 44] in a cross-sectional study may be misleading. In their cluster randomised trial, Ojha et al. report an improvement in all other indices of malnutrition other than HAZ and stunting after an 18 month period[45]. Height-for-age measures the effect of poor nutrition on the growth of a child. Growth faltering is slow in reversal and requires a longer follow-up period to detect[48]. It may be more prudent to use acute measures of malnutrition such as wasting (WHZ) which are likely to be more sensitive to change in nutritional status over shorter periods.

Strengths and limitations

Five comprehensive databases were searched in this review, including a large economic database. The use of multiple indicators to measure women's empowerment and children's nutrition also served to broaden the search to reduce the likelihood of missing relevant articles. The selection was carried out independently by two authors without any language restrictions, particularly important given the geographical regions studied.

For peer review only - http://bmjopen.bmj.com/site/about/guidelines.xhtml

The models used to deliver microfinance services varied across included studies. Some combined microfinance with education on family planning[23], life skills[31] or health, nutrition and entrepreneurial skills[7], which makes it difficult to evaluate the effect of microfinance alone. Although all interventions were taken to be similar for the purposes of this review, it is possible that the way the microfinance services were provided may have influenced the outcome. Given the small number of interventions of each type reviewed here, it is not possible to suggest a model of microfinance that is superior to others in terms of social performance.

In general, the most common source of bias in studies of the social impact of microfinance is selection bias, as participants *self-select* to either participate or not participate in the programme. Although, it may be argued that it would be difficult to randomise people to microfinance as the intervention may not be desired by all; therefore measuring effectiveness in those who did not desire it to begin with, may be problematic. Whilst a cluster RCT might guard against selection bias, a recent study[10] highlighted the current challenge in achieving randomisation due to the widespread diffusion of microfinance in some regions of South Asia leading to difficulties in identifying unexposed control clusters. Therefore, we included non-randomised studies in this review in order to not limit the evidence considered. The non-randomised studies included dealt with self-selection bias in two main ways, using either panel data in a quasi-experimental design or propensity score matching (PSM). However, additional analysis in of one of the studies included in this review suggested that the reduction in intimate partner violence demonstrated using conventional statistical methods did not hold when PSM was used[28].

Due to the lack of high quality randomised controlled trials in this field, the vast majority of studies included in this study were cross-sectional. As a study design, cross-sectional studies do not provide the strongest level of evidence. Analysis of quasi-experimental and panel data studies proved difficult as there is currently no universally acceptable quality assessment tool. The use of the Cochrane Risk-of-Bias tool in this instance may have introduced an over-or under-estimation of the risk of bias and, consequently, the quality assessment of the study.

There was a lack of homogeneity in the measures used to assess social performance of microfinance particularly that of decision making ability which varied from study to study which may account for the conflicting outcomes. The average follow-up period of the studies included was three years. An alternative explanation for their statistically non-significant findings is that the observation period may have not been long enough to detect any change or may have missed any fleeting changes that

For peer review only - http://bmjopen.bmj.com/site/about/guidelines.xhtml

For peer review only

occurred before the follow up survey. While changes in some measures of children's malnutrition may be detectable within three years, changes in other outcomes requiring a shift in cultural and social norms may take much longer

Conclusions

In conclusion, our findings suggest that for the types of microfinance interventions assessed in this study, there may be an association between microfinance and increasing contraceptive use, improving female empowerment and better children's nutrition. However, as only 6 of 27 studies included in this review were randomised trials any conclusions about direct causation must be guarded. However, the wide diversity in reported outcomes, study design, statistical methods and microfinance models makes it difficult to synthesise evaluation data statistically. Thus further studies are required to evaluate the social performance of microfinance. Such studies could focus on some of the many unanswered questions such as the impact of microfinance on specific standardised measures of children's health and women's wellbeing such that the findings could be compared across populations. The lack of this evidence is highlighted by the paucity of good quality studies included in this review. Other unanswered questions include the long term impact of microfinance on communities and designing studies focused on potential harm. The design of future studies requires effective and clearly described randomisation, harmonisation of appropriate outcome measures and avoidance of confounders. Incorporating evaluation methods at the onset of a microfinance programme could help address many of the weaknesses identified here. While this may not be practical in areas where microfinance is fully established, areas with an increasing number of microfinance programmes, for example sub-Saharan Africa, would benefit.

For peer review only

ACKNOWLEDGEMENTS

We thank Magdalena Opazo Breton and Gabriella Zapata for their assistance in translating manuscripts written in Spanish and Portuguese.

We thank Dr Rajeev Kamal and his team at the A N Sinha Institute of Social Sciences, Patna, India for organising the public involvement workshop.

CONTRIBUTION STATEMENT

WG, LS, SO and AS conceived and designed the study. WG and LS independently carried out the title, abstract and full text screening and quality assessment. WG conducted the meta-analyses and wrote the first draft of the paper. SS updated the search in 2018 and completed the updated title and

For peer review only - http://bmjopen.bmj.com/site/about/guidelines.xhtml

abstract screening. The updated full text screening was performed by SO, SS and WG. All authors critically revised subsequent drafts, and have approved the final version.

For peer review only

For peer review only - http://bmjopen.bmj.com/site/about/guidelines.xhtml

REFERENCES

1. Ledgerwood J, Earne J, Nelson C. *The New Microfinance Handbook: A Financial Market System*: The International Bank for Reconstruction and Development/The World Bank, 2013.
2. Robinson MS, Marguerite S, Fidler, et al. The microfinance revolution : sustainable finance for the poor 2001; 1(Report number: 23250). http://documents.worldbank.org/curated/en/226941468049448875/sustainable-finance-for-the-poor.
3. Schurmann AT, Johnston HB. The group-lending model and social closure: microcredit, exclusion, and health in Bangladesh. J Health Popul Nutr 2009;**27**(4):518-27.
4. Goldberg N. Measuring the Impact of Microfinance: Taking Stock of What We Know. Washinton DC, 2005.
5. Pitt MM, Khandker SR. The impact of group-based credit programs on poor households in Bangladesh: Does the gender of participants matter? J Polit Econ 1998;**106**(5):958-96.
6. MicroRate. The State of Microfinance Investment 2013. Secondary The State of Microfinance Investment 2013 2013. http://www.microrate.com/media/downloads/2013/11/MicroRate-The-State-of-Microfinance-Investment-2013.pdf.
7. Marquis GS, Colecraft EK, Sakyi-Dawson O, et al. An Integrated Microcredit, Entrepreneurial Training, and Nutrition Education Intervention Is Associated with Better Growth Among Preschool-Aged Children in Rural Ghana. J Nutr 2015;**145**(2):335-43.
8. Doocy S, Teferra S, Norell D, et al. Credit program outcomes: coping capacity and nutritional status in the food insecure context of Ethiopia. Soc Sci Med 2005;**60**(10):2371-82.
9. Pitt MM, Khandker SR, Shahidur R, et al. Does Micro-Credit Empower Women : Evidence from Bangladesh. Policy Research Working Paper;No. 2998. . 2003.
10. Banerjee A, Duflo E, Glennerster R, et al. The miracle of microfinance? Evidence from a randomized evaluation. American Economic Journal: Applied Economics 2015;**7**(1):22-53.
11. Bank W. World Bank: countries. Secondary World Bank: countries 2016. http://www.worldbank.org/en/country.
12. Bank W. Poverty Overview. Secondary Poverty Overview 2018. http://worldbank.org/en/topic/poverty/overview.
13. Odell K. Measuring the Impact of Microfinance: Taking another look. 2010. https://grameenfoundation.org/blog/measuring-impact-microfinance-taking-another-look#.W1iROdVKiUk.
14. WHO. Monitoring Maternal, Newborn and Child Health: Understanding Key Progress Indicators. 2011.
15. Murray CJL, Salomon JA, Mathers C, et al. Summary Measures of Population Health: Concepts, Ethics, Measurement and Applications. 2002. http://apps.who.int/iris/bitstream/handle/10665/42439/9241545518.pdf?sequence=1&isAllowed=y.
16. Project UM. Investing in Development: A Practical Plan to Achieve the Millennium Development Goals. . 2005.
17. Rustagi P. Situation of Women in South Asia: Some Dimensions. . 2015.
18. ECMIA. Violence and Indigenous Women. 2013. http://www.ncdsv.org/images/Ecmia-Chirapaq_Violence-and-Indigenous-Women_3-2013.pdf.
19. Kabeer N. Resources, agency, achievements: Reflections on the measurement of women's empowerment. Dev Change 1999;**30**(3):435-64.
20. CDC. Intimate Partner Violence. Secondary Intimate Partner Violence 2018. https://www.cdc.gov/violenceprevention/intimatepartnerviolence/.
21. *Cochrane Handbook for Systematic Reviews of Interventions*: The Cochrane Collaboration, 2011.

For peer review only - http://bmjopen.bmj.com/site/about/guidelines.xhtml

22. Wells GA, Shea B, O'Connell, et al. The Newcastle-Ottawa Scale (NOS) for assessing the quality of nonrandomised studies in meta-analyses. Secondary The Newcastle-Ottawa Scale (NOS) for assessing the quality of nonrandomised studies in meta-analyses. http://www.ohri.ca/programs/clinical_epidemiology/oxford.asp.
23. Desai J, Tarozzi A. Microcredit, Family Planning Programs, and Contraceptive Behavior: Evidence From a Field Experiment in Ethiopia. Demography 2011;**48**(2):749-82.
24. Pitt MM, Khandker SR. Household and Intrahousehold Impact of the Grameen Bank and Similar Targeted Credit Programs in Bangladesh. 1996. http://documents.worldbank.org/curated/en/385431468740690795/pdf/multi-page.pdf.
25. Steele F, Amin S, Naved RT. Savings/credit group formation and change in contraception. Demography 2001;**38**(2):267-82.
26. Murshid NS, Ely GE. Microfinance participation and contraceptive use: does control over resources matter? J Public Health (Oxf) 2017;**39**(2):e27-e32.
27. Ahmed SM. Intimate partner violence against women: Experiences from a woman-focused development programme in Matlab, Bangladesh. Journal of Health Population and Nutrition 2005;**23**(1):95-101.
28. Bajracharya A, Amin S. Microcredit and Domestic Violence in Bangladesh: An Exploration of Selection Bias Influences. Demography 2013;**50**(5):1819-43.
29. Dalal K, Dahlstrom O, Timpka T. Interactions between microfinance programmes and non-economic empowerment of women associated with intimate partner violence in Bangladesh: a cross-sectional study. Bmj Open 2013;**3**(12).
30. Murshid NS, Akincigil A, Zippay A. Microfinance Participation and Domestic Violence in Bangladesh: Results From a Nationally Representative Survey. J Interpers Violence 2016;**31**(9):1579-96.
31. Pronyk PM, Hargreaves JR, Kim JC, et al. Effect of a structural intervention for the prevention of intimate-partner violence and HIV in rural South Africa: a cluster randomised trial. Lancet 2006;**368**(9551):1973-83.
32. Schuler SR, Hashemi SM, Riley AP, et al. Credit programs, patriarchy and men's violence against women in rural Bangladesh. Soc Sci Med 1996;**43**(12):1729-42.
33. Angelucci M, Karlan D, Zinman J. Microcredit Impacts: Evidence from a Randomized Microcredit Program Placement Experiment by Compartamos Banco. Am Econ J-Appl Econ 2015;**7**(1):151-82.
34. Beaman L, Karlan D, Thuysbaert B. Saving for a (not So) Rainy Day: A Randomized Evaluation of Savings Groups in Mali. 2014. http://www.nber.org/papers/w20600.pdf.
35. Karlan D, Savonitto B, Thuysbaert B, et al. Impact of savings groups on the lives of the poor. P Natl Acad Sci USA 2017;**114**(12):3079-84.
36. Mohindra KS, Haddad S, Narayana D. Can microcredit help improve the health of poor women? Some findings from a cross-sectional study in Kerala, India. Int J Equity Health 2008;**7**.
37. Montgomery H, Weiss J. Can Commercially-oriented Microfinance Help Meet the Millennium Development Goals? Evidence from Pakistan. World Dev 2011;**39**(1):87-109.
38. Rahman S, Junankar PN, Mallik G. Factors influencing women's empowerment on microcredit borrowers: a case study in Bangladesh. J Asia Pac Econ 2009;**14**(3):287-303.
39. Sharif NR. Microcredit programs and women's decision-making status: Further evidence from Bangladesh. Can J Dev Stud 2004;**25**(3):465-80.
40. Swain RB, Wallentin FY. Does microfinance empower women? Evidence from self-help groups in India. International Review of Applied Economics 2009;**23**(5):541-56.
41. Tarozzi A, Desai J, Johnson K. The Impacts of Microcredit: Evidence from Ethiopia. Am Econ J-Appl Econ 2015;**7**(1):54-89.
42. Zaman H. Assessing the Impact of Micro-Credit on Poverty and Vulnerability in Bangladesh. Policy,. 1999.

For peer review only - http://bmjopen.bmj.com/site/about/guidelines.xhtml

http://documents.worldbank.org/curated/en/630461468741915080/Assessing-the-impact-of-micro-credit-on-poverty-and-vulnerability-in-Bangladesh.
43. Abubakari A, Sadik Bb, Keisan Y. Impact of Village Savings and Loans Associations on the Nutritional Status of Under-Five Children: A Case Study in the Sissala West District of Upper West Region. Pakistan Journal of Nutrition 2014;**13**(7):309-96.
44. Friesen V, Marquis GS, Fallon K, et al. Tackling poverty through private sector microcredit programs in Ghana: does infant and young child nutrition improve? McGill University, 2012.
45. Ojha S, Szatkowski L, Sinha R, et al. PO5 Effects of the rojiroti microfinance programme on nutrition in very poor children under five in india. Archives of Disease in Childhood 2017;**102**(A3).
46. Devries KM, Mak JYT, Garcia-Moreno C, et al. The Global Prevalence of Intimate Partner Violence Against Women. Science 2013;**340**(6140):1527-28.
47. WHO. Ethical and Safety Recommendations for Research on Domestic Violence Against Women. 2001. http://www.who.int/gender/violence/womenfirtseng.pdf.
48. Shrimpton R, Victora CG, de Onis M, et al. Worldwide timing of growth faltering: implications for nutritional interventions. Pediatrics 2001;**107**(5):E75.

For peer review only

For peer review only - http://bmjopen.bmj.com/site/about/guidelines.xhtml

Figure legends:

Figure 1. PRISMA flow chart

Figure 2. Fixed effects meta-analysis of effect of microfinance participation on women reporting contraceptive use.

Figure 3. Theory of change model linking microfinance to women's wellbeing and children's nutrition

For peer review only

For peer review only - http://bmjopen.bmj.com/site/about/guidelines.xhtml

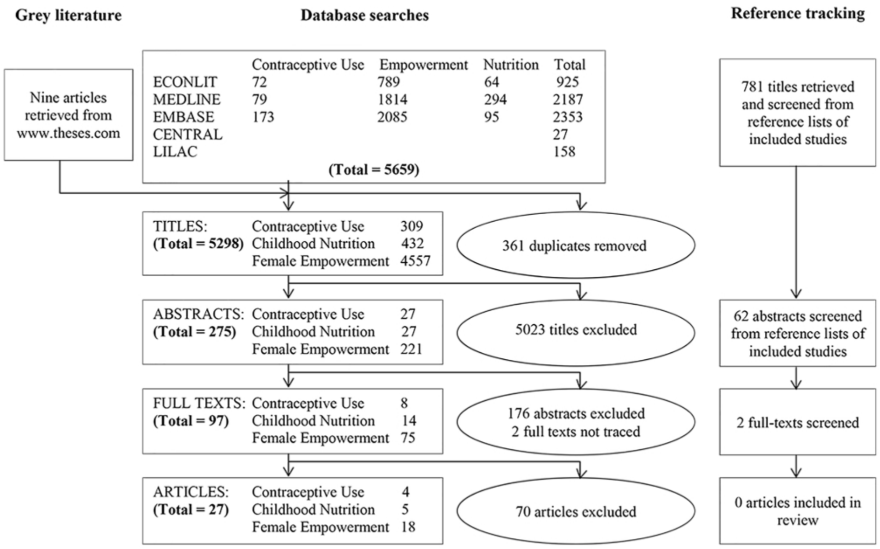

Figure 1. PRISMA flow chart

287x179mm (300 x 300 DPI)

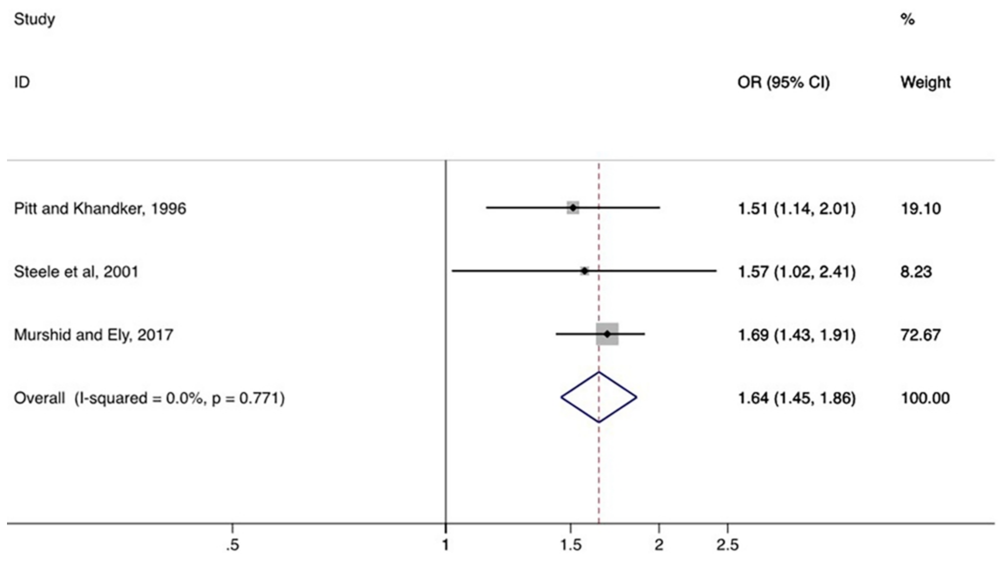

Figure 2.Fixed effects meta-analysis of effect of microfinance participation on women reporting contraceptive use

287x179mm (300 x 300 DPI)

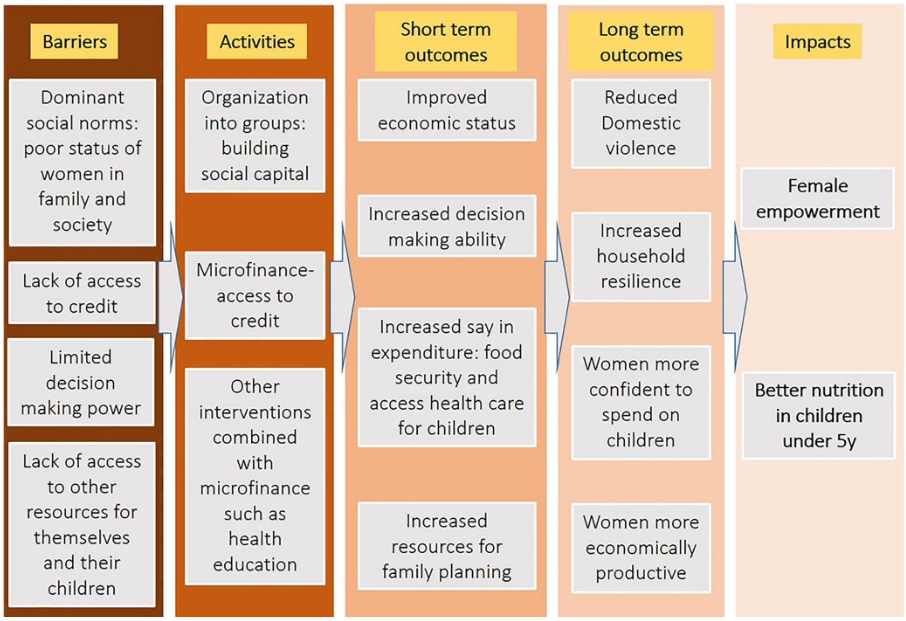

Figure 3. Theory of change model linking microfinance to women's wellbeing and children's nutrition

287x179mm (300 x 300 DPI)

Is microfinance associated with changes in women's wellbeing and childhood nutrition? A systematic review and meta-analysis

Wanjiku Gichuru[1] MPH (International Health), Shalini Ojha[2] PhD, Alan R Smyth[3] MD, Lisa Szatkowski PhD[1]

[1]University of Nottingham, Division of Epidemiology and Public Health, Clinical Sciences Building, Nottingham City Hospital, Nottingham, UK, NG5 1PB

[2]University of Nottingham, Division of Graduate Entry Medicine, Derby Medical School, Derby, DE22 3DT

[3]Child Health, Obstetrics and Gynaecology, E Floor, East Block, Queen's Medical Centre, Nottingham, UK, NG7 2UH

Corresponding author: shalini.ojha@nottingham.ac.uk

For peer review only

For peer review only - http://bmjopen.bmj.com/site/about/guidelines.xhtml

Supplementary material

Supplement 1: Search Terms

1. MEDLINE SEARCH STRATEGY

Contraceptive Use

1. (microfinanc* or microcredit or microloan or "micro-financ*" or "micro-credit" or "micro-loan" or "micro financ*" or "micro credit" or "micro loan" or "small loan" or "small lend" or "micro enterpris*" or "micro enterpreneur").mp. [mp=title, abstract, original title, name of substance word, subject heading word, keyword heading word, protocol supplementary concept word, rare disease supplementary concept word, unique identifier]
2. (random* or "randomi*ed control trial" or "randomi*ed cluster trial" or study or analys* or cohort or "cross section*" or "cross-section*" or survey or " pre test and post test" or "pre-test and post-test" or "before and after" or "interrupted time series" or "time series" or "time-series" or "time points").mp. [mp=title, abstract, original title, name of substance word, subject heading word, keyword heading word, protocol supplementary concept word, rare disease supplementary concept word, unique identifier]
3. ("contraceptive*" or "contraception" or "reproductive" or gynaecolog* or gynecolog* or "birth control" or fertility).mp. [mp=title, abstract, original title, name of substance word, subject heading word, keyword heading word, protocol supplementary concept word, rare disease supplementary concept word, unique identifier]
4. exp contraceptive behavior/
5. 3 or 4
6. economics/ or financial support/
7. 1 or 6
8. 2 and 5 and 7
9. limit 8 to (humans and yr="1990 -Current")

Female Empowerment

1. economics/ or financial support/
2. (microfinanc* or microcredit or microloan or "micro-financ*" or "micro-credit" or "micro-loan" or "micro financ*" or "micro credit" or "micro loan" or "small loan" or "small lend" or "micro enterpris*" or "micro enterpreneur").mp. [mp=title, abstract, original title, name of substance word,

For peer review only - http://bmjopen.bmj.com/site/about/guidelines.xhtml

subject heading word, keyword heading word, protocol supplementary concept word, rare disease supplementary concept word, unique identifier]

3. ("health" or "outcome" or "evaluat*" or "intervention" or "impact" or "result*" or "effect*").mp. [mp=title, abstract, original title, name of substance word, subject heading word, keyword heading word, protocol supplementary concept word, rare disease supplementary concept word, unique identifier]

4. (random* or "randomi*ed control trial" or "randomi*ed cluster trial" or study or analys* or cohort or "cross section*" or "cross-section*" or survey or " pre test and post test" or "pre-test and post-test" or "before and after" or "interrupted time series" or "time series" or "time-series" or "time points").mp. [mp=title, abstract, original title, name of substance word, subject heading word, keyword heading word, protocol supplementary concept word, rare disease supplementary concept word, unique identifier]

5. ("gender" or "female" or "gender violence" or "assault" or "women" or "woman" or "women's empowerment" or "empowerment" or " women's rights" or "gender equality" or "intimate partner violence" or " travel* without permission" or "girl* education" or "school enrollment" or "school enrolment" or "infanticide").mp. [mp=title, abstract, original title, name of substance word, subject heading word, keyword heading word, protocol supplementary concept word, rare disease supplementary concept word, unique identifier]

6. 1 or 2

7. 3 or 5

8. 4 and 6 and 7

9. limit 8 to (humans and yr="1990 -Current")

Nutrition

1. (microfinanc* or microcredit or microloan or "micro-financ*" or "micro-credit" or "micro-loan" or "micro financ*" or "micro credit" or "micro loan" or "small loan" or "small lend" or "micro enterpris*" or "micro enterpreneur").mp. [mp=title, abstract, original title, name of substance word, subject heading word, keyword heading word, protocol supplementary concept word, rare disease supplementary concept word, unique identifier]

2. economics/ or exp financial support/

3. Child Nutrition Disorders/ or Nutrition Disorders/ or Nutrition Surveys/

4. (nutrition or malnutrition or undernutrition or under-nutrition or "MUAC" or "mid-upper arm circumference" or "Z score*" or Z-scores or "weight-for-age" or stunting or "weight-for-height" or "weight for age" or "weight for height" or underweight or “height for age” or “height-for-age” or

For peer review only - http://bmjopen.bmj.com/site/about/guidelines.xhtml

wasting or “whz” or wasting).mp. [mp=title, abstract, original title, name of substance word, subject heading word, keyword heading word, protocol supplementary concept word, rare disease supplementary concept word, unique identifier]

5. (random* or "randomi*ed control trial" or "randomi*ed cluster trial" or study or analys* or cohort or "cross section*" or "cross-section*" or survey or "pre test and post test" or "pre-test and post-test" or "before and after" or "interrupted time series" or "time series" or "time-series" or "time points").mp. [mp=title, abstract, original title, name of substance word, subject heading word, keyword heading word, protocol supplementary concept word, rare disease supplementary concept word, unique identifier]

6. 1 or 2

7. 3 or 4

8. 6 and 7

9. 5 and 8

10. limit 9 to (yr="1990 -Current")

II EMBASE SEARCH STRATEGY

Contraceptive Use

1. (microfinanc* or microcredit or microloan or "micro-financ*" or "micro-credit" or "micro-loan" or "micro financ*" or "micro credit" or "micro loan" or "small loan" or "small lend" or "micro enterpris*" or "micro enterpreneur").mp. [mp=title, abstract, heading word, drug trade name, original title, device manufacturer, drug manufacturer, device trade name, keyword]

2. (random* or "randomi*ed control trial" or "randomi*ed cluster trial" or study or analys* or cohort or "cross section*" or "cross-section*" or survey or "pre test and post test" or "pre-test and post-test" or "before and after" or "interrupted time series" or "time series" or "time-series" or "time points").mp. [mp=title, abstract, heading word, drug trade name, original title, device manufacturer, drug manufacturer, device trade name, keyword]

3. exp finance/

4. 1 or 3

5. ("contraceptive*" or "contraception" or "reproductive" or gynaecolog* or gynecolog* or "birth control" or fertility).mp. [mp=title, abstract, heading word, drug trade name, original title, device manufacturer, drug manufacturer, device trade name, keyword]

6. exp contraceptive behavior/

7. 5 or 6

For peer review only - http://bmjopen.bmj.com/site/about/guidelines.xhtml

8. 4 and 7

9. 2 and 8

10. limit 9 to (human and yr="1990 -Current")

Female Empowerment

1. (microfinanc* or microcredit or microloan or "micro-financ*" or "micro-credit" or "micro-loan" or "micro financ*" or "micro credit" or "micro loan" or "small loan" or "small lend" or "micro enterpris*" or "micro enterpreneur").mp. [mp=title, abstract, heading word, drug trade name, original title, device manufacturer, drug manufacturer, device trade name, keyword]

2. ("health" or "outcome" or "evaluat*" or "intervention" or "impact" or "result*" or "effect*").mp. [mp=title, abstract, heading word, drug trade name, original title, device manufacturer, drug manufacturer, device trade name, keyword]

3. (random* or "randomi*ed control trial" or "randomi*ed cluster trial" or study or analys* or cohort or "cross section*" or "cross-section*" or survey or "pre test and post test" or "pre-test and post-test" or "before and after" or "interrupted time series" or "time series" or "time-series" or "time points").mp. [mp=title, abstract, heading word, drug trade name, original title, device manufacturer, drug manufacturer, device trade name, keyword]

4. ("gender" or "female" or "gender violence" or "assault" or "women" or "woman" or "women's empowerment" or "empowerment" or " women's rights" or "gender equality" or "intimate partner violence" or " travel* without permission" or "girl* education" or "school enrollment" or "school enrolment" or "infanticide").mp. [mp=title, abstract, heading word, drug trade name, original title, device manufacturer, drug manufacturer, device trade name, keyword]

5. exp finance/

6. 1 or 5

7. 2 or 4

8. 3 and 6 and 7

9. limit 8 to (human and yr="1990 -Current")

10. 1 and 3 and 7

11. limit 10 to (human and yr="1990 -Current")

Nutrition

1. (microfinanc* or microcredit or microloan or "micro-financ*" or "micro-credit" or "micro-loan" or "micro financ*" or "micro credit" or "micro loan" or "small loan" or "small lend" or "micro

For peer review only

enterpris*" or "micro enterpreneur").mp. [mp=title, abstract, heading word, drug trade name, original title, device manufacturer, drug manufacturer, device trade name, keyword]

2. Child Nutrition Disorders/ or Nutrition Disorders/ or Nutrition Surveys/

3. (nutrition or malnutrition or undernutrition or under-nutrition or "MUAC" or "mid-upper arm circumference" or "Z score*" or Z-scores or "weight-for-age" or stunting or "weight-for-height" or "weight for age" or "weight for height" or underweight or “height for age” or “height-for-age” or wasting or “whz” or wasting).mp. [mp=title, abstract, heading word, drug trade name, original title, device manufacturer, drug manufacturer, device trade name, keyword]

4. (random* or "randomi*ed control trial" or "randomi*ed cluster trial" or study or analys* or cohort or "cross section*" or "cross-section*" or survey or "pre test and post test" or "pre-test and post-test" or "before and after" or "interrupted time series" or "time series" or "time-series" or "time points").mp. [mp=title, abstract, heading word, drug trade name, original title, device manufacturer, drug manufacturer, device trade name, keyword]

5. 2 or 3

6. exp finance/

7. 1 or 6

8. 5 and 7

9. 4 and 8

10. limit 9 to (human and yr="1990 -Current")

III ECONLIT SEARCH STRATEGY

Female Empowerment

microfinanc* or microcredit or microloan or "micro-financ*" or "micro-credit" or "micro-loan" or "micro financ*" or "micro credit" or "micro loan" or "small loan" or "small lend" or "micro enterpris*" or "micro entrepreneur”

AND

"health" or "outcome" or "evaluat*" or "intervention" or "impact" or "result*" or "effect*" or "gender" or "female" or "gender violence" or "assault" or "women" or "woman" or "women's empowerment" or "empowerment" or " women's rights" or "gender equality" or "intimate partner violence" or " travel* without permission" or "girl* education" or "school enrollment" or "school enrolment" or "infanticide"

AND

For peer review only - http://bmjopen.bmj.com/site/about/guidelines.xhtml

random* or "randomi*ed control trial" or "randomi*ed cluster trial" or study or analys* or cohort or "cross section*" or "cross-section*" or survey or " pre test and post test" or "pre-test and post-test" or "before and after" or "interrupted time series" or "time series" or "time-series" or "time points”

Contraceptive Use

microfinanc* or microcredit or microloan or "micro-financ*" or "micro-credit" or "micro-loan" or "micro financ*" or "micro credit" or "micro loan" or "small loan" or "small lend" or "micro enterpris*" or "micro entrepreneur”

AND

random* or "randomi*ed control trial" or "randomi*ed cluster trial" or study or analys* or cohort or "cross section*" or "cross-section*" or survey or "pre test and post test" or "pre-test and post-test" or "before and after" or "interrupted time series" or "time series" or "time-series" or "time points”

AND

"contraceptive*" or "contraception" or "reproductive" or gynaecolog* or gynecolog* or "birth control" or fertility

Nutrition

microfinanc* or microcredit or microloan or "micro-financ*" or "micro-credit" or "micro-loan" or "micro financ*" or "micro credit" or "micro loan" or "small loan" or "small lend" or "micro enterpris*" or "micro entrepreneur”

AND

nutrition OR malnutrition OR undernutrition OR under-nutrition OR underweight OR "MUAC" OR "mid-upper arm circumference" OR stunting OR "weight-for-age" OR "height for age" OR "height-for-age" OR wasting OR whz OR "Z score"

AND

random* or "randomi*ed control trial" or "randomi*ed cluster trial" or study or analys* or cohort or "cross section*" or "cross-section*" or survey or "pre test and post test" or "pre-test and post-test" or "before and after" or "interrupted time series" or "time series" or "time-series" or "time points"

IV CENTRAL

microfinanc* or microcredit or microloan or "micro-financ*" or "micro-credit" or "micro-loan" or "micro financ*" or "micro credit" or "micro loan" or "small loan" or "small lend" or "micro enterpris*" or "micro enterpreneur"

For peer review only - http://bmjopen.bmj.com/site/about/guidelines.xhtml

V LILAC

Microfinan$ OR microcredit$ OR microenterprise$ OR microentrepreneur$ OR microemp$

OR

(micro AND (enterprise$ ORcredit$ OR entrepreneur$ OR finan$ OR empres$ OR companhia$))

OR

Index microfinanzas

For peer review only

For peer review only - http://bmjopen.bmj.com/site/about/guidelines.xhtml

Supplement 2: REVISED NEWCASTLE-OTTAWA SCALE ADAPTED FOR CROSS-SECTIONAL STUDIES

Selection: (Maximum 5 stars) /6

1) Representativeness of the sample: **

a) Truly representative of the average in the target population. ** (all subjects or random sampling)

b) Somewhat representative of the average in the target population. * (non-random sampling)

c) Selected group of users.

d) No description of the sampling strategy.

2) Sample size:

a) Justified and satisfactory. *

b) Not justified.

3) Non-respondents:

a) Comparability between respondents and non-respondents characteristics is established, and the response rate is satisfactory. *

b) The response rate is unsatisfactory, or the comparability between respondents and non-respondents is unsatisfactory.

c) No description of the response rate or the characteristics of the responders and the non-responders.

4) Ascertainment of the exposure (risk factor):**

a) Validated – based on individual exposure. **

b) Non-validated measurement tool, but the tool is available or described – based on group exposure e.g. village level.*

c) No description of the measurement tool.

Comparability: (Maximum 2 stars) – /2

1) The subjects in different outcome groups are comparable, based on the study design or analysis. Confounding factors are controlled.

a) The study controls for the most important factors – age, education level, social status (select one). * *

b) The study displays data on the above factors comparing intervention and non-intervention groups but does not adjust*

For peer review only - http://bmjopen.bmj.com/site/about/guidelines.xhtml

For peer review only

c) No data on above factors collected

Outcome: (Maximum 2 stars) /3

1) Assessment of the outcome:

a) Assessment through self-reported anonymised questionnaires or blinded independent assessors. **

b) Record linkage. **

c) Systematic assessment without blinding or independent assessors and self-reported through interviewer. *

d) No description

2) Statistical test: -

a) The statistical test used to analyze the data is clearly described and appropriate, and the measurement of the association is presented, including confidence intervals and the probability level (p value). *

b) The statistical test is not appropriate, not described or incomplete.

Total # of stars: /11

This scale has been adapted from the Newcastle-Ottawa Quality Assessment Scale for cohort studies to perform a quality assessment of cross-sectional studies for this systematic review.

For peer review only

For peer review only - http://bmjopen.bmj.com/site/about/guidelines.xhtml

Supplement 3: Studies Excluded at Full-Text Screening

Reason For Exclusion	Number excluded
Contraceptive Use	
No results for outcome of interest	3
No comparison group included in the study	1
Childhood Nutrition	
No results for outcome of interest	4
Inappropriate measure provided at result stage (pooled result)	1
Study results already presented in another included article	1
Not traced in print and online editions of journal referenced	3
Female Empowerment	
No non-economic outcome presented in results	29
Outcome of interest only presented for intervention group but not for comparison group	1
No comparison group included in the study	12
Comparison group included but were also exposed to the intervention in some capacity	4
Exposure included other credit sources as well as microfinance	1
No empirical quantitative data presented (theoretical framework)	3
Study results already presented in another included article (including critiques of existing studies)	3
Study protocol only, no results provided	1
Not traced in print and online editions of journal referenced	3
Primary exposure of interest not microfinance	2
Not traced in the British library catalogue and other sources	2

For peer review only - http://bmjopen.bmj.com/site/about/guidelines.xhtml

Supplement 4: Impact of Microfinance on Household Decision Making Agency among Female Clients

Study	Measure of Decision Making Agency	Statistical measure of effect used	Results<br>(*p*=*p-value, n*=sample size)	Direction of effect
Angelucci *et al, 2015*[37]	Proportion of women who participate in any financial decision<br>Number of household issues women have a say on	Regression coefficients (SE)	0.008 (0.003)<br>*p*<0.01 *n*=12183<br>0.071 (0.030)<br>*p*<0.05 *n*=12379	Positive
Beaman *et al, 2014*[38]	Proportion of women free to decide about a) food expenses b) education expenses c) business.<br><br>d) Standardised index of intra-household decision making power derived from 3 individual measures	Regression coefficients (SE) for outcomes a-c<br><br>Regression coefficient (SE) for change in standardised index d (i.e. change in deviations from mean)	-0.006 (0.016) n=5425<br>0.010 (0.014) n=4440<br>0.012 (0.020) n=4180<br>0.02 (0.03) *n*=5425	No significant change
Mohindra *et al, 2008*[39]	Decision-making agency based on at least one situation of male decision making versus no male decision making in: seeking health care of a family member; daily household expenditures; child's education at school; family planning; voting in an election.	Adjusted odds ratio for early joiner (>2 years membership) compared to non-clients	0.90 (95%CI 0.53-1.74)<br>n=928	No significant change
Montgomery & Weiss, 2011[40]	Women between 15-40 asked about their involvement in family decisions regarding:<br>Child's schooling<br>Child's marriage<br>Whether to have another child<br>Repair/construction of house<br>Sale-purchase of livestock<br>Borrowing money	Logit, SE and ORs for female clients compared to non-clients	0.22 (0.30) OR=1.25<br>0.45 (0.34) OR=1.57<br>-0.01 (0.51) OR=0.99<br>0.36 (0.40) OR=1.43<br>-0.12 (0.57) OR=0.88<br>0.96 (0.38) OR=2.62**<br>-1.16 (1.08) OR=0.31<br>-0.60 (0.60) OR=0.55<br>**significant at 5% level *n*=2876	Positive change in involvement in decisions regarding borrowing money.<br>No significant changes in other domains.

For peer review only - http://bmjopen.bmj.com/site/about/guidelines.xhtml

	Woman's participation in community political activity<br>Woman's decision to work outside home			
Sharif, 2004[42]	Degree of participation in decisions regarding:<br>Daily food purchases<br>Large purchases e.g. house, furniture<br>Health expenditure<br>Education of children<br>Marriage of children and social events<br>Fertility<br>Five point ranking given for each domain, 1 being least able, to 5, able to make decisions on her own	Means and standard deviation, Wilcoxon Z statistic and significance for difference between groups	Clients Non-clients<br>4.2 (1.15) 3.8 (1.45)<br>Z=1.83, $p<0.05$<br>3.1 (0.78) 2.7 (0.99)<br>Z=2.43, p<0.05<br>3.1 (0.87) 2.9 (0.91)<br>Z=0.68<br>3.2 (0.82) 2.9 (0.81)<br>Z=1.43, p<0.05<br>2.9 (0.61) 2.9 (0.67)<br>Z=2.14<br>2.9 (0.39) 2.9 (0.54)<br>Z=0.39	Positive change in decisions on purchase of food, large purchases and education of children
Tarozzi *et al*, *2015*[44]	Standardised index of fraction of decision across 20 domains women involved in:<br>All issues<br>Economic issues<br>(standardised using mean and SD of the outcome estimated from control areas at endline)	Regression coefficients (SEs) for change in standardised index (i.e. change in deviations from mean)	-0.043 (0.030)<br>n=10500 women<br>-0.038 (0.032)<br>n=10497 women	No significant change
Zaman, 1999[45]	Decision making agency:<br>If owns poultry % that can sell poultry independently<br>If owns livestock % that can sell livestock independently<br>If owns jewellry % that can sell jewellry independently<br>If has savings % can use savings independently	Coefficient estimates	-0.103 (*n*=980)<br>-0.178 (*n*= 103)<br>0.017 (*n*= 694)<br>-0.345*** (*n*=379)<br><br>***significant at 1% level	Positive change only in decisions on use of savings

For peer review only - http://bmjopen.bmj.com/site/about/guidelines.xhtml

PROSPERO

International prospective register of systematic reviews

National Institute for Health Research

Is microfinance associated with changes in women's empowerment and childhood nutrition, and does this vary by geographical region? A systematic review and meta-analysis

Wanjiku J Gichuru, Lisa Szatkowski, Alan Smyth, Shalini Ojha

Citation

Wanjiku J Gichuru, Lisa Szatkowski, Alan Smyth, Shalini Ojha. Is microfinance associated with changes in women's empowerment and childhood nutrition, and does this vary by geographical region? A systematic review and meta-analysis. PROSPERO 2015 CRD42015026018 Available from: http://www.crd.york.ac.uk/PROSPERO/display_record.php?ID=CRD42015026018

Review question

Through systematic review and, if possible, meta-analysis assess whether microfinance programmes are associated with changes in female empowerment and the wellbeing of women over the age of 15 years

Through systematic review and, if possible, meta-analysis assess whether microfinance programmes are associated with changes in use of a contraception method among women of reproductive age

Through systematic review and, if possible, meta-analysis assess whether microfinance programmes are associated with changes in childhood nutrition and whether this varies by the sex of the child

Searches

The Cochrane Central Register of Controlled Trials (CENTRAL),

Ovid MEDLINE,

EMBASE,

Latin American and Caribbean Health Sciences (LILACS),

ECONLIT.

An attempt will be made to access unpublished studies and dissertations through a search of grey literature through www.thesis.com.

The search will be limited to studies carried out after 1990.

No language restrictions will be imposed.

Types of study to be included

Cross-sectional surveys, cohort studies, controlled before-and-after studies, interrupted time series, quasi-experimental studies, randomised and non-randomised control/cluster trials.

Condition or domain being studied

Women's empowerment as measured by intimate partner violence, decision making agency, mobility and distinctly as uptake of a contraceptive method. Empowerment of women may also be linked to improved childhood nutrition. This will be measured by weight-for-age Z-scores, height-for-age Z-scores, weight-for-height Z scores and mid-upper arm circumference.

Participants/population

Inclusion: Women above the age of fifteen and children under-five for the outcome on childhood nutrition.

Exclusion: Men, children above five years

Intervention(s), exposure(s)

Intervention: Microfinance schemes defined as a combination of savings and credit services offered without physical collateral to a population thought to be poor or otherwise vulnerable through any organisation or institution.

The provider may be non-profit, e.g. NGO, self-help group (SHG), community-based organisation or microfinance bank, or a for-profit micro-finance institution, e.g., commercial bank.

Studies having an additional intervention will also be considered, provided that the primary intervention is microfinance.

Comparator(s)/control

Populations without any microfinance services or the same population prior to receiving microfinance. In studies with more than one comparator group, the group without microfinance will be considered as the main comparator.

Context

Developing countries in South Asia, sub Saharan Africa and Latin America and the Caribbean as defined by the Word Bank

Primary outcome(s)

1. Use of contraception

2. Childhood nutrition measured as the rate of malnutrition in girls and boys under-five years of age

3. Female empowerment and well-being

Timing and effect measures

1. Use of contraception method

2. Weight-for age Z score, Height-for-age Z score, Weight-for-height Z score, mid-upper arm circumference

3. Intimate partner violence (IPV), decision making agency,mobility

Secondary outcome(s)

None

Data extraction (selection and coding)

The search will be conducted and subsequent papers reviewed for eligibility independently by two researchers in three stages; title, abstract and full-text.

For peer review only - http://bmjopen.bmj.com/site/about/guidelines.xhtml

A data extraction form will be completed for each selected study by one researcher under the following sub-headings; publication details, study details, nature of study, intervention and results. The data extraction forms will then be reviewed by the second researcher. This is to be used in further analysis and synthesis of the data.

Any disparities will be solved be mutual consensus between the two primary researchers. If this is not possible, the input of the third researcher will be sought.

Risk of bias (quality) assessment

The selected studies will be assessed for risk of bias by two researchers using the Cochrane Collaboration's tool for assessing risk of bias in randomised controlled trials and for quality by the Newcastle-Ottawa Quality Assessment Scale in non-randomised studies.

Any disparities will be resolved be mutual consensus between the two primary researchers. If this is not possible, the input of the third researcher will be sought.

Strategy for data synthesis

Outcome measures will be extracted from the studies and used in the meta-analyses. The studies providing an appropriate measure of effect will be weighted using a quality rating system and then stratified by quality score. A descriptive analysis will be done for studies providing quantitative outcome measures not suitable for meta-analysis.

A fixed-effects or a random-effects model will be used in pooling of the data and a suitable method of estimating variance in studies will be applied. The summary estimate of the effect size will be done in each stratum according to quality score, i.e. high, medium and low quality score, and statistical tests (I-squared) used to check for heterogeneity.

Analysis of subgroups or subsets

A sub-group analysis of the measures of effect chosen will be done according to region to detect any variations between regions. The three regions will be geographically specified as Sub-Saharan Africa, South Asia and South America. The results will be presented by tables within the text of the review or if possible in forest plots in the meta-analysis

Contact details for further information

Dr Gichuru

wanjiku.gichuru@gmail.com

Organisational affiliation of the review

University of Nottingham

www.nottingham.ac.uk

Review team members and their organisational affiliations

Dr Wanjiku J Gichuru. University of Nottingham
Dr Lisa Szatkowski. University of Nottingham
Professor Alan Smyth. University of Nottingham
Dr Shalini Ojha. University of Nottingham

Anticipated or actual start date

20 April 2015

Anticipated completion date

08 December 2015

Funding sources/sponsors

The Commonwealth Scholars and Fellows Scheme funded the Masters' course of which this review formed part of the dissertation

Conflicts of interest

None known

Language

English

Country

England

Stage of review

Review_Completed_not_published

Subject index terms status

Subject indexing assigned by CRD

Subject index terms

Female; Humans; Nutritional Status; Power (Psychology)

Date of registration in PROSPERO

09 September 2015

Date of publication of this version

12 January 2016

Revision note for this version

Update to reflect the completion of the review.

Details of any existing review of the same topic by the same authors

Stage of review at time of this submission

Stage	Started	Completed
Preliminary searches	Yes	Yes
Piloting of the study selection process	Yes	Yes
Formal screening of search results against eligibility criteria	Yes	Yes
Data extraction	Yes	Yes
Risk of bias (quality) assessment	Yes	Yes

For peer review only - http://bmjopen.bmj.com/site/about/guidelines.xhtml

Stage	Started	Completed
Data analysis	Yes	Yes

Revision note

Update to reflect the completion of the review.

Versions

09 September 2015

12 January 2016

PROSPERO
This information has been provided by the named contact for this review. CRD has accepted this information in good faith and registered the review in PROSPERO. CRD bears no responsibility or liability for the content of this registration record, any associated files or external websites.

PRISMA 2009 Checklist

Section/topic	#	Checklist item	Reported on page #
**TITLE**			
Title	1	Identify the report as a systematic review, meta-analysis, or both.	1
**ABSTRACT**			
Structured summary	2	Provide a structured summary including, as applicable: background; objectives; data sources; study eligibility criteria, participants, and interventions; study appraisal and synthesis methods; results; limitations; conclusions and implications of key findings; systematic review registration number.	2
**INTRODUCTION**			
Rationale	3	Describe the rationale for the review in the context of what is already known.	4
Objectives	4	Provide an explicit statement of questions being addressed with reference to participants, interventions, comparisons, outcomes, and study design (PICOS).	5
**METHODS**			
Protocol and registration	5	Indicate if a review protocol exists, if and where it can be accessed (e.g., Web address), and, if available, provide registration information including registration number.	5
Eligibility criteria	6	Specify study characteristics (e.g., PICOS, length of follow-up) and report characteristics (e.g., years considered, language, publication status) used as criteria for eligibility, giving rationale.	5
Information sources	7	Describe all information sources (e.g., databases with dates of coverage, contact with study authors to identify additional studies) in the search and date last searched.	6
Search	8	Present full electronic search strategy for at least one database, including any limits used, such that it could be repeated.	Supplement 1
Study selection	9	State the process for selecting studies (i.e., screening, eligibility, included in systematic review, and, if applicable, included in the meta-analysis).	7
Data collection process	10	Describe method of data extraction from reports (e.g., piloted forms, independently, in duplicate) and any processes for obtaining and confirming data from investigators.	7
Data items	11	List and define all variables for which data were sought (e.g., PICOS, funding sources) and any assumptions and simplifications made.	6
Risk of bias in individual studies	12	Describe methods used for assessing risk of bias of individual studies (including specification of whether this was done at the study or outcome level), and how this information is to be used in any data synthesis.	Supplement 2
Summary measures	13	State the principal summary measures (e.g., risk ratio, difference in means).	7

For peer review only - http://bmjopen.bmj.com/site/about/guidelines.xhtml

PRISMA 2009 Checklist

Synthesis of results	14	Describe the methods of handling data and combining results of studies, if done, including measures of consistency (e.g., I^2) for each meta-analysis.	7

Section/topic	#	Checklist item	Reported on page #
Risk of bias across studies	15	Specify any assessment of risk of bias that may affect the cumulative evidence (e.g., publication bias, selective reporting within studies).	14
Additional analyses	16	Describe methods of additional analyses (e.g., sensitivity or subgroup analyses, meta-regression), if done, indicating which were pre-specified.	7
**RESULTS**			
Study selection	17	Give numbers of studies screened, assessed for eligibility, and included in the review, with reasons for exclusions at each stage, ideally with a flow diagram.	7 [Figure 1]
Study characteristics	18	For each study, present characteristics for which data were extracted (e.g., study size, PICOS, follow-up period) and provide the citations.	8 – 11 Table 2
Risk of bias within studies	19	Present data on risk of bias of each study and, if available, any outcome level assessment (see item 12).	8 – 11 [Table 2]
Results of individual studies	20	For all outcomes considered (benefits or harms), present, for each study: (a) simple summary data for each intervention group (b) effect estimates and confidence intervals, ideally with a forest plot.	12 - 14
Synthesis of results	21	Present results of each meta-analysis done, including confidence intervals and measures of consistency.	12 [Figure 2]
Risk of bias across studies	22	Present results of any assessment of risk of bias across studies (see Item 15).	14
Additional analysis	23	Give results of additional analyses, if done (e.g., sensitivity or subgroup analyses, meta-regression [see Item 16]).	
**DISCUSSION**			
Summary of evidence	24	Summarize the main findings including the strength of evidence for each main outcome; consider their relevance to key groups (e.g., healthcare providers, users, and policy makers).	15
Limitations	25	Discuss limitations at study and outcome level (e.g., risk of bias), and at review-level (e.g., incomplete retrieval of identified research, reporting bias).	17
Conclusions	26	Provide a general interpretation of the results in the context of other evidence, and implications for future research.	18
**FUNDING**			
Funding	27	Describe sources of funding for the systematic review and other support (e.g., supply of data); role of funders for the systematic review.	2

For peer review only - http://bmjopen.bmj.com/site/about/guidelines.xhtml

PRISMA 2009 Checklist

From: Moher D, Liberati A, Tetzlaff J, Altman DG, The PRISMA Group (2009). Preferred Reporting Items for Systematic Reviews and Meta-Analyses: The PRISMA Statement. PLoS Med 6(6): e1000097. doi:10.1371/journal.pmed1000097

For more information, visit: **www.prisma-statement.org**.

For peer review only

For peer review only - http://bmjopen.bmj.com/site/about/guidelines.xhtml
